# Novel diagnostic and therapeutic techniques reveal changed metabolic profiles in recurrent focal segmental glomerulosclerosis

**DOI:** 10.1038/s41598-021-83883-w

**Published:** 2021-02-25

**Authors:** Janina Müller-Deile, George Sarau, Ahmed M. Kotb, Christian Jaremenko, Ulrike E. Rolle-Kampczyk, Christoph Daniel, Stefan Kalkhof, Silke H. Christiansen, Mario Schiffer

**Affiliations:** 1 Department of Nephrology and Hypertension, Friedrich-Alexander-University (FAU) Erlangen-Nuremberg, Erlangen, Germany; 2 Fraunhofer Institute for Ceramic Technologies and Systems IKTS, Dresden, Germany; 3 Leuchs Emeritus Group, Max Planck Institute for the Science of Light, Erlangen, Germany; 4 Institute for Nanotechnology and Correlative Microscopy eV INAM, Forchheim, Germany; 5 Department of Anatomy and Histology, Faculty of Veterinary Medicine, Assiut University, Asyût, Egypt; 6 Institute of Optics, Information and Photonics, Friedrich-Alexander-University (FAU) Erlangen-Nuremberg, Erlangen, Germany; 7 Department Molecular Systems Biology, Helmholtz Centre for Environmental Research, Leipzig, Germany; 8 Department of Nephropathology, Friedrich-Alexander-University (FAU) Erlangen-Nuremberg, Erlangen, Germany; 9 Institute for Bioanalysis, University of Applied Sciences Coburg, Coburg, Germany; 10 Department of Molecular Systems Biology, Helmholtz-Centre for Environmental Research-UFZ, Leipzig, Germany; 11 Physics Department, Freie Universität Berlin, Berlin, Germany

**Keywords:** Mass spectrometry, Glomerular diseases, Focal segmental glomerulosclerosis, Raman spectroscopy, Kidney, Glomerulus, Podocytes, Diagnostic markers

## Abstract

Idiopathic forms of Focal Segmental Glomerulosclerosis (FSGS) are caused by circulating permeability factors, which can lead to early recurrence of FSGS and kidney failure after kidney transplantation. In the past three decades, many research endeavors were undertaken to identify these unknown factors. Even though some potential candidates have been recently discussed in the literature, “the” actual factor remains elusive. Therefore, there is an increased demand in FSGS research for the use of novel technologies that allow us to study FSGS from a yet unexplored angle. Here, we report the successful treatment of recurrent FSGS in a patient after living-related kidney transplantation by removal of circulating factors with CytoSorb apheresis. Interestingly, the classical published circulating factors were all in normal range in this patient but early disease recurrence in the transplant kidney and immediate response to CytoSorb apheresis were still suggestive for pathogenic circulating factors. To proof the functional effects of the patient’s serum on podocytes and the glomerular filtration barrier we used a podocyte cell culture model and a proteinuria model in zebrafish to detect pathogenic effects on the podocytes actin cytoskeleton inducing a functional phenotype and podocyte effacement. We then performed Raman spectroscopy in the < 50 kDa serum fraction, on cultured podocytes treated with the FSGS serum and in kidney biopsies of the same patient at the time of transplantation and at the time of disease recurrence. The analysis revealed changes in podocyte metabolome induced by the FSGS serum as well as in focal glomerular and parietal epithelial cell regions in the FSGS biopsy. Several altered Raman spectra were identified in the fractionated serum and metabolome analysis by mass spectrometry detected lipid profiles in the FSGS serum, which were supported by disturbances in the Raman spectra. Our novel innovative analysis reveals changed lipid metabolome profiles associated with idiopathic FSGS that might reflect a new subtype of the disease.

## Introduction

Primary focal segmental glomerulosclerosis (FSGS) is a rare disease with an estimated incidence of about 7 per 1 million^1^. However, it is still one of the most prevalent nephropathies causing end-stage renal disease^2,3^. FSGS is a morphologic pattern of glomerular injury defined by the presence of sclerosis in parts (segmental) of some (focal) glomeruli and global podocyte foot process effacement in all glomeruli. Subclasses of FSGS include primary (idiopathic), genetic and secondary forms. Despite sharing certain clinical and histologic features, these subclasses differ in management and prognosis^4^. Distinguishing between different forms of FSGS is important to avoid unnecessary immunosuppressive treatments in case of secondary forms and most genetic forms^5^. Secondary FSGS is the consequence of other underlying chronic diseases such as diabetes, obesity, HIV, hypertension and other glomerular diseases. Genetic FSGS results from defects in podocyte genes and is often steroid resistant. Mutations in more than 30 recessive or dominant genes were identified for monogenic forms of steroid resistant nephrotic syndrome. Recessive mutations in nephrin (NPHS1), podocin (NPHS2), CD2 associated protein (CD2AP), laminin β2 (LAMB2), integrin, phospholipase C epsilon 1 (PLCE1), protein tyrosine phosphatase receptor type O (PTPRO) and alpha 3 (ITGA3) are only some examples. Dominant mutations include actin alpha 4 (ACTN4), inverted formin 2 (INF2), anillin (ANLN), transient receptor potential cation channel (TRPC6) and Wilms tumor 1 (WT1)^6^.

In contrast, idiopathic FSGS-forms are most likely caused by circulating permeability factors. Soluble urokinase plasminogen activator receptor (suPAR), cardiotrophin-like cytokine factor-1 (CLCF1) and sCD25 have been postulated as such permeability factors in FSGS^7–10^. However, it remains to be determined whether these factors are truly pathogenic, specific, sensitive and reproducible in all patients and just like the genetic forms it is likely that this disease category represents only a subgroup of diseases and that not only one but several different or a combination of circulatory factors exist.

The incidence of FSGS is increasing across the entire age spectrum worldwide^11^. Rapid onset of heavy proteinuria in a nephrotic range, hypoalbuminemia and edema are typical symptoms and sings of primary FSGS. FSGS non-responsive to steroid treatment has a high risk to progress to end stage kidney disease and second line treatment is often not effective or disrupted due to side effects. Moreover, patients who progress to end stage kidney disease and receive a kidney transplant have a 20 to 30% risk of FSGS recurrence in their kidney graft^11,12^.

There is an unmet clinical need for early and specific biomarkers to detect FSGS and to predict its prognosis and response to therapy. So far, diagnostic and therapeutic decisions are based on unspecific markers as proteinuria, serum creatinine as well as renal histology. However, even by histology the actual disease of the patients might be misinterpreted, as FSGS is a focal disease vulnerable to sampling errors in the biopsy^13^.

Here we report CytoSorb apheresis as a novel therapeutic approach to bring recurrent FSGS in remission in a patient that was nonresponsive to immunosuppressive therapy and plasmapheresis. We report an innovative zebrafish model to detect the presence of circulating permeability factors causing FSGS that might be used as non-invasive diagnostic test in the future. In order to characterize idiopathic FSGS on cell, serum and tissue level, we performed Raman spectroscopy in podocytes treated with the patient’s serum and control serum, in < 50 kDa serum fractions for FSGS patient and four control patients and on renal biopsies of a patient at the time of kidney transplantation, at the time of FSGS recurrence and in a biopsy with minimal change disease. Vertical differences in Raman intensities represent changes in component concentrations. Horizontal shifts in Raman signals are due to slight differences in molecular vibrations caused by modifications in the molecule composition. Both, Raman signals corresponding to changes in lipid concentrations as well as lipid composition were found in FSGS patient samples at the time of disease recurrence. Assignment of Raman bands in the FSGS biopsy to published metabolites corresponded to mass spectrometry findings. Serum metabolome analysis was able to detect metabolic changes in the patient’s serum during the active phase of the disease. Previously described circulating factor were detectable in the normal range. Therefore, we hypothesize that these metabolomic signatures indirectly reflects the effects of the unknown circulating factors in the kidney or that these metabolome changes might even represent the pathogenic components themselves.

## Results

### CytoSorb apheresis to treat for treatment resistant and recurrent FSGS

**Figure 1:**
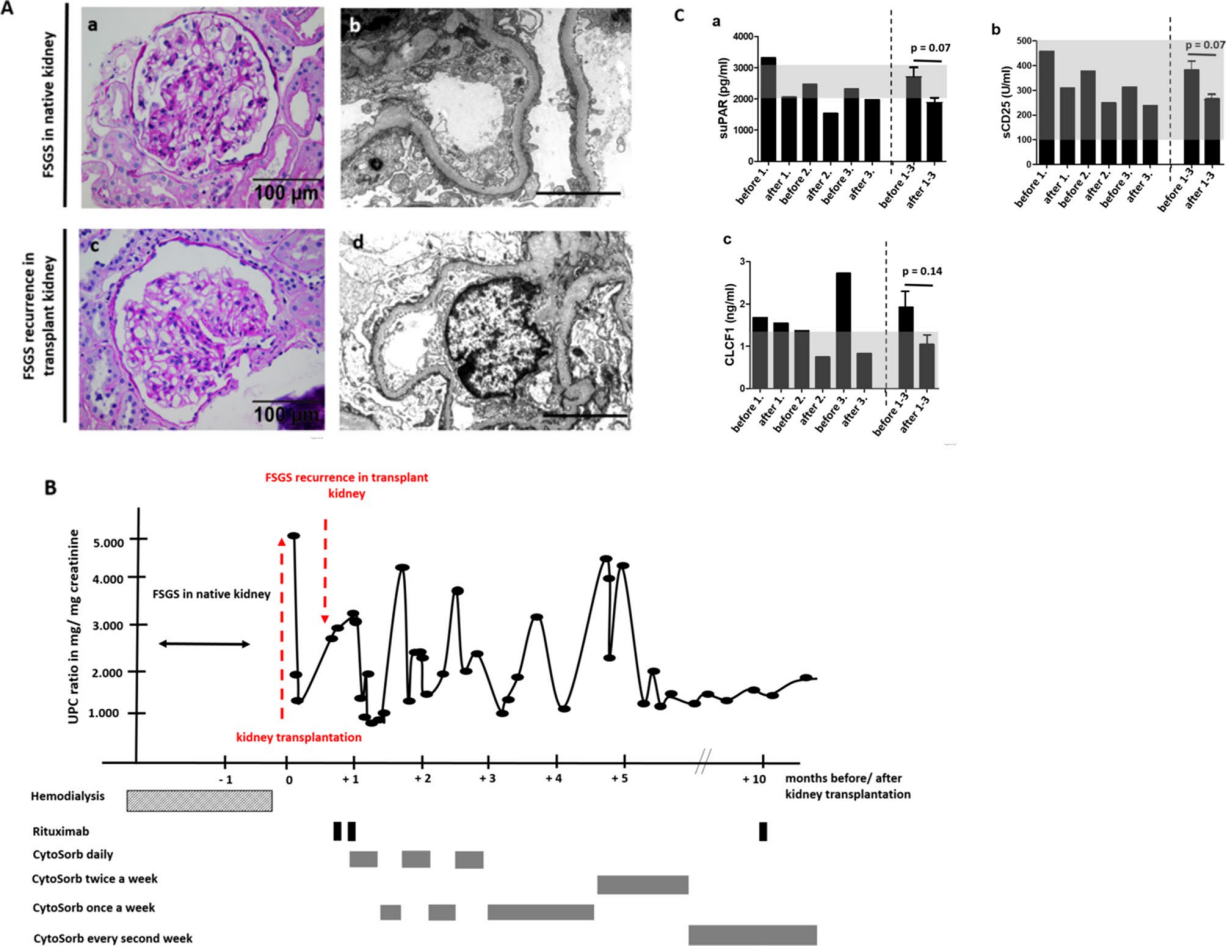
CytoSorb apheresis to treat therapy resistant and early recurrent FSGS. (**A**)—(a) PAS staining of native kidney biopsy of the patient at the time of initial diagnosis of FSGS. Scale bar = 100 µm. (b) Transmission electron microscopy picture of native kidney biopsy of the patient at the time of initial diagnosis of FSGS. Scale bar = 1 µm. (c) PAS staining of transplant kidney biopsy of the patient at the time of the diagnosis of recurrence of podocytopathy. Scale bar = 100 µm. (d) Transmission electron microscopy picture of transplant kidney biopsy of the patient at the time of the diagnosis of recurrence of podocytopathy. Scale bar = 1 µm. (**B**) Illustration of clinical course of the patient. Proteinuria measured as urine-protein-creatinine-ratio (UPC-ratio) is given in black dots and lines. Time points of kidney transplantation, transplant kidney biopsy, Rituximab treatment and CytoSorb apheresis schedule are illustrated. (**C**) Measurements for suPAR (a), sCD25 (b) and CLCF1 (c) in serum samples of the patient before and after first, second and third CytoSorb apheresis. The last two columns depict the mean of measurements for the parameters before and after apheresis 1–3. Normal reference levels are shown in gray. Differences before and after CytoSorb apheresis were not significant for suPAR, CLCF1 and sCD25.

Treatment options for FSGS caused by circulating factors are limited and often unsuccessful. We report a 24-year-old woman with nephrotic syndrome and biopsy proven FSGS in her native kidneys (Fig. 1Aa,b). Clinical history for secondary FSGS was negative and genetic testing did not reveal reported mutations in podocyte genes. Different treatment regimens including steroids, cyclosporine A, mycophenolate mofetil, rituximab and even plasmapheresis were unsuccessful and within 12 months after initial diagnosis, the patient developed end stage renal disease and was started on hemodialysis. Two years later, the patient received a living-related donor kidney transplantation from her mother. Primary renal transplant function was very good. Serum creatinine decreased to 1.14 mg/dl 10 days after transplantation and no significant proteinuria was detectable. However, 1 month after transplantation, the patient developed proteinuria with a urine-creatinine-protein-ratio (UPC-ratio) of 3 g/mg creatinine. Transplant kidney biopsy showed podocytopathy with podocyte effacement, which confirmed recurrent disease (Fig. 1Ac,d). The patient received intravenous corticoid treatment and two doses of rituximab (1 g) within 2 weeks. Despite of this therapy proteinuria did not respond. Given the early recurrence of significant proteinuria shortly after transplantation, we hypothesized the presence of a circulating permeability factor causing the disease. Due to the severity and rapid progression of the primary disease, the early recurrence after transplantation and the past medical history where the disease in the native kidneys was refractory to established treatment regimens, we decided to treat the patient with CytoSorb apheresis as a compassionate use approach. Initially, CytoSorb apheresis was performed daily for 4 days. Proteinuria immediately decreased to 327 mg/g creatinine and CytoSorb apheresis treatment schedule was reduced to once a week (Fig. 1B). However, proteinuria rapidly relapsed to 4235 mg/g creatinine so that CytoSorb treatment frequency was increased again until the patient developed clinical remission. However, a week later proteinuria again relapsed (3686 mg/g creatinine) (Fig. 1B). A third time CytoSorb apheresis was performed daily and brought the patient back into remission. After additional therapy with rituximab, CytoSorb treatment was tapered to once a week. Since then, the patient was in remission with proteinuria around 0.5 mg/g creatinine and serum creatinine around 1.5 mg/dl under CytoSorb treatment every second week. We follow this patient now for more than 2 years with an excellent transplant function and have so far not been able to wean the patient completely from CytoSorb apheresis. All attempts to increase the time between the treatment cycles have failed. Of note, if the patient is missing a session (e.g. due to a holiday) proteinuria immediately increases and rituximab treatments had to continue in 6 months intervals (data not shown). We measured proposed circulating permeability factors in the patient’s serum before and after first, second and third CytoSorb apheresis. suPAR (< 4000 pg/ml) and sCD25 (< 500 U/ml) were in normal range before the first apheresis and decreased not significantly in response to CytoSorb treatments (Fig. 1Ca,b). Serum concentration of CLCF1 was 1.67 ng/ml at the time of FSGS recurrence and decreased to 0.82 ng/ml after the 3rd CytoSorb treatment (Fig. 1Cc). In serum samples from healthy controls, concentrations of CLCF1 were between 0 ng/ml (66% of controls) and 1.2 ng/ml. Cathepsin L (undetectable) and IL6 (< 2 pg/ml) were also not elevated in the patient’s serum (data not shown).

### Ex vivo tests to detect morphological changes in podocytes caused by FSGS serum

Even though published circulating factors were in normal range or not detectable in our patient, the early disease recurrence after kidney transplantation and the immediate response to CytoSorb treatment were still suggestive for a disease-causing factor in the patient’s circulation. To screen for functional impacts of the patient’s serum on podocytes we used a cell-based ex vivo screening model. Differentiated human podocytes were cultured in the presence of serum from our patient with FSGS at the time of recurrence in transplant kidney and before the first CytoSorb treatment (FSGS serum) and compared to podocytes cultured in the presence of serum of a transplanted FSGS patient without a relapse in the transplant (CTRL serum) as well as serum from a patient with membranous glomerulonephritis (MGN serum) for three and 6 h. For quantification of changes in actin cytoskeleton of podocytes, we used a scoring system published earlier (cell type A–D, see “Methods”)^14–16^.

**Figure 2:**
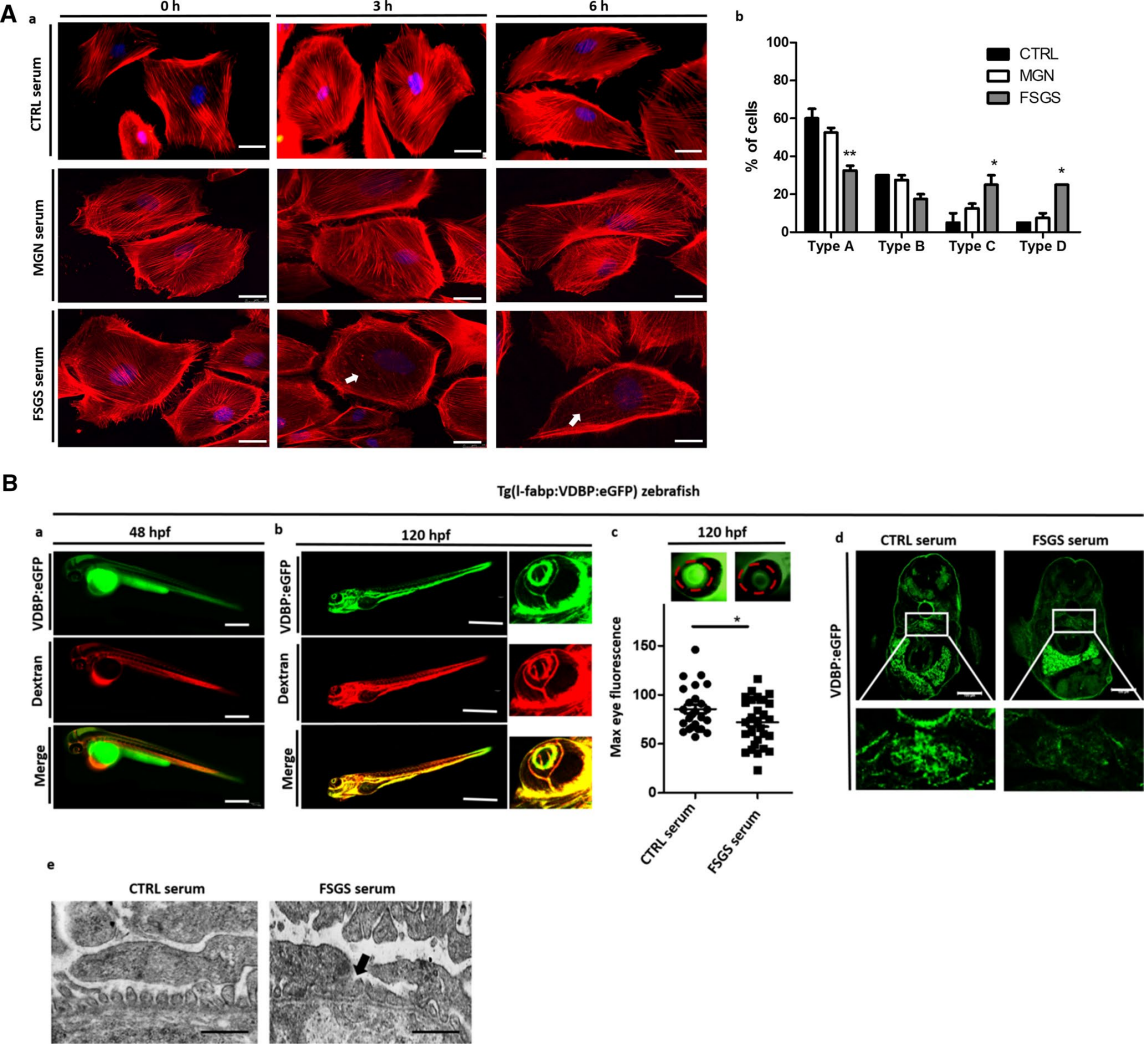
Cell culture and zebrafish assay to detect morphological and functional effects of unknown circulating permeability factors in FSGS. (**A**)—(a) Representative images of cultured differentiated human podocytes treated with 10% serum from a healthy control (CTRL serum), the patient with recurrent FSGS in the kidney transplant and a patient with membranous glomerulonephritis (incubation for 0 h, 3 h and 6 h). Cells were stained with phalloidin for cytoskeleton labeling. White arrows indicate cytoskeleton rearrangement. Scale bar = 25 µm. (b) Quantification of stress fiber formation in podocytes after treatment with different sera of patients. Type A: more than 90% of cell area filled with thick cables; type B: at least 2 thick cables running under nucleus and rest of cell area filled with fine cables; type C: no thick cables, but some cables present; type D: no cables visible in the central area of the cell. (**B**) Zebrafish assay for the detection of circulating permeability factors. (a) Representative image of a transgenic Tg(l-fabp:VDBP:eGFP) zebrafish larvae (VDBP:eGFP) injected with serum: dextran texas red into the zebrafish circulation (Dextran c.v. injection) at 48 hpf. Proper injection leads to red fluorescence of the zebrafish vascularization. Expression of the green fluorescent vitamin D binding protein just started. Scale bar = 500 µm. (b) At 120 hpf injected Tg(l-fabp:VDBP:eGFP) zebrafish express green fluorescent vitamin D binding protein (VDBP:eGFP) in the circulation. Red fluorescent serum: dextran mixture is still detectable in the circulation (dextran) and merges with the green fluorescent vitamin D binding protein (merge). The zebrafish eye is enlarged to show the retinal plexus. (c) Tg(l-fabp:VDBP:eGFP) transgenic zebrafish can be used to indirectly monitor proteinuria. Loss of green fluorescent protein in FSGS serum injected fish leads to reduced GFP signal in the retinal vessels where it can easily be quantified. Quantification of loss of fluorescent vitamin D binding proteins was done by measuring maximum GFP fluorescence in the retinal vessel plexus of Tg(l-fabp:VDBP:eGFP) zebrafish larvae at 120 hpf. Zebrafish larvae were injected with serum: dextran mixture from a healthy control and from a patient with FSGS recurrence in the kidney transplant at 48 hpf. *p < 0.05. n = 107. Scale bar = 500 µm. (d) Cryo sections of Tg(l-fabp:VDBP:eGFP) transgenic zebrafish larvae at 120 hpf showing systemic decrease in VDBP:eGFP in the systemic vascular system in FSGS serum injected zebrafish as a hint for proteinuria. Zebrafish were injected with serum from CTRL or FSGS patient at the time of disease recurrence. Scale bar = 100 µm. (e) Electron microscopy picture of the glomerular filtration barrier of 5 day old zebrafish larvae that were injected with either serum of the patient from the time of FSGS recurrence or with CTRL serum at 48 hpf. Black arrow head shows podocyte effacement. Scale bar = 500 nm. hpf: hours post fertilization, VDBP: Vitamin D binding protein.

Type A actin arrangement with more than 90% of cell area filled with thick cables was significantly less abundant whereas type B and C with no thick cables and no cables in the central area of the cell were significantly more often after podocytes were exposed to serum derived from our patient with recurrent FSGS. In contrast, serum from a nephrotic patient with MGN did not induce cytoskeletal changes (Fig. 2Aa,b). Thus, the in vitro assay indicates the presence of unknown pathogenic circulating factors.

To test if these factors would also induce increased permeability of the glomerular filtration barrier, we used our zebrafish model for proteinuria screening. The zebrafish is an ideal model system for glomerular diseases as the zebrafish larvae develops a pronephros which is morphological almost indistinguishable from the human glomerulus and genes are highly conserved between zebrafish and human. In the past, we successfully used this zebrafish model to screen for proteinuria after genetic knockdown using morpholinos^17–24^. We usually use a transgenic zebrafish line that expresses a fluorescent Vitamin D binding plasma protein Tg(l-fabp:VDBP:eGFP fish) to indirectly measure proteinuria^25^. If plasma proteins are retained in the vascular system, the fluorescent signal from the Vitamin D binding protein can easily be detected in the retinal vessels of the zebrafish (Fig. 2Ba,c). In case of proteinuria, the fluorescent signal decreases^17–21,26^. Given the idea of an unknown circulating permeability factor, we refined our zebrafish model by injecting patient serum that was mixed 1:1 with red fluorescent dextran to control proper injection procedure in the zebrafish circulation at 48 h post fertilization (hpf) (Fig. 2Ba,b). Compared to zebrafish that were injected with serum from a healthy control, zebrafish injected with serum from the patient with recurrent FSGS developed lost high molecular weight plasma proteins at 120 hpf (Fig. 2Bc,d). In contrast, FSGS serum at the time of remission did not cause proteinuria (supplementary Fig. S1). We performed transmission electron microscopy of the zebrafish pronephros of fish that were injected with the serum of the FSGS patient from the time of disease recurrence and could show podocyte effacement compared to CTRL serum injected zebrafish (Fig. 2Be).

Taken together these tests proved that our patients FSGS serum contains a so far unknown permeability factor that acts on the podocyte cytoskeleton and changes the permeability of the glomerular filtration barrier.

### Raman spectroscopy reveals molecular fingerprint of FSGS serum and changes in podocyte metabolome induced by FSGS serum

**Figure 3:**
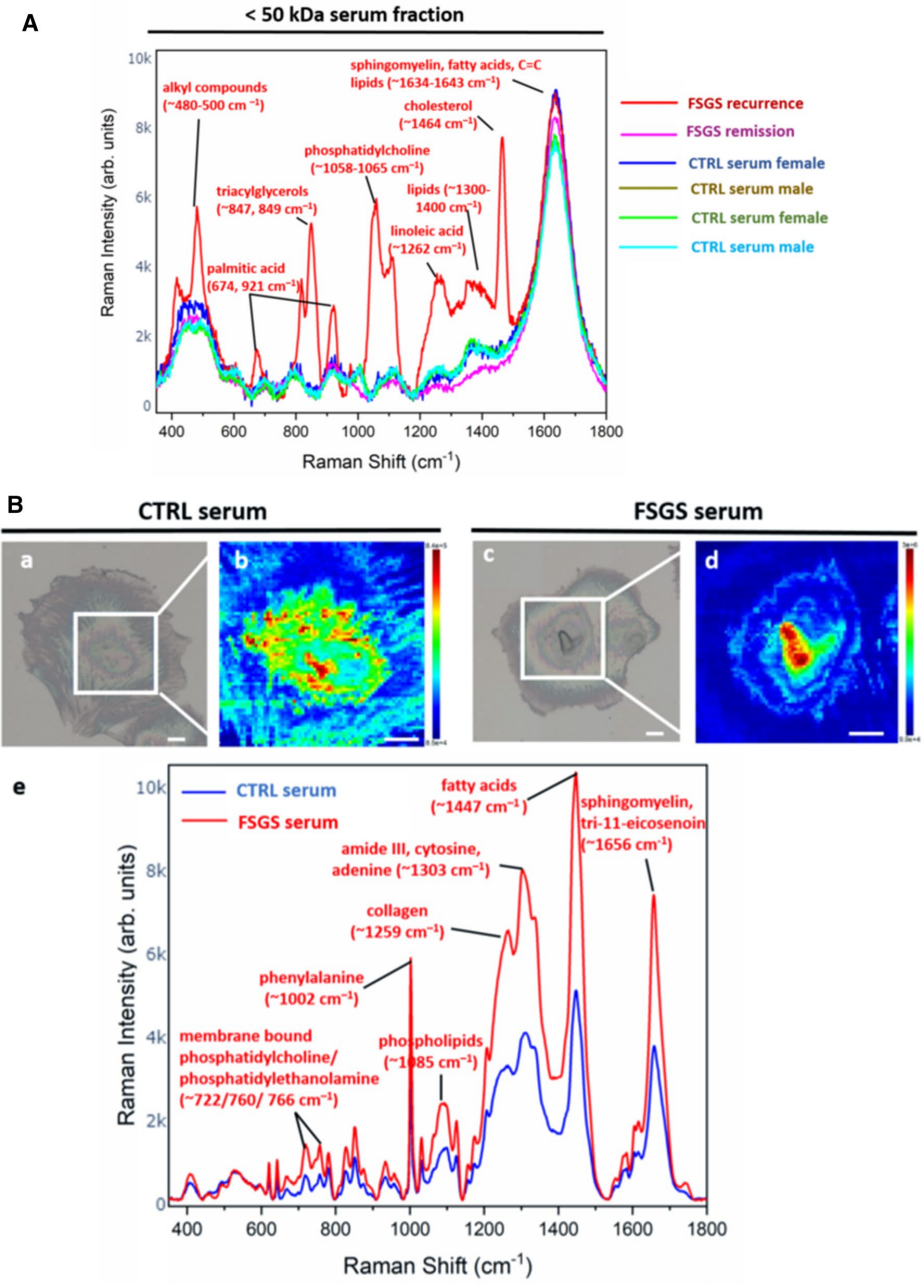
Raman spectroscopy reveals molecular fingerprint of FSGS serum and changes in podocyte metabolome induced by FSGS serum. (**A**) Mean Raman spectra of < 50 kDa serum fractions of FSGS at the time of disease recurrence (red), at the time of remission (purple) as well as of < 50 kDa serum fractions of four healthy control person (dark blue, green, brown and light blue). Different Raman signal corresponding mostly to lipoproteins were detected at the time of FSGS recurrence. Raman signals at the time of FSGS remission however resembled Raman signals of the CTRL serum fraction. Assignments of the Raman peaks according to the literature are given. (**B**) Representative bright field illumination (a,c) and heat map of Raman signal intensity (b,d) of cultured human podocytes treated with CTRL serum (a,b) and FSGS serum (c,d). Scale bar 10 µm. (e) Mean Raman spectra of three podocytes treated with FSGS serum (red line) and three podocytes treated with CTRL serum (blue line) showing increased Raman signal for FSGS treated cells.

In order to further characterize this unknown factor, we used a novel technique, which has so far not been used in the context of FSGS, Raman spectroscopy. This spectroscopy method is a technique for optical characterization of the compositional properties of materials. It detects the inelastic scattering of light from molecules, which results in a change in wavelength that corresponds to specific molecular vibrational modes. As CytoSorb therapy eliminates molecules with a molecular weight below 50 kDa, we analyzed the < 50 kDa serum fractions of the FSGS patient before the first CytoSorb apheresis at the time of FSGS recurrence and at the time of remission as well as in four healthy control persons by Raman spectroscopy. Raman shifts corresponding to alkyl components (~ 500 cm^–1^), phosphatidylcholine (~ 876 cm^–1^, ~ 1065 cm^–1^), lipid fractions (~ 1300–1400 cm^–1^) and cholesterol (~ 1442 cm^–1^) were elevated at the time of FSGS recurrence. In contrast, Raman signals of the < 50 kDa serum fraction at time of FSGS remission resembled the Raman signal of < 50 kDa serum fraction of control persons (Table 1 and Fig. 3A). Thus, Raman spectroscopy revealed a changed serum lipid metabolome in FSGS. Raw data of Raman intensity and Raman shift can be found in supplementary table S1.

**Table 1:** Raman spectra wavelength with corresponding published assignments .

Raman shift	Assignment	References^a^
~ 417 cm^–1^	Cholesterol	^77,78^
~ 480–500 cm^–1^	Alkyl components	^85^
~ 674/912 cm^–1^	Palmitic acid	^86^
~ 720/780 cm^–1^	DNA/RNA	^79,80^
~ 722/718, 766 cm^–1^	Membrane bound/free phosphatidylcholine	^81,86^
~ 743 cm^–1^	Thymine	^77,79,80^
~ 760 cm^–1^	Phosphatidylethanolamine	^82^
~ 847/849 cm^–1^	Triacylglycerols	^86^
~ 876/1058–1065 cm^–1^	Phosphatidylcholine	^82,86^
~ 1003/1030 cm^–1^	Phenylalanine	^77,79,80^
~ 1002/987 cm^–1^	Membrane bound/free phosphatidylcholine	^81^
~ 1068 cm^–1^	Cholesteryl palmitate	^86^
~ 1125 cm^–1^	Carbohydrates	^70^
~ 1125/1580 cm^–1^	Cytochrome c	^77,79,80^
~ 1131 cm^–1^	Cholesterol/cholesteryl palmitate	^86^
~ 1259 cm^–1^	Collagen	^79^
~ 1262 cm^–1^	Linoleic acid	^86^
~ 1303 cm^–1^	Amide III, cytosine, adenine	^83^
~ 1433/1457 cm^–1^	α(CH2/CH3) and β(CH2/CH3) of fatty acid	^77,79,80,84^
~ 1442/1464 cm^–1^	Cholesterol	^86^
~ 1643 cm^–1^	Sphingomyelin	^56^
~ 1656 cm^−1^	tri-11-eicosenoin	^86^
~ 1660 cm^–1^	Amide I	^77,79^
~ 1665 cm^–1^	CC lipids	^77,79^

^a^Slight differences in Raman shifts between measurements in this manuscript and references from the literature can be due to differences in Raman laser energy and molecular composition.

To explore the molecular basis of the patient’s serum-induced morphological changes on podocytes further we performed Raman micro-spectroscopy mapping of cultured podocytes treated with FSGS serum and control serum. Interestingly, the Raman signal intensity of cells treated with control serum for 6 h was less intense compared to podocytes treated with FSGS serum (Fig. 3B). Among others, increased Raman signal shifts assigned to membrane bound phosphatidylcholine/phosphatidylethanolamine (~ 722, 760, 766 cm^–1^), to phenylalanine (~ 1003 cm^–1^), phospholipids (~ 1085 cm^–1^), fatty acids (~ 1447 cm^–1^ ) and to sphingolipid cluster (~ 1656 cm^–1^) were detectable in podocytes treated with FSGS serum (Table 1). Raman signal corresponding to collagen showed a vertical but also horizontal shift indicating collagen modification due to FSGS serum treatment. Raw data of Raman signal intensity and Raman signal shift can be found in supplementary Table S2.

Taken together, changes in Raman signals in FSGS treated cultured human podocytes could mostly be attributed to cellular lipoproteins.

### Raman spectroscopy gives a molecular fingerprint of recurrent FSGS on tissue level

**Figure 4:**
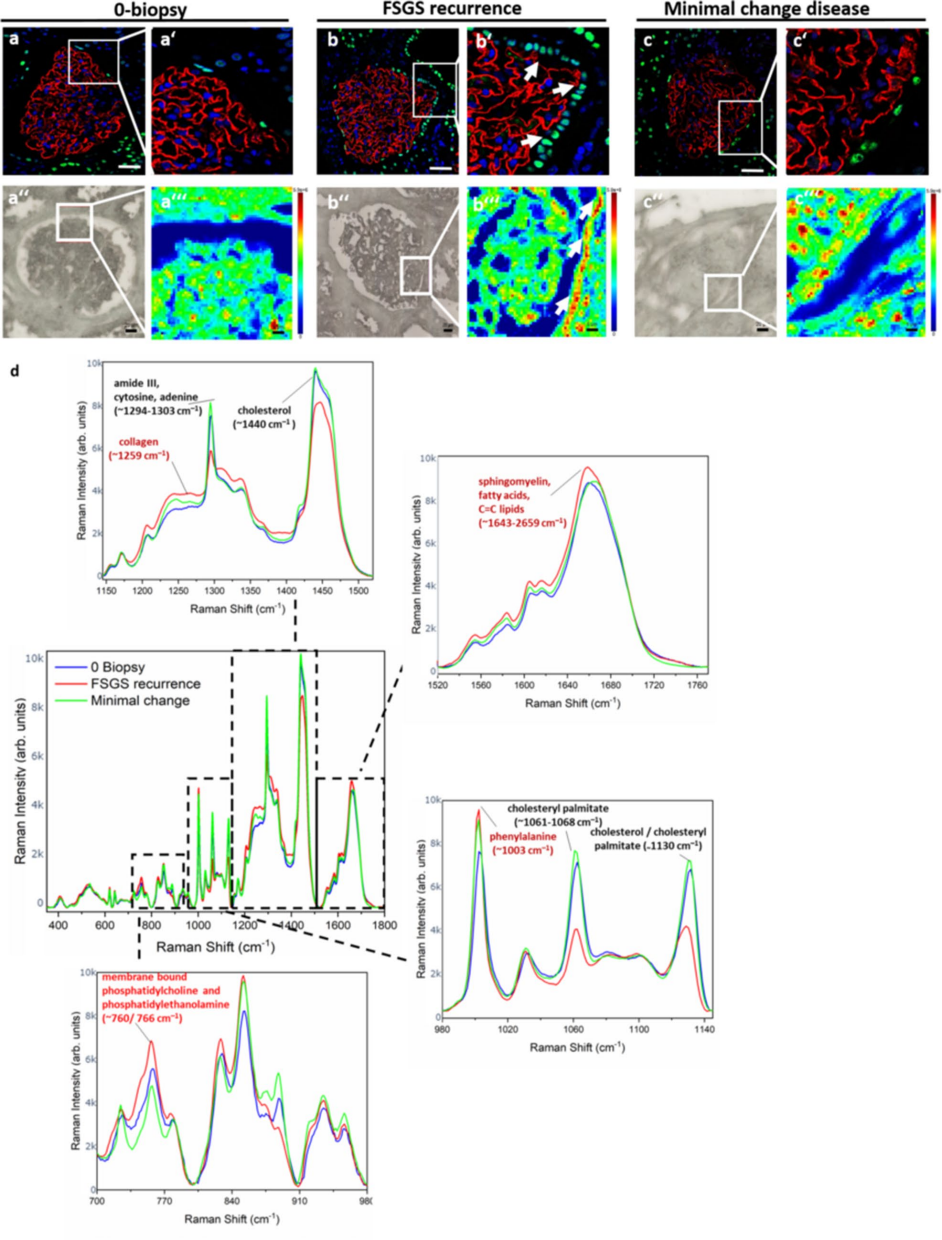
Raman spectroscopy gives a molecular fingerprint of recurrent FSGS on tissue level. (**a**,**b**) PAX8 staining (green) and synaptopodin staining (red) of a glomerulus from the kidney biopsy at transplantation (0-biopsy) (**a**,**a′**) and at the time of FSGS recurrence (FSGS recurrence) (**b**,**b′**) and from a patient with minimal change disease (**c**,**c′**) showing increased PAX8 staining in the Bowman’s capsule (arrow head in **b′**), scale bar = 100 µm. Representative bright field illumination (**a″**,**b″**,**c″**) and heat map of Raman signal intensity (**a‴**,**b‴**,**c‴**) of a glomerulus from the kidney biopsy at transplantation (0-biopsy) (**a″**,**a‴**), at the time of FSGS recurrence (FSGS recurrence) (**b″**,**b‴**) and of a biopsy with minimal change disease (**c″**,**c‴**) showing increased Raman signal at the region of parietal epithelial cell in the Bowman’s capsule (arrow head in **b‴**). Scale bar = 50 µm. (**d**) Mean Raman spectra of three glomeruli from the kidney biopsy at transplantation (blue line) and three glomeruli at the time of FSGS recurrence (red line). Assignments of the Raman peaks according to the literature are given.

To explore if we can also get a molecular fingerprint of recurrent FSGS on tissue level, we performed Raman micro-spectroscopy mapping on different glomerular regions of a kidney graft biopsy at time of transplantation (0 biopsy) (Fig. 4a'',a‴) and at the time of FSGS relapse (FSGS recurrence) (Fig. 4b'',b‴) and in a patient with minimal change disease (Fig. 4c'',c‴). Interestingly, increased Raman signal was detected in the region of parietal epithelial cells in the Bowman’s capsule in the biopsy with FSGS recurrence indicating activation of these cells (arrowheads in Fig. 4b′,b‴). We could detect an increase in Raman peak abundance for membrane bound phosphatidylcholine and phosphatidylethanolamine, sphingomyelin, fatty acids and phenylalanine in the kidney biopsy with FSGS relapse. Especially peak ratio between sphingomyelin and cholesterol and between sphingomyelin and cholesteryl palmitate revealed the disturbance in lipids in kidney biopsy with FSGS relapse. Other lipids like membrane bound phosphatidylcholine and phosphatidylethanolamine showed a vertical as well as horizontal Raman shift that indicated differences in molecular composition as well as concentration change of these lipids. Most Raman shifts of the minimal changes biopsy were comparable to that of the 0-biopsy (Fig. 4d, Table 1).

**Figure 5:**
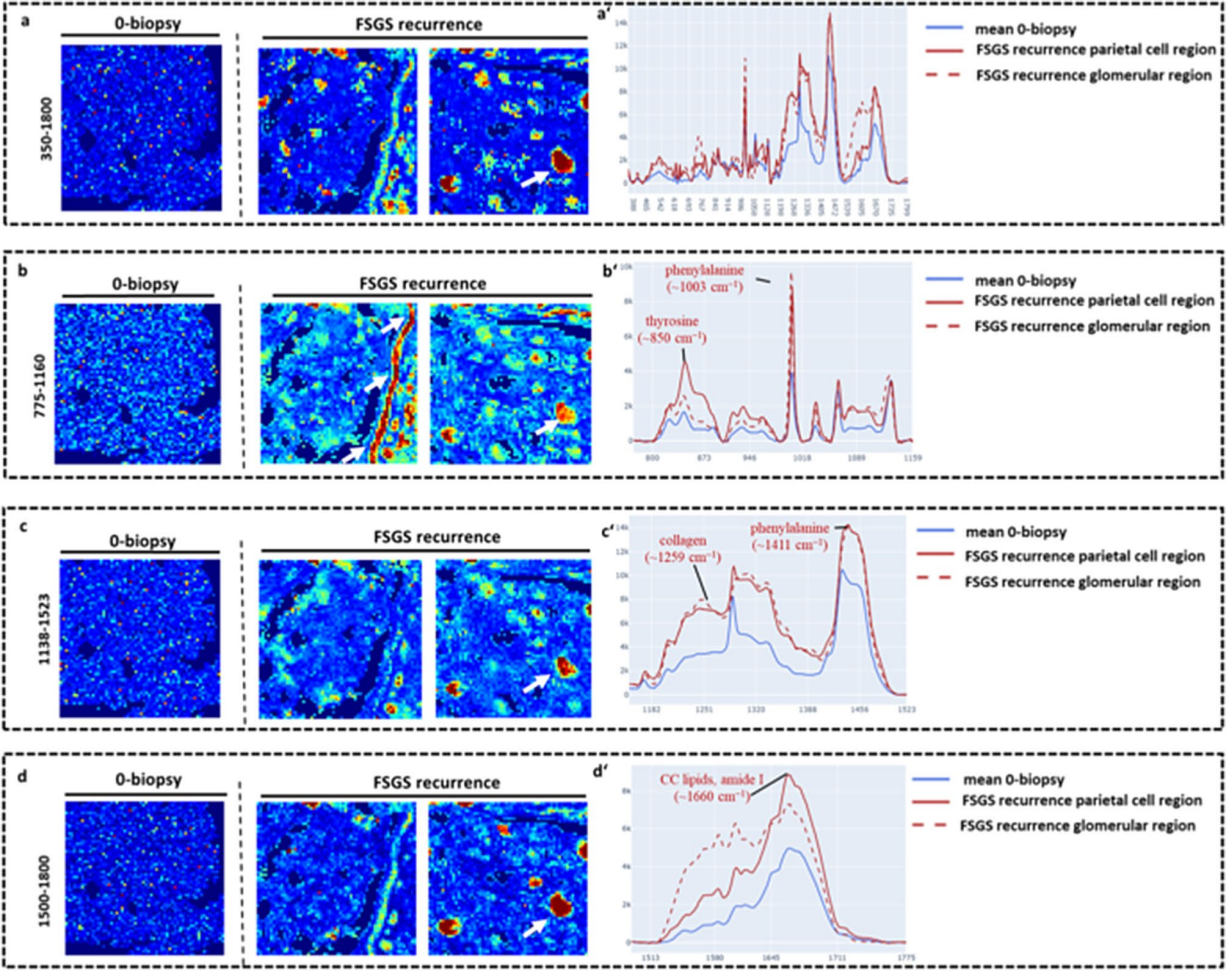
Machine learning reveals anomalies in Raman spectroscopy maps between 0-biopsy and FSGS recurrence. Raman spectra of glomeruli from the 0-biopsy and two glomeruli from the biopsy with FSGS recurrence were visualized, whereby the intensity and color range cover the degree of the anomaly. Areas of focal glomerular lesions as well as parietal epithelial cells in the Bowman capsule are highlighted as an anomaly in the FSGS samples. (**a**) Visualization of the occurring anomaly using the entire spectrum. (**b**–**d**) Consideration of only parts of the spectrogram that have been baseline corrected individually. Especially in the range of 775–1160 cm^−1^ (**b**) there were significant differences in FSGS glomeruli comparison to spectra from glomeruli of the 0-biopsy in focal areas as well in parietal cell region of the bowman capsule. Likewise, a similar effect was observed for the wavelength range 1138–1500 cm^−1^ (**c**) and 1500–1800 cm^−1^ (**d**). (**a′**–**d′**) Raman spectra for the anomalies with confidence > 80% in FSGS recurrence of the parietal cell region (red line) and the glomerular region (spotted red line) compared to 0-biopsy (blue line) for the entire spectrum (**a′**) and divided in wavelength range of 775–1160 cm^−1^ (**b′**) 1138–1523 cm^−1^ (**c′**) as well as 1500–1800 cm^−1^ (**d′**).

To analyze anomalies in Raman spectra between 0-biopsy and FSGS recurrence we generated a Raman spectroscopy map from two measurements belonging to each biopsy and used a machine learning approach (One-Class SVM) (Fig. 5). Raman spectra were visualized, whereby the intensity and color range cover the degree of the Raman anomaly. Areas of parietal epithelial cells in the Bowman capsule as well as focal glomerular lesions (white arrows in Fig. 5) could be detected as an anomaly in the FSGS samples. Dividing the whole Raman signal into only parts of the spectrogram revealed that there were significant differences in focal areas of glomeruli in the FSGS recurrence biopsy (dotted red line in Fig. 5a′–d′) compared to the 0-biopsy (blue line in Fig. 5a′–d′). This was most prominent in the wavelength range of 775–1160 cm^−1^, 1138–1523 cm^−1^ as well as 1500–1800 cm^−1^. Anomaly spectra from the parietal cell region of the bowman capsule were most prominent in wavelength range of 775–1160 cm^−1^ (red line in Fig. 5a′–d′). Raw data of Raman intensity and Raman shift can be found in supplementary Table S3.

### Serum metabolome analysis of FSGS serum reveals potential disease-causing factors

**Figure 6:**
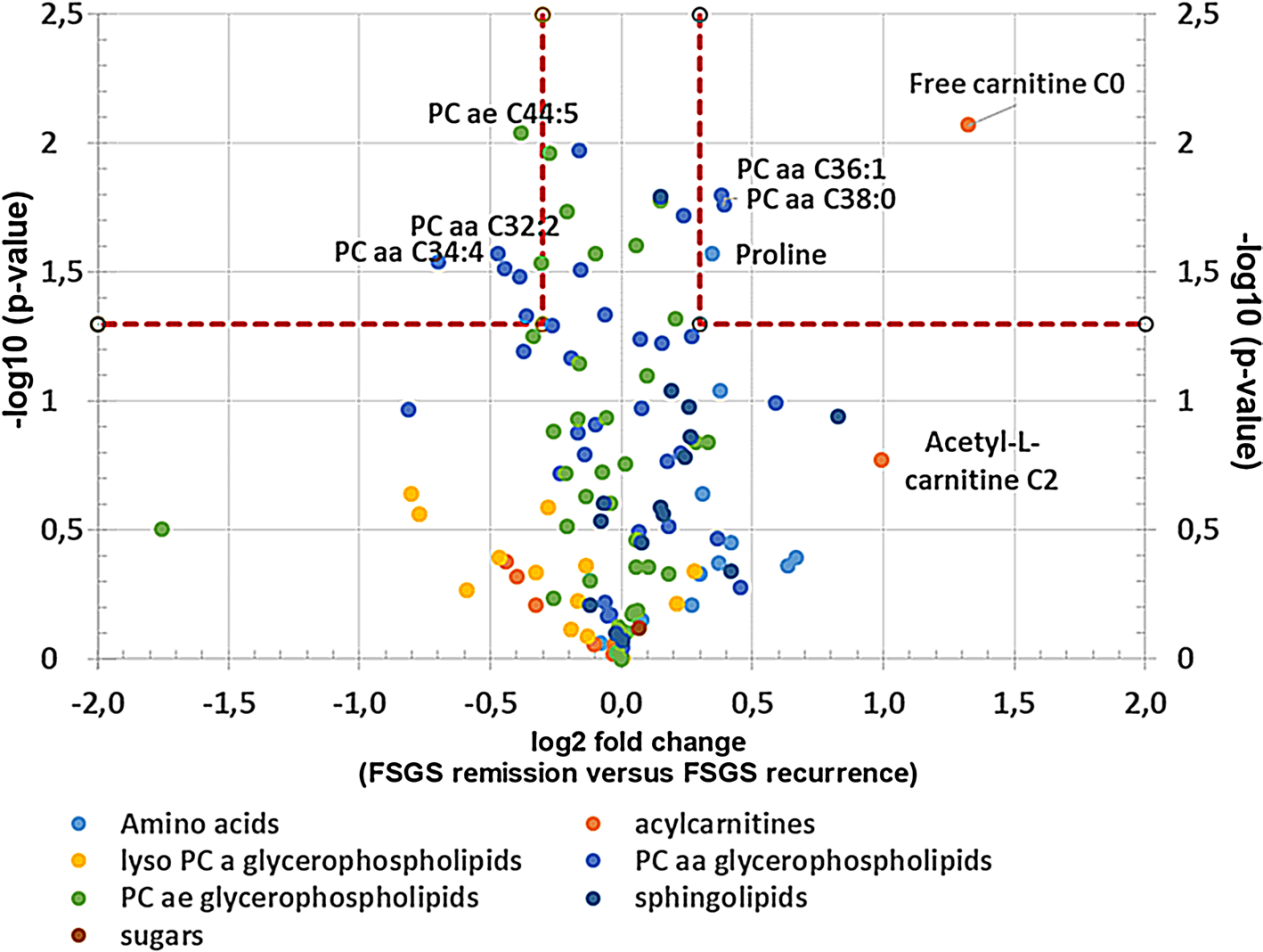
Serum metabolome analysis of recurrent FSGS reveals changes in carnitine and phosphatidylcholine-levels. Volcano-Plot of the serum metabolome analysis of the patient with FSGS. Log2 fold change of metabolites at FSGS remission versus FSGS recurrence as well as –log10 (p-values) are given. PCaaC34:4 and l-carnitine were significantly altered at remission versus recurrence. Confidence limits (fold change > 0.3, p-value < 0.05) are shown with dashed lines. Metabolomics are labeled in different colors according to their metabolomics class.

To characterize the patient’s serum further we performed mass spectrometry analysis. 163 metabolites and metabolite ratios were analyzed by a targeted mass spectrometry approach in the FSGS patient’s serum at different time points as well as in serum from a stable transplanted control patient. This patient had stable transplant function (serum creatinine around 80 µmol/l), no proteinuria and no rejection. Transplantation time and baseline immunosuppression was the same as in our FSGS recurrent patient. Ornithine/arginine ratio, ornithine, different lysophosphatidylcholines, tyrosine phosphatidylcholines as well as proline were increased in the FSGS serum at the time of relapse in the kidney transplant compared to serum of a stable transplanted patient (log2 fold change > 0.3) (Table 2). In contrast, different acylcarnitines (C0, C2, C3-DC (C4-OH)) and tyrosine/phenylalanine ratio were decreased in serum of the FSGS patient compared to serum of the control patient (log2 fold change < − 0.5 (Table 2). Comparing the metabolite abundances at the time of FSGS remission to that at the time of recurrence revealed a decrease of initially elevated lysophosphatidylcholines and an increase in initially reduced acylcarnitines (Table 3). This change was significant for l-carnitine C0 (log2 fold change =  + 1.33; − log10 (p-value) = 0.009) and phosphatidylcholine aaC34:4 (log2 fold change of − 0.75, − log10 (p-value) = 0.029) (Fig. 6). Comparing different metabolite classes after the first CytoSorb apheresis to that before the first CytoSorb apheresis showed a decrease in amino acids initially increases in FSGS serum and an increase in acylcarnitine initially reduced (Table 4)*.* CytoSorb apheresis in a stable phase of FSGS further decreased lysophosphatidylcholines and phosphatidylcholines that were still elevated compared to the control patient (Table 5). Raw data of mass spectrometry analysis normalized by the serum of the stable transplanted patient can be found in supplementary Table S4. The altered lipidome/metabolome in our FSGS patient with increase in phosphatidylcholines and ornithine and decrease in acylcarnitines could be confirmed when compared to ten 10 healthy control persons from previously published EPIC-study (supplementary Table S5)^27^.

**Table 2:**
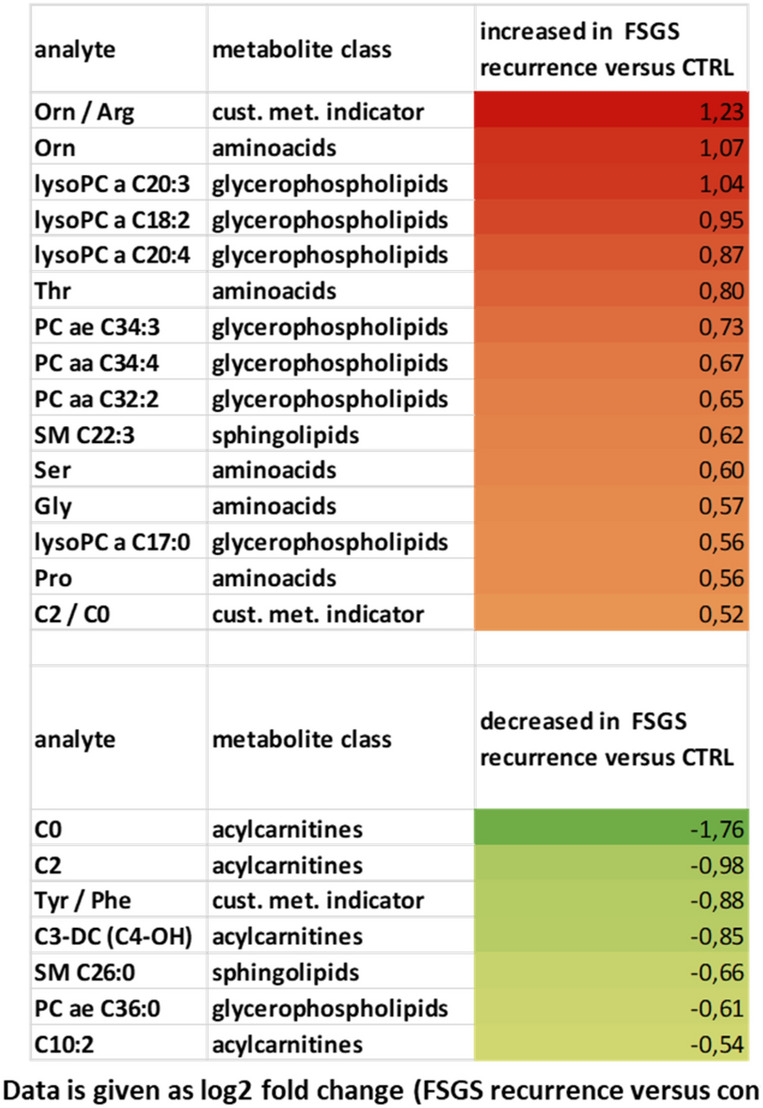
Differences in serum metabolites of a transplanted patient with FSGS recurrence versus a transplanted control measured by mass spectrometry.

Table shows metabolites that were increased > 0.5 and decreased > 0.5 in the FSGS patient competed to the control patient. All values are given as log2 (FSGS patient/stable transplanted control).

Color shades represent values as heat map.

**Table 3:**
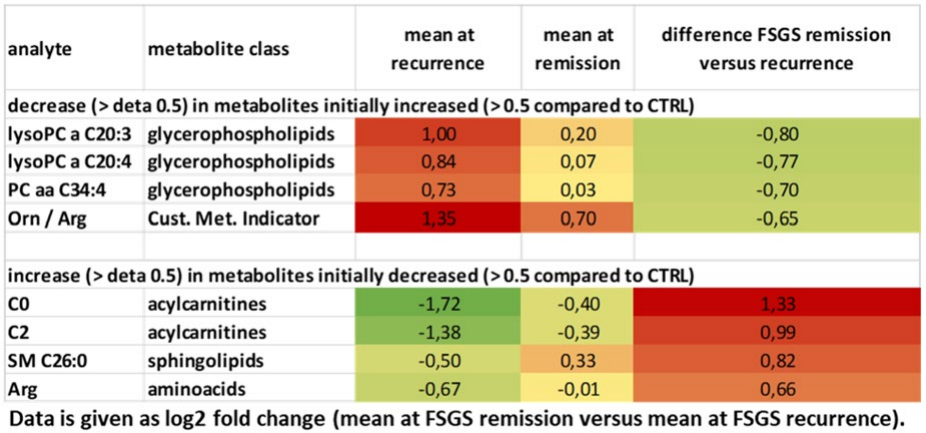
Difference in serum metabolites in FSGS remission versus recurrence measured by mass spectrometry.

Table shows metabolites that were initially increased > 0.5 and decreased more than > 0.5 as well as metabolites that were initially decreased and increased more than > 0.5. All values are given as log2 (FSGS patient/stable transplanted control). The mean of two measurements at the time of FSGS relapse (before and after the 1. CytoSorb apheresis) to the mean of two measurements at the time of FSGS remission (before and after the CytoSorb apheresis at stable remission) were used for calculations.

**Table 4:**
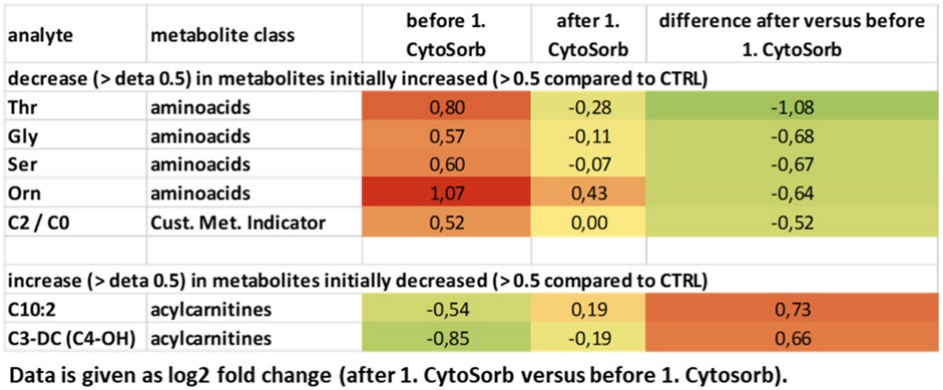
Difference in metabolites after first CytoSorb versus before first CytoSorb measured by mass spectrometry.

Tables shows metabolites that were initially increased > 0.5 and decreased more than > 0.5 as well as metabolites that were initially decreased and increased more than > 0.5. All values are given as Log2 (FSGS patient/stable transplanted control).

**Table 5:**
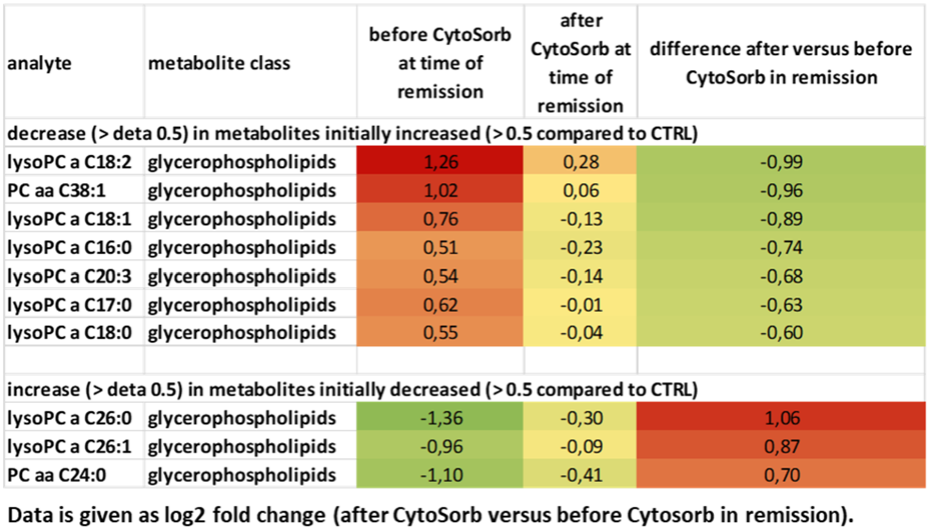
Difference in metabolites after versus before CytoSorb in remission measured by mass spectrometry.

Table shows metabolites that were initially increased > 0.5 and decreased more than > 0.5 as well as metabolites that were initially decreased and increased more than > 0.5. All values are given as Log2 (FSGS patient/stable transplanted control).

Color shades represent values as heat map.

In summary, mass spectrometry analysis revealed changes in serum lipoprotein profile in FSGS that could be influenced by CytoSorb therapy. Furthermore, mass spectrometry results correlated to Raman signals found in FSGS serum treated podocytes, FSGS serum fractions and FSGS kidney biopsy.

## Discussion

Idiopathic FSGS is a disease group that is believed to be caused by circulating permeability factors. Despite long lasting research efforts over many decades and several identified potential factors a unifying concept has not been established for circulating factors. Here, we report a patient with recurrent idiopathic FSGS negative for all published circulating permeability factors. Since we previously published successful removal of suPAR treatment with CytoSorb apheresis in a patient with FSGS and since our patient had progressed to end-stage renal disease of her native kidney despite all other treatment attempts we decided to use CytoSorb as compassionate use^28^. CytoSorb preferentially absorbs hydrophobic substances and remove molecules with a weight range between 5 and 60 kDa. Already one treatment with CytoSorb apheresis led to rapid decrease in proteinuria with no side effects in this patient. However, at the beginning of the treatment proteinuria relapsed shortly after apheresis frequency was reduced. This might indicate the rapid rebound of the circulating components after CytoSorb treatment. Only after daily treatment sessions over several weeks and additional rituximab treatment CytoSorb apheresis sessions could be reduced to once a week. Even though the patient achieved remission with our treatment, the actual circulating component in the patient’s blood remains unknown.

There is plenty of evidence for a causative circulating factor in idiopathic FSGS: serum from patients with FSGS increases glomerular albumin permeability in vitro and induces proteinuria in rats^7,29^. Proteinuria can recur days to weeks after kidney transplantation and plasmapheresis is able to induce proteinuria remission^30,31^. Implantation of a kidney allograft with FSGS in another patient was successful without causing proteinuria^32^. One of the most prominent, but also most debated circulating factor suggested to cause FSGS is suPAR^8,33^. suPAR is released during inflammation or immune activation, and therefore the suPAR levels reflect immune activation^34^. Normal suPAR level range from 2000–3000 pg/mL in healthy individuals, about 3000–4000 pg/mL in unselected patients in emergency departments, and about 9000–10,000 pg/mL in critically ill patients. suPAR levels are higher in females compared to males and smoking is associated with an increase in suPAR^35^. We could show in a previously published case report, that CytoSorb treatments can decrease suPAR levels^28^. However, our recent patient showed suPAR levels in a normal range. CLCF1 is another putative circulating permeability factor described in FSGS^36^. Recombinant human CLCF1 increased albumin permeability of isolated rat glomeruli^37^. Incubation of cultured murine podocytes with CLCF1 caused marked changes in the configuration of the actin cytoskeleton^38^. Serum CLCF1 concentration was 1.67 ng/ml at the time of FSGS recurrence in our patient. Even though undetectable in most of our healthy controls, serum CLCF1 reached concentrations up to 1.2 ng/ml in one control. Therefore, we do not believe that CLCF1 is specific for recurrent FSGS and most likely was not the disease-causing factor in our patient. Kemper et al. observed increased levels of the T-cell activation marker sCD25 during relapses of steroid dependent nephrotic syndrome^39^. sCD25 level was in normal range before the first apheresis session in our patient. Cathepsins are other suggested candidates for the circulating permeability factor. Cathepsins are proteases involved in intracellular protein degradation and activation of enzyme precursors. Immunohistochemically staining of human kidney biopsy specimens indicated that the expression of cathepsin D was significantly increased in Minimal change disease compared to that in FSGS maybe because of a high level of autophagic activity^40,41^. IL6 was demonstrated to contribute to renal diseases like FSGS^42^. Cathepsin L and IL6 were undetectable or in normal range in our patient.

Since our patient had normal range of published circulating permeability factors, we used our cell culture model and our zebrafish proteinuria assay to show that the patient’s serum contained disease-causing factors. Cultured human podocytes can be a useful bioassay to monitor disease activity and to screen for podocyte damaging factors. Sera of patients with recurrent FSGS induced downregulation of SMPDL-3b in cultured podocytes making them more susceptible to actin remodeling^43^. We treated cultured human podocytes with the patient’s serum and could detect a significant cytoskeleton rearrangement. Actin stress fibers in the central part of the cells decreased and the cells displayed a typical actin rim-like structure. These changes are consistent with activation of the cell towards a more motile phenotype that may be more vulnerable to detachment.

In the past, we established the zebrafish as a screening model for proteinuria in gene knockdown models^17–24^. Now, we refined our model for a screening of circulating permeability factors. We injected serum of patient with recurrent FSGS in the cardinal vein of the zebrafish and detected a significant loss of plasma proteins and partial podocyte effacement 3 days later.

In order to characterize the unknown circulating permeability factor further, we performed Raman spectroscopy in < 50 kDa serum fractions, on kidney biopsies and on podocytes treated with serum of our patient with recurrent FSGS. Raman spectra are directly related to the biochemical composition of tissues^44–46^. In the past, Raman was used to detect metabolomic changes in different cancers^47,48^. However, Raman spectroscopy was never used before to study metabolomics in FSGS. Li et al. demonstrated that Raman spectroscopy combined with multivariate analysis can be a potential non-invasive diagnostic tool for nephritis in an anti-GBM mouse model^44^. We previously used Raman to detect cell stress induced by micro particles^49^. As the disease-causing factor was unknown in our patient, we used a global approach based on the ability of Raman to identify spectral markers of the global intrinsic molecular composition. Most prominent differences in Raman peaks between FSGS serum treated and control serum treated cultured human podocytes were found at wavelength corresponding to membrane bound phosphatidylcholine, phenylalanine, phospholipids, fatty acids and sphingomyelin.

Small vertical wavelength shifts were present between FSGS serum and control serum treated human podocytes between 700 and 800 cm^−1^. It has been suggested that differences in protein secondary structures result in a horizontal shift of Raman bands^50,51^. For example, the phenylalanine bands shifted between 997 and 1007 cm^−1^ in different types of collagen. Raman spectra of < 50 kDa serum fraction of the FSGS patient at the time of recurrence corresponded to phospholipids, phosphatidylcholine, and sphingomyelin confirming a dysbalance in the serum lipoprotein profile.

On tissue level, increased Raman signal was detected in the region of the Bowman’s capsule in the biopsy with FSGS recurrence but not in the preimplantation biopsy. We have also applied a machine learning based anomaly detection to identify discrepancies in the Raman spectra between the 0-biopsy and FSGS recurrent biopsy. Differences were found in the area of parietal epithelial cells and in the focal points of the glomerulus in the FSGS recurrent biopsy. Recently, a novel parietal epithelial cell subpopulation called cuboidal parietal cells co-localized with the Bowman’s capsule were proposed to form tip lesions in FSGS^52^. In line, activation of parietal epithelial cells has been described in early recurrent FSGS^53^. In three different models of FSGS and in human biopsies with FSGS focal activation of parietal epithelial cells contributed to the development and progression of sclerotic lesions^54^. Thus, the increased Raman signal in the Bowman’s capsule in recurrent FSGS might correspond to activated parietal epithelial cells.

Raman signal of the FSGS biopsy again revealed increased membrane bound phosphatidylcholine, phenylalanine, phospholipids, fatty acids and sphingomyelin. Increased Raman peaks in the FSGS biopsy corresponding to phosphatidylcholine, phospholipids and fatty acids were in line with disturbed systemic and renal lipid expression in FSGS^55^. A characteristic Raman band of sphingomyelin was identified at ∼1643 cm^−1^^56^. This Raman signal was increased in our FSGS biopsy compared to the preimplantation-biopsy. Increased collagen along the Bowman’s capsule was reported in FSGS mice and fitting to our Raman measurements with increased signal 1259 cm^−1^^57^. Albumin has major Raman peaks at 830 cm^−1^, 950 cm^−1^, 1350 cm^−1^, and 1650 cm^−1^^58^. All these spectra were increased in FSGS relapse biopsy compared to 0-biopsy indicating a higher albumin abundance in the damaged kidney due to leakage in the glomerular filtration barrier.

Dyslipidaemia is a typical finding of nephrotic syndrome including FSGS. The beauty of our study is that for the first time we could not only detect this dyslipidaemia in serum but also disturbed lipid profiles on tissue level and in podocytes treated with FSGS serum using Raman spectroscopy. These changes in lipoproteins might reveal novel pathways involved in the pathomechanism of recurrent FSGS.

In addition to Raman spectroscopy, we performed mass spectrometry in the patient’s serum at different time points of the disease to characterize the circulating metabolome in FSGS. Metabolite signatures have been demonstrated to possess diagnostic or predictive power for several renal dysfunctions such as acute kidney injury, chronic kidney disease, diabetic nephropathy, kidney cancer, membranous nephropathy, polycystic kidney disease as well as for transplant rejection^59^. Fouque et al. could show that several acyl-carnitines were significantly increased and inversely associated with lower eGFR^60^. Plasma free carnitine concentrations were significantly higher in the acute period of steroid-sensitive nephrotic syndrome compared to the remission period and plasma free carnitine positively correlated with low-density lipoprotein cholesterol, total cholesterol and triglyceride^61^.

Phosphatidylcholine, lysophosphatidylcholine, and sphingomyelin were all described to be elevated in diabetic nephropathy and dysregulation of ceramide metabolism was recently reported to be also involved in diabetic kidney disease^62,63^. Metabolomic profiling of patients with a failing kidney allograft revealed a correlation of serum concentrations of tryptophan, glutamine, dimethylarginine isomers and short-chain acyl-carnitines (C4 and C12) with a reduced GFR^64^. There is emerging evidence that disturbed lipid metabolism might play a role in FSGS. Erkan et al. reported increased fatty acids and phosphatidylcholines as well as reduced phosphatidylcholines in urines from patients with FSGS^65^.

In our analysis, we compared mass spectrometry data of the patient’s serum at time of FSGS recurrence to serum of a transplanted control and the FSGS serum before and after CytoSorb treatment at the time of FSGS relapse and at the time of remission. Even though a targeted mass spectrometric approach can only cover a predefined set of metabolites the accuracy and reproducibility is higher compared to profiling approaches and was therefore used in this study. Several molecules of the changed lipid metabolome profiles associated with idiopathic FSGS interact with each other and were published in similar context: Phosphatidylcholine and lysophsphatidylcholine were both elevated at the time of FSGS relapse. Both lipids are converted to each other by lecithin–cholesterol acyltransferase (LCAT) and lysophosphatidylcholine acyltransferase (LPCAT). Well in line, lysophosphatidylcholine 16:0 and 18:0 were also found in a podocyte-selective injury mouse model^66^. Furthermore, podocyte injury-driven lysophosphatidylcholine accelerated glomerular macrophage-derived foam cell infiltration via lysophosphatidylcholine-mediated expression of adhesion molecules and chemokines in glomerular resident cells in FSGS^66^. In addition, phosphatidylcholines were accumulated in the FSGS serum. Urine of patients with FSGS was previously described to contain elevated levels of fatty acids (C16:0, C22:4) and lysophosphotidylcholines (C14:0, C18:1) but decreased levels of phosphotidylcholine (C38:4) compared to healthy subjects^65^.

Serum sphingomyelin was reduced in our FSGS patient in our mass spectrometry analysis. In contrast, Raman spectroscopy revealed increased signal corresponding to sphingomyelin on podocyte and tissue level in FSGS. Dysregulation and tissue accumulation of different sphingolipids are typical findings in genetic diseases including Tay–Sachs disease, Fabry disease, hereditary inclusion body myopathy 2, Niemann–Pick disease, and nephrotic syndrome of the Finnish type^67–70^. Similarly, sphingolipid accumulation has also been reported in glomerular diseases of non-genetic origin including diabetic kidney disease, HIV-associated nephropathy, lupus nephritis and idiopathic FSGS^70,71^. Sphingomyelins are synthesized during the transfer of phosphorylcholine from phosphatidylcholine to ceramide in a reaction catalyzed by sphingomyelin synthase. SMPDL3b, an enzyme that modulates sphingomyelinase activity in podocytes has been shown to be reduced in FSGS^43,72^. Thus, our findings are well in line with previously described dysregulation of sphingolipids in FSGS.

Sphingomyelin synthesis from ceramide and phosphatidylcholine is catalyzed by sphingomyelin synthase. We speculate that the decrease in sphingomyelin in the patient at the time of FSGS recurrence most likely was due to decreased ceramide and decreased sphingomyelin synthase activity and at the same time causing an increase in phosphatidyl-choline due to accumulation. A decrease in catabolism of amino acids might have resulted in decreased amino-acid derived acylcarnitines and higher levels of tyrosine seen in the patient.

Moreover, acylcarnitine that also belongs to the sphingolipid family was reduced at the time of FSGS relapse. Acylcarnitines were previously described to be reduced in urines from FSGS patients^65^. Acylcarnitines play a role in fatty acid oxidation and transport of acyl-CoA across the inner mitochondrial membrane^73^. A decrease in catabolism of amino acids might have resulted in decreased amino-acid derived acylcarnitines, l-carnitine and higher levels of tyrosine seen in the patient. Lower acylcarnitine levels are therefore a hint for impaired fatty acid oxidation and mitochondrial dysfunction. Taken together we identified several serum metabolomic signatures involved in lipid metabolism disturbances in FSGS that corresponded to Raman signals of FSGS serum, Raman signal of serum treated podocytes and Raman signals in the biopsy after FSGS recurrence. It is possible that not a single circulating factor but several molecules of the disturbed lipid metabolome might have induced the recurrence of FSGS and that the molecules we detected might have been surrogates rather than causal for the disease.

Our innovative methods might shed new light on the pathogenesis of recurrent FSGS and could be used as a novel tool to predict response to treatment. Metabolic profiling was shown to predict outcome of rituximab therapy in rheumatoid arthritis. Phenylalanine, choline, glycine, threonine and glycerol were all increased in non-rituximab responders versus rituximab responder^74^. Interestingly, all these metabolites were also increases in our FSGS patient that did previously not respond to rituximab when the disease occurred in the native kidneys (Tables 2 and 3). It is tempting to speculate that the CytoSorb therapy changed the metabolites and thus changed rituximab responsiveness after disease recurrence.

In summary, we provide novel evidence for additional circulating factors in FSGS causing early recurrence of the disease in the transplanted kidney. This is supported by the following pieces of evidence: First, the patient had normal levels of previously described circulating factors but rapidly responded to CytoSorb treatment. Second, the patient’s serum caused podocyte cytoskeleton rearrangements and proteinuria was induced by injection of the patient’s serum in zebrafish. Third, Raman spectroscopy was able to give a molecular fingerprint of recurrent FSGS on serum cell and tissue level and revealed metabolomic changes corresponding to serum mass spectrometry from the patient’s serum.

Our findings have several limitations. Assignments of Raman signals to lipid fractions was performed according to the literature and we did not measure the pure substances. However, we could detect dyslipidaemia in the FSGS serum by mass spectroscopy. Furthermore, our innovative methods were only performed in a very limited number of patient samples. However, we were the first analyzing Raman and mass spectroscopy serially over time in the same patient in serum and kidney biopsies. Next to the serial measurements in the same patient we used different controls like a stable transplanted patient, minimal change biopsy, serum from membranous glomerulonephritis and samples from healthy individuals as controls. As idiopathic FSGS is a heterogeneous disease most likely caused by different factors in different patients an individualized approach seems reasonable. Second, we did not actually identify “the” causing disease factor. However, we describe morphological and functional changes induced by the serum and found an altered lipid metabolome associated with idiopathic FSGS that might reflect a new subtype of FSGS. The innovative treatment management and analysis methods of this study might be used as a model for personalized treatment approaches and further research on recurrent FSGS. We believe that a patient centric approach is necessary to tailor treatment regimens for individual patients due to the heterogeneity of the disease.

## Methods

### CytoSorb apheresis

CytoSorb apheresis was performed in the department of Nephrology at University of Erlangen. The patient received daily/weekly CytoSorb apheresis over a cimino fistula on the left forearm. Blood flow rate was 200 ml/min. Anticoagulation during the apheresis was done with 1000 IE Heparin bolus followed by 1000 IE Heparin/h. Patients’ blood pressure, heart rate and electrolytes were measured during to the procedure. Venous pressure, arterial pressure and transmembrane pressure were controlled at the site of the apheresis machine. Informed consent was obtained from the participant.

### Measurement of circulating permeability factors

CLCF1 levels in patients’ serum were measured with human CLCF1 Sandwich ELISA Kit—LS-F7193 (Catalog** #** BMS257, LSBio, Seattle, WA, USA) according to the manufacture protocol. The ELISA can detect levels of CLCF1 between 0.156 and 10 ng/ml. According to the company, healthy subjects have serum levels between 0 and 1.5 ng/ml. Cathepsin L in patient’s serum was measured with human cathepsin L ELISA Kit (Catalog **#** BMS257, Invitrogen, Thermo Fisher scientific) according to the manufacture protocol. The ELISA can detect cathepsin L levels between 3.1 and 50 ng/mL. According to the company, healthy subjects have serum levels between 0 and 56 ng/ml. IL-6, suPAR and sCD25 were measured in the laboratory of the University of Heidelberg. The study was approved from ethic committee of Friedrich-Alexander University Erlangen-Nuremberg (182_19B) and the patient gave written consent. All experiments were performed in accordance with relevant named guidelines and regulations.

### Treatment of cultured human podocytes with patient serum

Immortalized cultured human podocytes were proliferated under permissive conditions at 33 °C. When cultivated at 37 °C, the SV40 T-antigen was inactivated for cell differentiation. Podocytes were differentiated for 10 days on cover slides in RPMI 1640 Medium (Roth, Karlsruhe, Germany) with 10% fetal calf serum, 1% Penicillin/Streptomycin and 0.1% Insulin. At day 7 fetal calf serum was replaced to 10% control serum or patient serum. Cells were fixed at 0 h and 6 h using ice-cold methanol at − 20 °C for 10 min and permeabelized using 0.1% Triton for 10 min. After blocking with 10% donkey serum, immunofluorescent labeling of F-actin was done by incubation with Alexa Fluor 546 phalloidin (Invitrogen) at 4 °C overnight. Nuclei staining was done with Hoechst. Sides were mounted on glass slides and were visualized under fluorescent microscopy. For quantification of changes in actin cytoskeleton of podocytes, we used a scoring system published earlier^14–16^: Type A: more than 90% of cell area filled with thick cables; type B: at least 2 thick cables running under nucleus and rest of cell area filled with fine cables; type C: no thick cables, but some cables present; type D: no cables visible in the central area of the cell.

### Serum injection in zebrafish larvae

Zebrafish were grown and mated at 28.5 °C. Larvae were kept and handled in standard embryo raising medium, as previously described. Two-day-old zebrafish larvae were anesthetized with 1.5% MS-222, transferred to an agarose injection mold. Zebrafish were injected with human serum derived from the patient and a healthy control into the cardinal venous sinus using a Drummond Nanoject 200 micro-injector. Following injection zebrafish were transferred to embryo raising medium to recovery. Larvae were checked daily until 120 h post fertilization at which point they were euthanized using 1.5% MS-222 or 1:500 2-phenoxy-ethanol.

We only used zebrafish until 120 hpf in our experiments, which does not need animal approval by licensing committees. Zebrafish breeding protocol were approved by the Office for Veterinary and Consumer Health Protection Erlangen (I/39/FN003). All methods were carried out in accordance with European Convention on the Protection of Vertebrate Animals (EU commission recommendation 2007/526/EC). The study was carried out in compliance with the ARRIVE guidelines.

### Proteinuria detection in zebrafish larvae

A transgenic zebrafish line that expresses a fluorescent Vitamin D binding protein Tg(l-fabp:VDBP:eGFP fish) was used to indirectly measure proteinuria. If plasma proteins are retained in the vascular system, the fluorescent signal from the Vitamin D binding protein increases over time and can easily be seen in the retinal vessels of the zebrafish. The maximum fluorescence intensities of grayscale images of the pupil of zebrafish larvae that were injected with control serum or patient serum at 48 hpf were measured using Image J (Version 1.48 Wayne Rasband National Institutes of Health, USA) and reported in relative units of brightness.

### Transmission electron microscopy of zebrafish larvae

Zebrafish larvae were fixed in solution D and embedded in EPON according to the manufacture protocol (recipe/protocol from EMS, Hatfield, PA). Semi-thin (300 nm) and ultra-thin (90 nm) sectioning were performed with a microtome (Reichert Austria Ultracut) and transferred onto copper slit grids (EMS, Hatfield, PA). Grids were optionally stained with uranyl acetate (2%) for 30 min and then lead citrate for 15 min with three washing steps in between. Images were recorded using Leo912 transmission electron microscope (Zeiss, Oberkochen, Germany).

For paraffin tissue sections, zebrafish larvae were fixed in 2% PFA for 1 day, dehydrated in ascending concentrations of ethanol in PBS (25%, 50%, 70%, and 100%) and transferred in xylene (100%) for 5 min before embedding in paraffin (60 °C) overnight. With a rotational microtome (Leica SM 2000 R) sections of 5 μm were cut, incubated in ethanol (100%, 70%, 50%, and 25%). For cryosections larvae were fixed in 2% PFA for 2 h followed by an overnight incubation in 30% saccharose at 4 °C. After embedding the larvae in Tissue-Tek (Sakura, Staufen, Germany), sections (20 μm, 60 μm) were cut using a Leica CM 1950 microtome and stained with antibodies. The following primary antibodies were used: anti-zebrafish nephrin (zNephrin, Innovagen, Sweden) 1:8000, Alexa Fluor 488 goat anti-rabbit (life technology) 1:500.

### Metabolome quantification

Concentrations of 163 metabolites including 13 amino acid/biogenic amines, sum hexoses, 41 acylcarnitines, 15 lysophosphatidylcholines, 77 phospho- and sphingolipids, and 15 sphingomyelines from serum samples were determined using a targeted metabolomics approach using the Absolute IDQ p150 Kit (Biocrates LIFE Science AG)^75^. Briefly, the liquid chromatography with tandem liquid chromatography with tandem mass spectrometry (LC‐MS/MS) analysis were carried out by means of multiple‐reaction monitoring (MRM) acquisition using a Waters Acquity UPLC System coupled with QTRAP 5500 (AB Sciex, Darmstadt, Germany). Raw data were analyzed in Analyst software 1.6.2 (Sciex, Framingham, MA, USA) and processed using MetIDQ software, which is an integrated part of the p150 Kit (Biocrates). Metabolites with log_2_ ratios above 0.3 or below − 0.3 are assumed to be up- or down regulated. Significance has been computed by two-sited paired Student’s *t*-test. Significance has been assumed in cases with p-values below 0.05.

### Generation of serum fractions

After collection of the whole blood in standard serum tubes, the blood was allowed to clot by leaving it undisturbed at room temperature for 20 min. The clot was separated from the serum by centrifuging at 2000×*g* for 10 min in a refrigerated centrifuge. Serum was carefully removed by pipetting and stored at − 80 °C until further fractioning. 100 kDa, 50 kDa and 10 kDa Amicon Ultra-0.5 mL Centrifugal Filters (Merck) were pre-rinsed with 0.5 ml 0.1 M NaOH at 14,000×*g* for 30 min to avoid glycerine interference in the analysis. Next, filters were rinsed with distilled water by spinning 0.5 mL distilled water for 30 min at 14,000×*g*. Every 30 min wash was followed by spinning the device in the inverted position at 1000×*g* for 2 min to remove the residual solution contained in the filter. 0.5 mL serum was transferred to the 100 kDa filter and centrifuged at 14,000×*g* for 30 min. The filtrate of this centrifugation step contained molecules with < 100 kDa. The concentrate (remainder of the serum in the filter) was collected by placing the filter device upside down and spinning for 1000×*g* for 2 min. The concentrate contained molecules with > 100 kDa. The filtrate of the 100 kDa filter was then transferred to the 50 kDa filter and centrifuged at 14,000×*g* for 30 min. The filtrate of this centrifugation step contained molecules with < 50 kDa. The concentrate of the 50 kDa filter was collected by placing the filter device upside down and spinning for 1000×*g* for 2 min. The concentrate contained molecules with 50–100 kDa. The filtrate of the 50 kDa filter was transferred to the 10 kDa filter and centrifuged at 14,000×*g* for 30 min. The filtrate of this centrifugation step contained molecules with < 10 kDa. The concentrate of the 10 kDa filter was collected by placing the filter device upside down and spinning for 1000×*g* for 2 min. The concentrate contained molecules with 10–50 kDa and was stored at − 80 °C until Raman spectroscopy.

### Raman spectroscopy and spectral analysis

Confocal Raman spectra were measured using a LabRam HR Evolution spectrometer (Horiba Scientific) at room temperature under ambient conditions in a backscattering geometry. The biopsies and podocytes were placed on platinum-coated silicon wafers and probed with a 633 nm HeNe laser focused tightly through a ×100 objective (numerical aperture 0.9) resulting in a laser beam diameter of ~ 1 µm and a laser power of ~ 11 mW on samples. A 300 grooves/mm diffraction grating and a charge-coupled device cooled at ~ 60 ^o^ C were used to detect the Raman scattered light at 1.4 cm^−1^ resolution. Up to 3600 spectra (substrate excluded) were obtained by mapping for each type of sample: biopsies at time of transplantation and podocytopathy relapse as well as podocytes treated with control and FSGS serum. The serum samples protected by a cover glass were measured with a 532 nm laser through a 50× (numerical aperture 0.5) and a laser power of ~ 6 mW. Spectral analysis including polyline baseline fitting, threshold removal of substrate spectra, calculation of mean spectra, and anomaly detection in the range of 350 cm^−1^ to 1800 cm^−1^ were performed using LabSpec 6 (Horiba Scientific) and in-house Python scripts.

Formalin-fixed paraffin embedded (FFPE) sections were transferred on platinum-coated silicon wafers followed by dewaxing with xylene over a period of 24 h and replacing the xylene after 20 and 40 min. The xylene was then rinsed in isopropanol for 2 × 2 min and then air dried at room temperature. A Raman spectroscopy map was generated from two measurements belonging to the biopsy at the time of kidney transplantation (0-biopsy) and two measurements belonging to the biopsy at the time of FSGS recurrence in the transplant. In total, 3600 spectra consisting of 1025 different wavelengths were available for images. As all Raman spectra from the biopsies derived from biological material with the same molecular components the pathogenicity was expressed to exclusively cause different ratios in the corresponding Raman so that only small differences in the amplitude of the spectra can be observed. In order to evaluate the difference in Raman spectroscopy quantitatively and with spatial resolution, we used a machine learning approach. An anomaly detection using a One-Class SVM (O-SVM) which has been successfully employed in a variety of medical and industrial applications^76^ was pursued. O-SVM allows learning a decision boundary or a hypersphere in the high dimensional feature space solely by using the spectra of the physiological samples. Parts of the Raman map, that only contained substrate or noise signal where removed, to not deteriorate the O-SVM and to focus only on the parts of the Raman map that contains signal information. Consequently, the hypersphere approximately matched the distribution of the spectra of the physiological samples. This separation hypothesis was tested with the spectra of the pathological samples (FSGS recurrence). For these Raman spectra, a confidence score could be determined using the O-SVM, which describes whether the spectrum can be considered as anomaly, whereby the degree of the anomaly increases with increasing distance to the hypersphere. The baseline corrected Raman spectra were used as features, which cover a dimension of 1025 wavelengths. To reduce the dimension of the feature vector, principal component analysis (PCA) was further employed so that 95% of the variance contained within the spectra was covered. This resulted in a dimensionality of 75 PCA components per spectrum, which represent the feature vectors that were subsequently used for generating the decision hyperplane of the One-Class SVM. Consequently, 7200 × 75 physiological samples were available for training the O-SVM, as well as potentially 7200 pathological samples for the determination of the anomaly. The parameters of the O-SVM ($\nu ,\gamma$) that characterize the decision boundary and balance two properties were empirically set to 0.05 and 0.0005.

### Electron microscopy

For electron microscopy a piece of the human renal biopsy was fixed overnight in 4% formalin, post-fixed with 1% OsO_4_ (90 min) and stained for 1 h with 1% UranyLess (Science services GmbH, Munich, Germany). After dehydration tissue blocks were embedded in Araldite Renlam M1 resin (Serva Electrophoresis GmbH, Heidelberg, Germany). 80 nm ultrathin sections were cut on an UC7 ultramicrotome (Leica, Wetzlar, Germany) and rinsed in lead citrate buffer for contrasting before analysis using an Leo912 transmission electron microscope (Zeiss, Oberkochen, Germany).

### Immunofluorescence staining of human kidney biopsies

Formalin-fixed, paraffin embedded human renal biopsies were cut into sections of 2 µm and transferred either on glass slides or on platinum-coated silicon wafers. After deparaffinization sections on platinum-coated silicon wafers were used for Raman spectroscopy first. Next, antigen retrieval was done using target retrieval solution pH 6 (Dako Deutschland GmbH, Hamburg, Germany) and cooking in a pressure cooker for 2.5 min at 110 °C. After blocking for 30 min with 5% skim milk in 50 mM Tris pH 7.4 sections were incubated overnight at 4 °C for immunofluorescence staining and at room temperature for immunohistochemistry using the following antibodies diluted in 1% BSA in 50 mM Tris pH 7.4: Pax8, a rabbit polyclonal antibody against paired box gene 8 (Cell Marque, Rocklin, CA, USA) 1:50; synaptopodin, a mouse monoclonal antibody against rat synaptopodin (clone G1D4, Progen Biotechnik GmbH, Heidelberg, Germany) 1:100. Double-staining of Pax8 with podocyte marker synaptopodin was performed using fluorescence labelled secondary antibodies diluted 1:200 in 50 mM Tris pH 7.4 and incubated for 30 min at room temperature. The following secondary antibodies were used: donkey anti-mouse IgG Alexa Fluor 647 (A31571) and donkey anti-rabbit IgG Alexa Fluor 568 (A10042) both from Thermo Fisher Scientific (Waltham, MA, USA). Nuclei of cells were stained with DAPI in distilled water (0.2 µg/ml) for 5 min followed by rinsing in Tris buffer. Finally, sections were covered with TrueView mounting kit (Vector laboratories) and analyzed using laser scanning confocal microscopy (LSM Zeiss 710 and Zen software, Zeiss GmbH, Jena, Germany) and Image-J software. Negative controls for immunostaining included either deletion or substitution of the primary antibody with equivalent concentrations of an irrelevant murine monoclonal antibody or pre-immune rabbit IgG.

### Statistics

PCA was performed for Raman analysis as described above. T-test was performed for zebrafish experiments to screen for statistical significance.

## Supplementary Information

Supplementary Information 1.

Supplementary Information 2.

Supplementary Information 3.

Supplementary Information 4.

Supplementary Information 5.

Supplementary Information 6.
