# Author Correction: Novel diagnostic and therapeutic techniques reveal changed metabolic profiles in recurrent focal segmental glomerulosclerosis

**DOI:** 10.1038/s41598-021-89610-9

**Published:** 2021-05-17

**Authors:** Janina Müller-Deile, George Sarau, Ahmed M. Kotb, Christian Jaremenko, Ulrike E. Rolle-Kampczyk, Christoph Daniel, Stefan Kalkhof, Silke H. Christiansen, Mario Schiffer

**Affiliations:** 1 Department of Nephrology and Hypertension, Friedrich-Alexander-University (FAU) Erlangen-Nuremberg, Erlangen, Germany; 2 Fraunhofer Institute for Ceramic Technologies and Systems IKTS, Dresden, Germany; 3 Leuchs Emeritus Group, Max Planck Institute for the Science of Light, Erlangen, Germany; 4 Institute for Nanotechnology and Correlative Microscopy eV INAM, Forchheim, Germany; 5 Department of Anatomy and Histology, Faculty of Veterinary Medicine, Assiut University, Asyût, Egypt; 6 Institute of Optics, Information and Photonics, Friedrich-Alexander-University (FAU) Erlangen-Nuremberg, Erlangen, Germany; 7 Department Molecular Systems Biology, Helmholtz Centre for Environmental Research, Leipzig, Germany; 8 Department of Nephropathology, Friedrich-Alexander-University (FAU) Erlangen-Nuremberg, Erlangen, Germany; 9 Institute for Bioanalysis, University of Applied Sciences Coburg, Coburg, Germany; 10 Department of Molecular Systems Biology, Helmholtz-Centre for Environmental Research-UFZ, Leipzig, Germany; 11 Physics Department, Freie Universität Berlin, Berlin, Germany

Correction to: *Scientific Reports* 10.1038/s41598-021-83883-w, published online 25 February 2021

The original version of this Article contained an error in Figure 2B, where an incorrect panel was mistakenly included as panel (f). This panel has been removed, and cannot be replaced due to antibody issues affecting reproducibility. The original Figure 2 and accompanying legend appear below.

**Figure 2:**
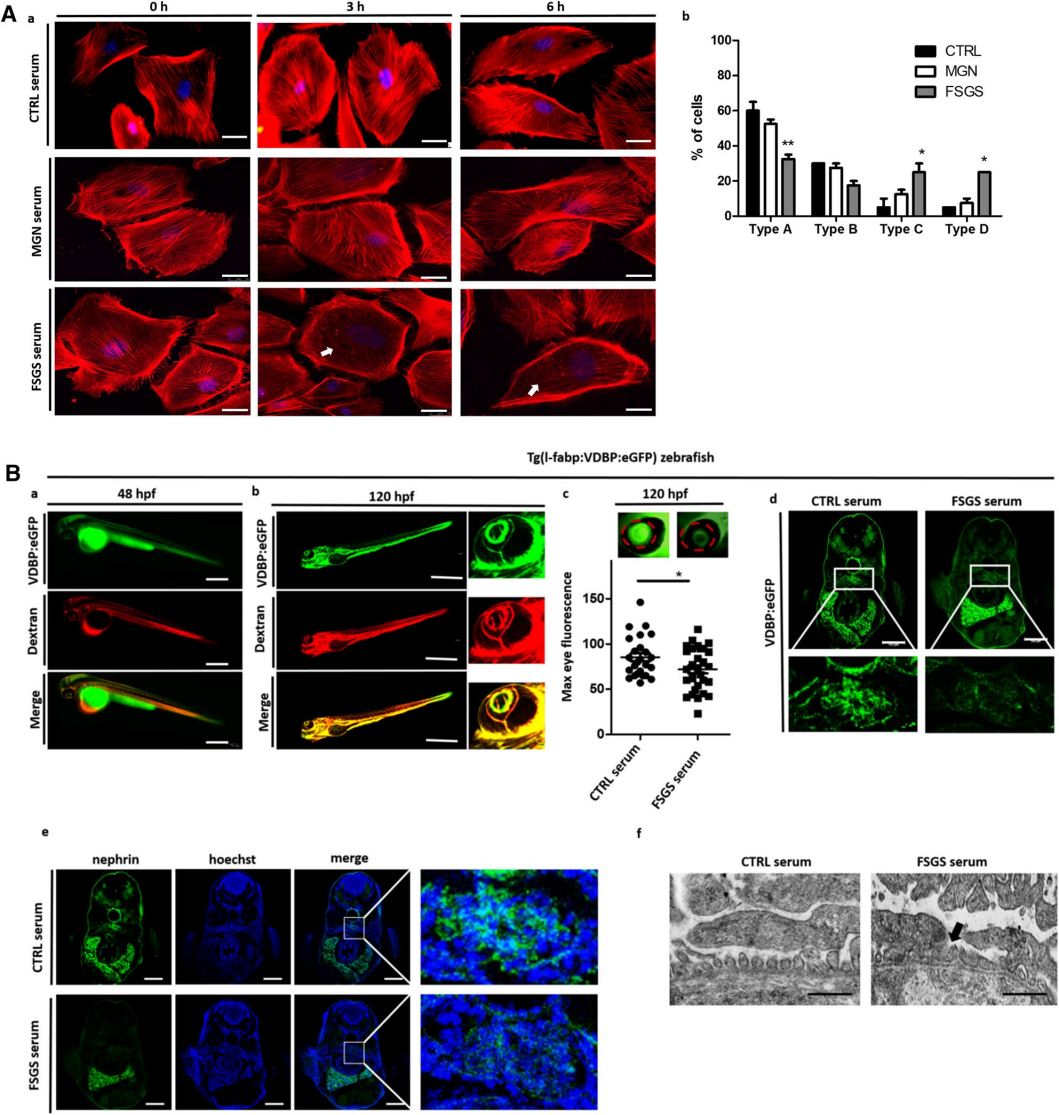
Cell culture and zebrafish assay to detect morphological and functional effects of unknown circulating permeability factors in FSGS. (**A**)—(a) Representative images of cultured differentiated human podocytes treated with 10% serum from a healthy control (CTRL serum), the patient with recurrent FSGS in the kidney transplant and a patient with membranous glomerulonephritis (incubation for 0 h, 3 h and 6 h). Cells were stained with phalloidin for cytoskeleton labeling. White arrows indicate cytoskeleton rearrangement. Scale bar = 25 µm. (b) Quantification of stress fiber formation in podocytes after treatment with different sera of patients. Type A: more than 90% of cell area filled with thick cables; type B: at least 2 thick cables running under nucleus and rest of cell area filled with fine cables; type C: no thick cables, but some cables present; type D: no cables visible in the central area of the cell. (**B**) Zebrafish assay for the detection of circulating permeability factors. (a) Representative image of a transgenic Tg(l-fabp:VDBP:eGFP) zebrafish larvae (VDBP:eGFP) injected with serum: dextran texas red into the zebrafish circulation (Dextran c.v. injection) at 48 hpf. Proper injection leads to red fluorescence of the zebrafish vascularization. Expression of the green fluorescent vitamin D binding protein just started. Scale bar = 500 µm. (b) At 120 hpf injected Tg(l-fabp:VDBP:eGFP) zebrafish express green fluorescent vitamin D binding protein (VDBP:eGFP) in the circulation. Red fluorescent serum: dextran mixture is still detectable in the circulation (dextran) and merges with the green fluorescent vitamin D binding protein (merge). The zebrafish eye is enlarged to show the retinal plexus. (c) Tg(l-fabp:VDBP:eGFP) transgenic zebrafish can be used to indirectly monitor proteinuria. Loss of green fluorescent protein in FSGS serum injected fish leads to reduced GFP signal in the retinal vessels where it can easily be quantified. Quantification of loss of fluorescent vitamin D binding proteins was done by measuring maximum GFP fluorescence in the retinal vessel plexus of Tg(l-fabp:VDBP:eGFP) zebrafish larvae at 120 hpf. Zebrafish larvae were injected with serum: dextran mixture from a healthy control and from a patient with FSGS recurrence in the kidney transplant at 48 hpf. *p < 0.05. n = 107. Scale bar = 500 µm. (d) Cryo sections of Tg(l-fabp:VDBP:eGFP) transgenic zebrafish larvae at 120 hpf showing systemic decrease in VDBP:eGFP in the systemic vascular system in FSGS serum injected zebrafish as a hint for proteinuria. Zebrafish were injected with serum from CTRL or FSGS patient at the time of disease recurrence. Scale bar = 100 µm. (e) Immunofluorescence staining for nephrin in FSGS and CTRL serum injected zebrafish at 120 hpf. Zebrafish larvae were injected with serum from a healthy control and from a patient with FSGS recurrence in the kidney transplant at 48 hpf. Scale bar = 100 µm. (f) Electron microscopy picture of the glomerular filtration barrier of 5 day old zebrafish larvae that were injected with either serum of the patient from the time of FSGS recurrence or with CTRL serum at 48 hpf. Black arrow head shows podocyte effacement. Scale bar = 500 nm. hpf: hours post fertilization, VDBP: Vitamin D binding protein.

As a result, in the legend of Figure 2,

“(e) Immunofluorescence staining for nephrin in FSGS and CTRL serum injected zebrafish at 120 hpf. Zebrafish larvae were injected with serum from a healthy control and from a patient with FSGS recurrence in the kidney transplant at 48 hpf. Scale bar = 100 μm. (f) Electron microscopy picture of the glomerular filtration barrier of 5 day old zebrafish larvae that were injected with either serum of the patient from the time of FSGS recurrence or with CTRL serum at 48 hpf.”

now reads:

“(e) Electron microscopy picture of the glomerular filtration barrier of 5 day old zebrafish larvae that were injected with either serum of the patient from the time of FSGS recurrence or with CTRL serum at 48 hpf.”

Additionally, in the Results section under the subheading ‘Ex vivo tests to detect morphological changes in podocytes caused by FSGS serum’,

“We performed immunofluorescent staining and transmission electron microscopy of the zebrafish pronephros of fish that were injected with the serum of the FSGS patient from the time of disease recurrence and could show reduced nephrin expression (Fig. 2Be) and podocyte effacement compared to CTRL serum injected zebrafish (Fig. 2Bf).”

now reads:

“We performed transmission electron microscopy of the zebrafish pronephros of fish that were injected with the serum of the FSGS patient from the time of disease recurrence and could show podocyte effacement compared to CTRL serum injected zebrafish (Fig. 2Be).”

Furthermore, in the Discussion section,

“We injected serum of patient with recurrent FSGS in the cardinal vein of the zebrafish and detected a significant loss of plasma proteins, reduced nephrin and partial podocyte effacement 3 days later.”

now reads:

“We injected serum of patient with recurrent FSGS in the cardinal vein of the zebrafish and detected a significant loss of plasma proteins and partial podocyte effacement 3 days later.”

Finally, in the Methods section, the subsection entitled ‘Immunofluorescence staining of zebrafish larvae’ has been removed.

“For paraffin tissue sections, zebrafish larvae were fixed in 2% PFA for 1 day, dehydrated in ascending concentrations of ethanol in PBS (25%, 50%, 70%, and 100%) and transferred in xylene (100%) for 5 min before embedding in paraffin (60 °C) overnight. With a rotational microtome (Leica SM 2000 R) sections of 5 μm were cut, incubated in ethanol (100%, 70%, 50%, and 25%). For cryosections larvae were fixed in 2% PFA for 2 h followed by an overnight incubation in 30% saccharose at 4 °C. After embedding the larvae in Tissue-Tek (Sakura, Staufen, Germany), sectsions (20 μm, 60 μm) were cut using a Leica CM 1950 microtome and stained with antibodies. The following primary antibodies were used: anti-zebrafish nephrin (zNephrin, Innovagen, Sweden) 1:8000, Alexa Fluor 488 goat anti-rabbit (life technology) 1:500.”

These errors have now been corrected in the PDF and HTML versions of the Article.

