# Supplementary material for: Novel diagnostic and therapeutic techniques reveal changed metabolic profiles in recurrent focal segmental glomerulosclerosis
Source: Sci Rep. 2021 Feb 25;11:4577. doi: 10.1038/s41598-021-83883-w (PMC7907124; doi:10.1038/s41598-021-83883-w)
Supplement: Supplementary file 1 — Supplementary Information 1. [file 41598_2021_83883_MOESM1_ESM.pdf]

Novel diagnostic and therapeutic techniques reveal changed metabolic profiles in recurrent Focal Segmental Glomerulosclerosis

Janina Müller-Deile[1*], George Sarau[2,3,4], Ahmed M Kotb[1,5], Christian Jaremenko[4,6], Ulrike E. Rolle-Kampczyk[7], Christoph Daniel[8], Stefan Kalkhof[9,10], Silke H. Christiansen[2,3,4,11] and Mario Schiffer[1]

[1]Department of Nephrology and Hypertension, Friedrich-Alexander-University (FAU) Erlangen-Nuremberg, Erlangen, Germany
[2]Fraunhofer Institute for Ceramic Technologies and Systems IKTS, Dresden, Germany
[3]Leuchs Emeritus Group, Max Planck Institute for the Science of Light, Erlangen, Germany
[4]Institute for Nanotechnology and Correlative Microscopy eV INAM, Forchheim, Germany
[5]Department of Anatomy and Histology, Faculty of Veterinary Medicine, Assiut University, Assiut, Egypt
[6]Institute of Optics, Information and Photonics, Friedrich-Alexander-University (FAU) Erlangen-Nuremberg, Erlangen, Germany
[7]Protein biomarker unit, Department of Therapy validation, Fraunhofer Institute for Cell Therapy and Immunology, Leipzig, Germany
[8]Department of Nephropathology, Friedrich-Alexander-University (FAU) Erlangen-Nuremberg, Erlangen, Germany
[9]Institute for Bioanalysis, University of Applied Sciences Coburg, Coburg, Germany
[10]Department of Molecular Systems Biology, Helmholtz-Centre for Environmental Research - UFZ, Leipzig, Germany
[11]Physics Department, Freie Universität Berlin, Berlin, Germany

Supplementary material

Supplementary Fig. 1

Zebrafish assay for proteinuria detection after injection of FSGS serum at time of disease recurrence and at the time of remission.

Quantification of loss of fluorescent vitamin D binding proteins was done by measuring maximum GFP fluorescence in the retinal vessel plexus of Tg(l-fabp:VDBP:eGFP) zebrafish larvae at 120 hpf. Zebrafish larvae were injected with serum: buffer mixture from a healthy control and from a patient with FSGS at the time of disease recurrence and at the time of FSGS remission. * = $p < 0.05$. hpf: hours post fertilization. n = 29.

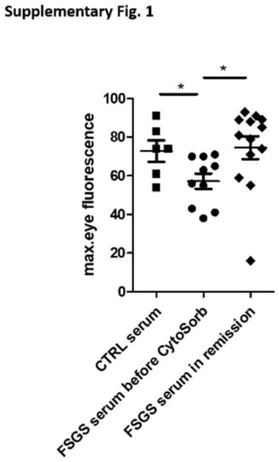

Supplementary table 1

Raman shift and Raman intensity of Raman measurement of <50 kDa serum fractions of a control person, FSGS patient at the time of disease recurrence and FSGS patient at the time of remission.

Supplementary table 2

Raman shift and Raman intensity of Raman measurement of podocytes treated with CTRL serum and FSGS serum.

Supplementary table 3

Raman shift and Raman intensity of Raman measurement of 0 biopsy and FSGS recurrence biopsy.

Supplementary table 4

Log2 (FSGS patient / stable transplanted control) values of metabolites measured by mass spectrometry before 1. CytoSorb, after 1. CytoSorb, mean of before 1. CytoSorb and after 1. CytoSorb (mean at recurrence), before CytoSorb at remission, after CytoSorb at remission and mean of before CytoSorb at remission, and after CytoSorb at remission (mean at remission). Difference in between Log2 (FSGS mean at remission / stable transplanted control) versus Log2 (FSGS mean at recurrence / stable transplanted control) is given together with p-value (paired, 2-sided).

Supplementary table 5

Values of metabolites (in μM) measured by mass spectrometry in our FSGS patient at time of disease recurrence (two different measurements) and in 10 healthy subjects of the EPIC-study. P-value states if difference in FSGS patient at time of disease recurrence versus healthy controls was significant.
