# Supplementary material for: Novel diagnostic and therapeutic techniques reveal changed metabolic profiles in recurrent focal segmental glomerulosclerosis
Source: Sci Rep. 2021 Feb 25;11:4577. doi: 10.1038/s41598-021-83883-w (PMC7907124; doi:10.1038/s41598-021-83883-w)
Supplement: Supplementary file 2 — Supplementary Information 2. [file 41598_2021_83883_MOESM2_ESM.pdf]

| < 50 kDa CTRL serum | | < 50 kDa FSGS serum at recurrence | | < 50 kDa FSGS serum at remission | |
|---|---|---|---|---|---|
| Raman Shift (cm-1) | Raman intensity (arb. units) | Raman Shift (cm-1) | Raman intensity (arb. units) | Raman Shift (cm-1) | Raman intensity (arb. units) |
| 300 | 634 | 300 | 776 | 300 | 634 |
| 302 | 537 | 302 | 520 | 302 | 534 |
| 305 | 520 | 305 | 468 | 305 | 479 |
| 307 | 361 | 307 | 346 | 307 | 375 |
| 309 | 353 | 309 | 336 | 309 | 318 |
| 311 | 324 | 311 | 246 | 311 | 256 |
| 313 | 268 | 313 | 262 | 313 | 196 |
| 316 | 172 | 316 | 184 | 316 | 137 |
| 318 | 208 | 318 | 130 | 318 | 125 |
| 320 | -22 | 320 | 67 | 320 | 102 |
| 322 | 64 | 322 | 1 | 322 | 28 |
| 325 | 95 | 325 | 82 | 325 | -4 |
| 327 | -18 | 327 | 11 | 327 | 22 |
| 329 | -11 | 329 | 0 | 329 | -33 |
| 331 | -9 | 331 | -21 | 331 | 38 |
| 334 | -47 | 334 | 0 | 334 | 25 |
| 336 | 83 | 336 | 10 | 336 | -43 |
| 338 | -13 | 338 | 91 | 338 | 1 |
| 340 | 190 | 340 | -6 | 340 | 42 |
| 343 | 145 | 343 | 50 | 343 | 42 |
| 345 | 139 | 345 | 43 | 345 | 23 |
| 347 | 131 | 347 | 110 | 347 | 44 |
| 349 | 167 | 349 | 148 | 349 | 96 |
| 352 | 262 | 352 | 65 | 352 | 120 |
| 354 | 215 | 354 | 183 | 354 | 198 |
| 356 | 282 | 356 | 202 | 356 | 140 |
| 358 | 284 | 358 | 131 | 358 | 176 |
| 361 | 233 | 361 | 199 | 361 | 123 |
| 363 | 261 | 363 | 178 | 363 | 193 |
| 365 | 364 | 365 | 191 | 365 | 218 |

| | | | | | |
|---|---|---|---|---|---|
| 367 | 142 | 367 | 216 | 367 | 249 |
| 369 | 363 | 369 | 252 | 369 | 272 |
| 372 | 302 | 372 | 273 | 372 | 202 |
| 374 | 203 | 374 | 379 | 374 | 236 |
| 376 | 327 | 376 | 358 | 376 | 250 |
| 378 | 370 | 378 | 384 | 378 | 270 |
| 381 | 368 | 381 | 361 | 381 | 251 |
| 383 | 303 | 383 | 455 | 383 | 340 |
| 385 | 431 | 385 | 380 | 385 | 300 |
| 387 | 410 | 387 | 476 | 387 | 238 |
| 390 | 335 | 390 | 453 | 390 | 330 |
| 392 | 452 | 392 | 430 | 392 | 254 |
| 394 | 484 | 394 | 569 | 394 | 372 |
| 396 | 434 | 396 | 622 | 396 | 425 |
| 398 | 605 | 398 | 680 | 398 | 424 |
| 401 | 589 | 401 | 784 | 401 | 408 |
| 403 | 579 | 403 | 793 | 403 | 365 |
| 405 | 566 | 405 | 1009 | 405 | 541 |
| 407 | 687 | 407 | 1092 | 407 | 493 |
| 410 | 592 | 410 | 1152 | 410 | 527 |
| 412 | 726 | 412 | 1262 | 412 | 538 |
| 414 | 686 | 414 | 1304 | 414 | 558 |
| 416 | 756 | 416 | 1371 | 416 | 595 |
| 418 | 749 | 418 | 1294 | 418 | 668 |
| 421 | 987 | 421 | 1254 | 421 | 656 |
| 423 | 882 | 423 | 1284 | 423 | 760 |
| 425 | 889 | 425 | 1154 | 425 | 686 |
| 427 | 862 | 427 | 1169 | 427 | 762 |
| 430 | 1038 | 430 | 1202 | 430 | 776 |
| 432 | 873 | 432 | 1226 | 432 | 829 |
| 434 | 1000 | 434 | 1101 | 434 | 868 |
| 436 | 1049 | 436 | 1149 | 436 | 835 |
| 438 | 1129 | 438 | 1097 | 438 | 862 |

| | | | | | |
|---|---|---|---|---|---|
| 441 | 1019 | 441 | 1086 | 441 | 903 |
| 443 | 1042 | 443 | 1085 | 443 | 839 |
| 445 | 1067 | 445 | 1147 | 445 | 859 |
| 447 | 1007 | 447 | 1130 | 447 | 852 |
| 449 | 1043 | 449 | 1096 | 449 | 861 |
| 452 | 1029 | 452 | 1156 | 452 | 862 |
| 454 | 929 | 454 | 1167 | 454 | 936 |
| 456 | 1098 | 456 | 1143 | 456 | 843 |
| 458 | 1103 | 458 | 1148 | 458 | 940 |
| 460 | 1022 | 460 | 1193 | 460 | 857 |
| 463 | 1017 | 463 | 1237 | 463 | 838 |
| 465 | 1056 | 465 | 1325 | 465 | 839 |
| 467 | 1024 | 467 | 1479 | 467 | 931 |
| 469 | 988 | 469 | 1545 | 469 | 875 |
| 472 | 945 | 472 | 1685 | 472 | 857 |
| 474 | 993 | 474 | 1866 | 474 | 923 |
| 476 | 1098 | 476 | 1945 | 476 | 903 |
| 478 | 1064 | 478 | 2007 | 478 | 921 |
| 480 | 1074 | 480 | 2192 | 480 | 948 |
| 483 | 1034 | 483 | 2144 | 483 | 863 |
| 485 | 1071 | 485 | 2038 | 485 | 898 |
| 487 | 1000 | 487 | 1997 | 487 | 909 |
| 489 | 1020 | 489 | 1893 | 489 | 910 |
| 491 | 1058 | 491 | 1811 | 491 | 883 |
| 494 | 995 | 494 | 1661 | 494 | 856 |
| 496 | 1071 | 496 | 1579 | 496 | 836 |
| 498 | 902 | 498 | 1417 | 498 | 782 |
| 500 | 1059 | 500 | 1391 | 500 | 834 |
| 502 | 943 | 502 | 1278 | 502 | 827 |
| 505 | 903 | 505 | 1180 | 505 | 802 |
| 507 | 886 | 507 | 1155 | 507 | 786 |
| 509 | 991 | 509 | 1103 | 509 | 779 |
| 511 | 940 | 511 | 1020 | 511 | 754 |

| | | | | | |
|---|---|---|---|---|---|
| 513 | 1021 | 513 | 1038 | 513 | 752 |
| 516 | 791 | 516 | 1037 | 516 | 785 |
| 518 | 853 | 518 | 1002 | 518 | 728 |
| 520 | 955 | 520 | 892 | 520 | 639 |
| 522 | 849 | 522 | 913 | 522 | 642 |
| 524 | 824 | 524 | 856 | 524 | 617 |
| 527 | 707 | 527 | 794 | 527 | 579 |
| 529 | 763 | 529 | 815 | 529 | 592 |
| 531 | 643 | 531 | 763 | 531 | 524 |
| 533 | 656 | 533 | 783 | 533 | 574 |
| 535 | 641 | 535 | 662 | 535 | 483 |
| 537 | 545 | 537 | 717 | 537 | 428 |
| 540 | 572 | 540 | 682 | 540 | 449 |
| 542 | 648 | 542 | 757 | 542 | 372 |
| 544 | 412 | 544 | 622 | 544 | 380 |
| 546 | 498 | 546 | 623 | 546 | 320 |
| 548 | 472 | 548 | 591 | 548 | 344 |
| 551 | 439 | 551 | 512 | 551 | 295 |
| 553 | 332 | 553 | 542 | 553 | 329 |
| 555 | 289 | 555 | 456 | 555 | 212 |
| 557 | 326 | 557 | 468 | 557 | 256 |
| 559 | 361 | 559 | 412 | 559 | 217 |
| 562 | 251 | 562 | 408 | 562 | 260 |
| 564 | 230 | 564 | 418 | 564 | 240 |
| 566 | 364 | 566 | 424 | 566 | 170 |
| 568 | 351 | 568 | 314 | 568 | 230 |
| 570 | 306 | 570 | 324 | 570 | 174 |
| 572 | 348 | 572 | 298 | 572 | 213 |
| 575 | 323 | 575 | 265 | 575 | 198 |
| 577 | 263 | 577 | 267 | 577 | 160 |
| 579 | 323 | 579 | 335 | 579 | 238 |
| 581 | 324 | 581 | 329 | 581 | 243 |
| 583 | 342 | 583 | 295 | 583 | 181 |

| | | | | | |
|---|---|---|---|---|---|
| 586 | 289 | 586 | 324 | 586 | 165 |
| 588 | 331 | 588 | 312 | 588 | 213 |
| 590 | 202 | 590 | 349 | 590 | 176 |
| 592 | 343 | 592 | 287 | 592 | 146 |
| 594 | 326 | 594 | 271 | 594 | 175 |
| 596 | 349 | 596 | 334 | 596 | 189 |
| 599 | 167 | 599 | 268 | 599 | 185 |
| 601 | 273 | 601 | 306 | 601 | 182 |
| 603 | 222 | 603 | 226 | 603 | 138 |
| 605 | 229 | 605 | 383 | 605 | 111 |
| 607 | 312 | 607 | 259 | 607 | 201 |
| 609 | 325 | 609 | 273 | 609 | 205 |
| 612 | 233 | 612 | 276 | 612 | 197 |
| 614 | 254 | 614 | 221 | 614 | 150 |
| 616 | 192 | 616 | 182 | 616 | 119 |
| 618 | 163 | 618 | 229 | 618 | 153 |
| 620 | 129 | 620 | 203 | 620 | 147 |
| 623 | 200 | 623 | 193 | 623 | 104 |
| 625 | 129 | 625 | 186 | 625 | 125 |
| 627 | 184 | 627 | 185 | 627 | 129 |
| 629 | 201 | 629 | 170 | 629 | 140 |
| 631 | 45 | 631 | 166 | 631 | 15 |
| 633 | 99 | 633 | 86 | 633 | 38 |
| 636 | -49 | 636 | 79 | 636 | 52 |
| 638 | 86 | 638 | -14 | 638 | 11 |
| 640 | 65 | 640 | 136 | 640 | -16 |
| 642 | 83 | 642 | 123 | 642 | 21 |
| 644 | 27 | 644 | 57 | 644 | 27 |
| 646 | 107 | 646 | 43 | 646 | -6 |
| 649 | 36 | 649 | 90 | 649 | -18 |
| 651 | 78 | 651 | 118 | 651 | 13 |
| 653 | 21 | 653 | 128 | 653 | -15 |
| 655 | 0 | 655 | 129 | 655 | 21 |

| | | | | | |
|---|---|---|---|---|---|
| 657 | -123 | 657 | 96 | 657 | -12 |
| 659 | 12 | 659 | 239 | 659 | -26 |
| 662 | 87 | 662 | 218 | 662 | 16 |
| 664 | 21 | 664 | 295 | 664 | 16 |
| 666 | 101 | 666 | 362 | 666 | 2 |
| 668 | 16 | 668 | 404 | 668 | 44 |
| 670 | 109 | 670 | 522 | 670 | 10 |
| 672 | 62 | 672 | 512 | 672 | 4 |
| 674 | 25 | 674 | 537 | 674 | 14 |
| 677 | 84 | 677 | 518 | 677 | 45 |
| 679 | 56 | 679 | 453 | 679 | 60 |
| 681 | 61 | 681 | 474 | 681 | 77 |
| 683 | 19 | 683 | 428 | 683 | 89 |
| 685 | 218 | 685 | 336 | 685 | 93 |
| 687 | 164 | 687 | 321 | 687 | 95 |
| 690 | 161 | 690 | 292 | 690 | 176 |
| 692 | 95 | 692 | 197 | 692 | 68 |
| 694 | 188 | 694 | 272 | 694 | 73 |
| 696 | 165 | 696 | 250 | 696 | 165 |
| 698 | 78 | 698 | 237 | 698 | 167 |
| 700 | 183 | 700 | 235 | 700 | 152 |
| 702 | 111 | 702 | 222 | 702 | 110 |
| 705 | 287 | 705 | 176 | 705 | 158 |
| 707 | 72 | 707 | 147 | 707 | 174 |
| 709 | 177 | 709 | 158 | 709 | 139 |
| 711 | 74 | 711 | 73 | 711 | 78 |
| 713 | 160 | 713 | 131 | 713 | 111 |
| 715 | 146 | 715 | 86 | 715 | 120 |
| 718 | 69 | 718 | 151 | 718 | 124 |
| 720 | 14 | 720 | 86 | 720 | 149 |
| 722 | 80 | 722 | 108 | 722 | 127 |
| 724 | 105 | 724 | 60 | 724 | 99 |
| 726 | 164 | 726 | 51 | 726 | 64 |

| | | | | | |
|---|---|---|---|---|---|
| 728 | 64 | 728 | 62 | 728 | 141 |
| 730 | 26 | 730 | 45 | 730 | 91 |
| 733 | 72 | 733 | 7 | 733 | 150 |
| 735 | 56 | 735 | -33 | 735 | 183 |
| 737 | 36 | 737 | -34 | 737 | 160 |
| 739 | -66 | 739 | -25 | 739 | 212 |
| 741 | 126 | 741 | -2 | 741 | 143 |
| 743 | 20 | 743 | -48 | 743 | 125 |
| 745 | 56 | 745 | 38 | 745 | 135 |
| 748 | 106 | 748 | -26 | 748 | 201 |
| 750 | 16 | 750 | 33 | 750 | 149 |
| 752 | 107 | 752 | -25 | 752 | 128 |
| 754 | 17 | 754 | 26 | 754 | 234 |
| 756 | 181 | 756 | -17 | 756 | 216 |
| 758 | 75 | 758 | -31 | 758 | 187 |
| 760 | 134 | 760 | 71 | 760 | 218 |
| 762 | 198 | 762 | -16 | 762 | 235 |
| 765 | 212 | 765 | 105 | 765 | 253 |
| 767 | 119 | 767 | 138 | 767 | 266 |
| 769 | 139 | 769 | 84 | 769 | 256 |
| 771 | 83 | 771 | 188 | 771 | 217 |
| 773 | 168 | 773 | 163 | 773 | 285 |
| 775 | 228 | 775 | 201 | 775 | 292 |
| 777 | 276 | 777 | 228 | 777 | 288 |
| 780 | 253 | 780 | 200 | 780 | 271 |
| 782 | 227 | 782 | 286 | 782 | 326 |
| 784 | 383 | 784 | 303 | 784 | 388 |
| 786 | 307 | 786 | 253 | 786 | 317 |
| 788 | 293 | 788 | 336 | 788 | 344 |
| 790 | 208 | 790 | 340 | 790 | 395 |
| 792 | 237 | 792 | 385 | 792 | 401 |
| 794 | 296 | 794 | 370 | 794 | 325 |
| 797 | 302 | 797 | 384 | 797 | 410 |

| | | | | | |
|---|---|---|---|---|---|
| 799 | 280 | 799 | 397 | 799 | 377 |
| 801 | 338 | 801 | 453 | 801 | 362 |
| 803 | 377 | 803 | 528 | 803 | 399 |
| 805 | 262 | 805 | 611 | 805 | 350 |
| 807 | 243 | 807 | 666 | 807 | 320 |
| 809 | 240 | 809 | 754 | 809 | 332 |
| 811 | 223 | 811 | 1002 | 811 | 374 |
| 814 | 369 | 814 | 1095 | 814 | 363 |
| 816 | 255 | 816 | 1200 | 816 | 352 |
| 818 | 256 | 818 | 1352 | 818 | 321 |
| 820 | 230 | 820 | 1230 | 820 | 311 |
| 822 | 194 | 822 | 1199 | 822 | 269 |
| 824 | 118 | 824 | 1079 | 824 | 237 |
| 826 | 109 | 826 | 1009 | 826 | 237 |
| 828 | 103 | 828 | 908 | 828 | 252 |
| 831 | 38 | 831 | 929 | 831 | 236 |
| 833 | 45 | 833 | 976 | 833 | 241 |
| 835 | 127 | 835 | 1045 | 835 | 199 |
| 837 | 149 | 837 | 1256 | 837 | 181 |
| 839 | 101 | 839 | 1408 | 839 | 222 |
| 841 | 104 | 841 | 1618 | 841 | 129 |
| 843 | 150 | 843 | 1816 | 843 | 176 |
| 845 | 124 | 845 | 1909 | 845 | 129 |
| 848 | 110 | 848 | 1982 | 848 | 167 |
| 850 | 52 | 850 | 2000 | 850 | 139 |
| 852 | 65 | 852 | 1941 | 852 | 151 |
| 854 | 93 | 854 | 1809 | 854 | 161 |
| 856 | 24 | 856 | 1729 | 856 | 191 |
| 858 | 69 | 858 | 1631 | 858 | 165 |
| 860 | 102 | 860 | 1465 | 860 | 185 |
| 862 | -21 | 862 | 1321 | 862 | 143 |
| 864 | 59 | 864 | 1228 | 864 | 107 |
| 867 | 121 | 867 | 1078 | 867 | 186 |

| | | | | | |
|---|---|---|---|---|---|
| 869 | 59 | 869 | 952 | 869 | 153 |
| 871 | 97 | 871 | 755 | 871 | 179 |
| 873 | 78 | 873 | 535 | 873 | 202 |
| 875 | 104 | 875 | 461 | 875 | 253 |
| 877 | -1 | 877 | 385 | 877 | 205 |
| 879 | 76 | 879 | 174 | 879 | 233 |
| 881 | 162 | 881 | 183 | 881 | 224 |
| 883 | 178 | 883 | 121 | 883 | 237 |
| 886 | 72 | 886 | 129 | 886 | 272 |
| 888 | 236 | 888 | 131 | 888 | 342 |
| 890 | 218 | 890 | 106 | 890 | 324 |
| 892 | 228 | 892 | 195 | 892 | 293 |
| 894 | 309 | 894 | 161 | 894 | 327 |
| 896 | 213 | 896 | 194 | 896 | 322 |
| 898 | 291 | 898 | 320 | 898 | 352 |
| 900 | 297 | 900 | 384 | 900 | 422 |
| 902 | 247 | 902 | 473 | 902 | 335 |
| 904 | 236 | 904 | 547 | 904 | 466 |
| 907 | 318 | 907 | 659 | 907 | 504 |
| 909 | 389 | 909 | 809 | 909 | 465 |
| 911 | 328 | 911 | 846 | 911 | 435 |
| 913 | 310 | 913 | 929 | 913 | 478 |
| 915 | 443 | 915 | 976 | 915 | 477 |
| 917 | 358 | 917 | 1003 | 917 | 469 |
| 919 | 290 | 919 | 980 | 919 | 471 |
| 921 | 362 | 921 | 1047 | 921 | 485 |
| 923 | 362 | 923 | 1007 | 923 | 465 |
| 925 | 470 | 925 | 986 | 925 | 474 |
| 928 | 381 | 928 | 899 | 928 | 419 |
| 930 | 329 | 930 | 753 | 930 | 450 |
| 932 | 349 | 932 | 655 | 932 | 366 |
| 934 | 337 | 934 | 447 | 934 | 375 |
| 936 | 323 | 936 | 322 | 936 | 417 |

| | | | | | |
|---|---|---|---|---|---|
| 938 | 333 | 938 | 198 | 938 | 403 |
| 940 | 292 | 940 | 132 | 940 | 367 |
| 942 | 252 | 942 | 90 | 942 | 372 |
| 944 | 376 | 944 | 56 | 944 | 377 |
| 946 | 222 | 946 | 96 | 946 | 378 |
| 949 | 150 | 949 | -10 | 949 | 360 |
| 951 | 268 | 951 | 83 | 951 | 307 |
| 953 | 275 | 953 | -15 | 953 | 302 |
| 955 | 205 | 955 | 0 | 955 | 298 |
| 957 | 266 | 957 | 7 | 957 | 280 |
| 959 | 238 | 959 | 13 | 959 | 276 |
| 961 | 233 | 961 | 0 | 961 | 303 |
| 963 | 177 | 963 | 67 | 963 | 301 |
| 965 | 201 | 965 | 154 | 965 | 248 |
| 967 | 185 | 967 | 134 | 967 | 271 |
| 969 | 135 | 969 | 199 | 969 | 275 |
| 971 | 272 | 971 | 221 | 971 | 261 |
| 974 | 194 | 974 | 234 | 974 | 231 |
| 976 | 248 | 976 | 367 | 976 | 257 |
| 978 | 274 | 978 | 310 | 978 | 216 |
| 980 | 225 | 980 | 271 | 980 | 244 |
| 982 | 252 | 982 | 165 | 982 | 189 |
| 984 | 158 | 984 | 171 | 984 | 225 |
| 986 | 283 | 986 | 194 | 986 | 178 |
| 988 | 165 | 988 | 168 | 988 | 176 |
| 990 | 273 | 990 | 213 | 990 | 177 |
| 992 | 155 | 992 | 197 | 992 | 145 |
| 994 | 246 | 994 | 210 | 994 | 151 |
| 996 | 324 | 996 | 240 | 996 | 125 |
| 999 | 299 | 999 | 280 | 999 | 158 |
| 1001 | 305 | 1001 | 319 | 1001 | 132 |
| 1003 | 240 | 1003 | 267 | 1003 | 129 |
| 1005 | 297 | 1005 | 316 | 1005 | 90 |

| | | | | | |
|---|---|---|---|---|---|
| 1007 | 302 | 1007 | 255 | 1007 | 87 |
| 1009 | 211 | 1009 | 207 | 1009 | 43 |
| 1011 | 264 | 1011 | 250 | 1011 | 27 |
| 1013 | 204 | 1013 | 272 | 1013 | 75 |
| 1015 | 165 | 1015 | 201 | 1015 | 69 |
| 1017 | 145 | 1017 | 217 | 1017 | 30 |
| 1019 | 167 | 1019 | 259 | 1019 | 43 |
| 1021 | 114 | 1021 | 397 | 1021 | 22 |
| 1023 | 96 | 1023 | 421 | 1023 | 18 |
| 1026 | 136 | 1026 | 397 | 1026 | 0 |
| 1028 | 92 | 1028 | 604 | 1028 | 28 |
| 1030 | 20 | 1030 | 682 | 1030 | -13 |
| 1032 | -68 | 1032 | 817 | 1032 | 29 |
| 1034 | 33 | 1034 | 1028 | 1034 | 0 |
| 1036 | -36 | 1036 | 1204 | 1036 | 28 |
| 1038 | 53 | 1038 | 1397 | 1038 | -12 |
| 1040 | 9 | 1040 | 1594 | 1040 | 24 |
| 1042 | 42 | 1042 | 1850 | 1042 | 54 |
| 1044 | -57 | 1044 | 1990 | 1044 | 10 |
| 1046 | 64 | 1046 | 2055 | 1046 | 49 |
| 1048 | 33 | 1048 | 2069 | 1048 | 33 |
| 1050 | 33 | 1050 | 2127 | 1050 | 71 |
| 1052 | -14 | 1052 | 2091 | 1052 | 37 |
| 1055 | 120 | 1055 | 2208 | 1055 | 65 |
| 1057 | 84 | 1057 | 2160 | 1057 | 98 |
| 1059 | 156 | 1059 | 2284 | 1059 | 110 |
| 1061 | 169 | 1061 | 2203 | 1061 | 57 |
| 1063 | 213 | 1063 | 2078 | 1063 | 77 |
| 1065 | 276 | 1065 | 2005 | 1065 | 117 |
| 1067 | 196 | 1067 | 1889 | 1067 | 123 |
| 1069 | 208 | 1069 | 1750 | 1069 | 127 |
| 1071 | 147 | 1071 | 1681 | 1071 | 110 |
| 1073 | 171 | 1073 | 1583 | 1073 | 103 |

| | | | | | |
|---|---|---|---|---|---|
| 1075 | 210 | 1075 | 1519 | 1075 | 142 |
| 1077 | 172 | 1077 | 1532 | 1077 | 138 |
| 1079 | 157 | 1079 | 1415 | 1079 | 160 |
| 1081 | 182 | 1081 | 1405 | 1081 | 138 |
| 1083 | 186 | 1083 | 1407 | 1083 | 117 |
| 1085 | 234 | 1085 | 1367 | 1085 | 145 |
| 1088 | 180 | 1088 | 1380 | 1088 | 199 |
| 1090 | 125 | 1090 | 1401 | 1090 | 183 |
| 1092 | 162 | 1092 | 1447 | 1092 | 146 |
| 1094 | 189 | 1094 | 1467 | 1094 | 175 |
| 1096 | 251 | 1096 | 1429 | 1096 | 99 |
| 1098 | 159 | 1098 | 1415 | 1098 | 127 |
| 1100 | 172 | 1100 | 1466 | 1100 | 159 |
| 1102 | 327 | 1102 | 1484 | 1102 | 205 |
| 1104 | 267 | 1104 | 1475 | 1104 | 183 |
| 1106 | 279 | 1106 | 1620 | 1106 | 160 |
| 1108 | 331 | 1108 | 1577 | 1108 | 185 |
| 1110 | 231 | 1110 | 1571 | 1110 | 185 |
| 1112 | 251 | 1112 | 1602 | 1112 | 190 |
| 1114 | 321 | 1114 | 1516 | 1114 | 200 |
| 1116 | 249 | 1116 | 1454 | 1116 | 148 |
| 1118 | 302 | 1118 | 1303 | 1118 | 172 |
| 1120 | 309 | 1120 | 1133 | 1120 | 169 |
| 1122 | 191 | 1122 | 1066 | 1122 | 121 |
| 1125 | 267 | 1125 | 862 | 1125 | 164 |
| 1127 | 219 | 1127 | 708 | 1127 | 204 |
| 1129 | 264 | 1129 | 589 | 1129 | 150 |
| 1131 | 191 | 1131 | 482 | 1131 | 155 |
| 1133 | 198 | 1133 | 449 | 1133 | 167 |
| 1135 | 174 | 1135 | 344 | 1135 | 136 |
| 1137 | 151 | 1137 | 247 | 1137 | 80 |
| 1139 | 189 | 1139 | 241 | 1139 | 132 |
| 1141 | 32 | 1141 | 214 | 1141 | 76 |

| | | | | | |
|---|---|---|---|---|---|
| 1143 | 98 | 1143 | 157 | 1143 | 99 |
| 1145 | 158 | 1145 | 155 | 1145 | 64 |
| 1147 | 32 | 1147 | 141 | 1147 | 99 |
| 1149 | 95 | 1149 | 91 | 1149 | 45 |
| 1151 | 163 | 1151 | 120 | 1151 | 75 |
| 1153 | 95 | 1153 | 64 | 1153 | 71 |
| 1155 | 14 | 1155 | 51 | 1155 | 20 |
| 1157 | -4 | 1157 | 118 | 1157 | 0 |
| 1159 | 91 | 1159 | 31 | 1159 | 9 |
| 1161 | 11 | 1161 | 69 | 1161 | 54 |
| 1163 | 83 | 1163 | 66 | 1163 | 77 |
| 1165 | 109 | 1165 | 101 | 1165 | 34 |
| 1167 | -34 | 1167 | -10 | 1167 | 20 |
| 1170 | 31 | 1170 | 4 | 1170 | 4 |
| 1172 | 20 | 1172 | 21 | 1172 | 6 |
| 1174 | 9 | 1174 | -19 | 1174 | 7 |
| 1176 | 29 | 1176 | -13 | 1176 | -17 |
| 1178 | -61 | 1178 | -6 | 1178 | 2 |
| 1180 | 61 | 1180 | 1 | 1180 | -1 |
| 1182 | -12 | 1182 | 40 | 1182 | 6 |
| 1184 | 4 | 1184 | -25 | 1184 | -11 |
| 1186 | -23 | 1186 | 72 | 1186 | -37 |
| 1188 | -1 | 1188 | 74 | 1188 | 6 |
| 1190 | -21 | 1190 | 132 | 1190 | 22 |
| 1192 | -35 | 1192 | 244 | 1192 | 31 |
| 1194 | 92 | 1194 | 220 | 1194 | -25 |
| 1196 | 75 | 1196 | 324 | 1196 | 31 |
| 1198 | 48 | 1198 | 298 | 1198 | 15 |
| 1200 | 9 | 1200 | 334 | 1200 | 36 |
| 1202 | 69 | 1202 | 373 | 1202 | -3 |
| 1204 | 56 | 1204 | 448 | 1204 | 22 |
| 1206 | 104 | 1206 | 548 | 1206 | 37 |
| 1208 | -51 | 1208 | 516 | 1208 | 41 |

| | | | | | |
|---|---|---|---|---|---|
| 1210 | 144 | 1210 | 553 | 1210 | 31 |
| 1212 | 147 | 1212 | 608 | 1212 | 36 |
| 1214 | 130 | 1214 | 648 | 1214 | 82 |
| 1216 | 167 | 1216 | 642 | 1216 | 72 |
| 1218 | 72 | 1218 | 712 | 1218 | 84 |
| 1220 | 86 | 1220 | 709 | 1220 | 16 |
| 1222 | 147 | 1222 | 729 | 1222 | 50 |
| 1224 | 137 | 1224 | 768 | 1224 | 116 |
| 1226 | 183 | 1226 | 832 | 1226 | 25 |
| 1228 | 142 | 1228 | 828 | 1228 | 75 |
| 1230 | 226 | 1230 | 892 | 1230 | 87 |
| 1233 | 180 | 1233 | 916 | 1233 | 58 |
| 1235 | 236 | 1235 | 918 | 1235 | 87 |
| 1237 | 260 | 1237 | 1082 | 1237 | 105 |
| 1239 | 231 | 1239 | 1103 | 1239 | 124 |
| 1241 | 221 | 1241 | 1107 | 1241 | 96 |
| 1243 | 169 | 1243 | 1146 | 1243 | 104 |
| 1245 | 206 | 1245 | 1153 | 1245 | 122 |
| 1247 | 189 | 1247 | 1234 | 1247 | 84 |
| 1249 | 121 | 1249 | 1320 | 1249 | 99 |
| 1251 | 210 | 1251 | 1296 | 1251 | 128 |
| 1253 | 256 | 1253 | 1294 | 1253 | 122 |
| 1255 | 179 | 1255 | 1414 | 1255 | 132 |
| 1257 | 209 | 1257 | 1308 | 1257 | 92 |
| 1259 | 295 | 1259 | 1317 | 1259 | 142 |
| 1261 | 226 | 1261 | 1329 | 1261 | 77 |
| 1263 | 156 | 1263 | 1338 | 1263 | 110 |
| 1265 | 259 | 1265 | 1309 | 1265 | 121 |
| 1267 | 212 | 1267 | 1395 | 1267 | 133 |
| 1269 | 289 | 1269 | 1289 | 1269 | 118 |
| 1271 | 246 | 1271 | 1353 | 1271 | 125 |
| 1273 | 196 | 1273 | 1331 | 1273 | 64 |
| 1275 | 257 | 1275 | 1331 | 1275 | 99 |

| | | | | | |
|---|---|---|---|---|---|
| 1277 | 244 | 1277 | 1275 | 1277 | 43 |
| 1279 | 221 | 1279 | 1243 | 1279 | 66 |
| 1281 | 249 | 1281 | 1144 | 1281 | 113 |
| 1283 | 203 | 1283 | 1143 | 1283 | 134 |
| 1285 | 261 | 1285 | 1118 | 1285 | 86 |
| 1287 | 201 | 1287 | 1089 | 1287 | 38 |
| 1289 | 183 | 1289 | 1042 | 1289 | 83 |
| 1291 | 194 | 1291 | 1087 | 1291 | 109 |
| 1293 | 263 | 1293 | 1014 | 1293 | 120 |
| 1295 | 135 | 1295 | 1001 | 1295 | 76 |
| 1297 | 167 | 1297 | 940 | 1297 | 92 |
| 1299 | 210 | 1299 | 995 | 1299 | 65 |
| 1301 | 216 | 1301 | 917 | 1301 | 80 |
| 1303 | 173 | 1303 | 967 | 1303 | 87 |
| 1305 | 266 | 1305 | 999 | 1305 | 44 |
| 1307 | 272 | 1307 | 966 | 1307 | 113 |
| 1309 | 268 | 1309 | 948 | 1309 | 115 |
| 1311 | 213 | 1311 | 1023 | 1311 | 115 |
| 1313 | 319 | 1313 | 999 | 1313 | 126 |
| 1315 | 319 | 1315 | 1017 | 1315 | 118 |
| 1317 | 245 | 1317 | 1037 | 1317 | 122 |
| 1319 | 262 | 1319 | 1073 | 1319 | 128 |
| 1321 | 283 | 1321 | 1027 | 1321 | 118 |
| 1323 | 287 | 1323 | 1072 | 1323 | 128 |
| 1325 | 253 | 1325 | 1100 | 1325 | 141 |
| 1327 | 287 | 1327 | 1010 | 1327 | 116 |
| 1329 | 333 | 1329 | 1047 | 1329 | 144 |
| 1331 | 302 | 1331 | 1014 | 1331 | 150 |
| 1333 | 289 | 1333 | 1005 | 1333 | 147 |
| 1335 | 387 | 1335 | 1088 | 1335 | 142 |
| 1337 | 419 | 1337 | 1075 | 1337 | 165 |
| 1339 | 369 | 1339 | 1060 | 1339 | 160 |
| 1341 | 402 | 1341 | 1057 | 1341 | 198 |

| | | | | | |
|---|---|---|---|---|---|
| 1343 | 433 | 1343 | 1116 | 1343 | 181 |
| 1345 | 338 | 1345 | 1128 | 1345 | 206 |
| 1347 | 421 | 1347 | 1220 | 1347 | 187 |
| 1349 | 502 | 1349 | 1314 | 1349 | 200 |
| 1351 | 498 | 1351 | 1337 | 1351 | 246 |
| 1353 | 449 | 1353 | 1325 | 1353 | 209 |
| 1355 | 510 | 1355 | 1304 | 1355 | 216 |
| 1357 | 468 | 1357 | 1318 | 1357 | 217 |
| 1359 | 567 | 1359 | 1300 | 1359 | 198 |
| 1361 | 601 | 1361 | 1330 | 1361 | 202 |
| 1363 | 492 | 1363 | 1258 | 1363 | 249 |
| 1365 | 559 | 1365 | 1261 | 1365 | 219 |
| 1367 | 624 | 1367 | 1305 | 1367 | 261 |
| 1369 | 524 | 1369 | 1214 | 1369 | 247 |
| 1371 | 578 | 1371 | 1292 | 1371 | 247 |
| 1373 | 591 | 1373 | 1329 | 1373 | 278 |
| 1375 | 628 | 1375 | 1325 | 1375 | 281 |
| 1377 | 507 | 1377 | 1292 | 1377 | 252 |
| 1379 | 520 | 1379 | 1258 | 1379 | 286 |
| 1381 | 477 | 1381 | 1272 | 1381 | 290 |
| 1383 | 502 | 1383 | 1225 | 1383 | 267 |
| 1385 | 555 | 1385 | 1293 | 1385 | 265 |
| 1387 | 579 | 1387 | 1244 | 1387 | 254 |
| 1389 | 573 | 1389 | 1249 | 1389 | 332 |
| 1391 | 551 | 1391 | 1195 | 1391 | 303 |
| 1393 | 499 | 1393 | 1317 | 1393 | 280 |
| 1395 | 589 | 1395 | 1256 | 1395 | 341 |
| 1397 | 539 | 1397 | 1148 | 1397 | 292 |
| 1399 | 574 | 1399 | 1270 | 1399 | 302 |
| 1401 | 426 | 1401 | 1234 | 1401 | 345 |
| 1403 | 511 | 1403 | 1200 | 1403 | 319 |
| 1405 | 542 | 1405 | 1201 | 1405 | 325 |
| 1407 | 509 | 1407 | 1241 | 1407 | 315 |

| | | | | | |
|---|---|---|---|---|---|
| 1409 | 475 | 1409 | 1203 | 1409 | 310 |
| 1411 | 516 | 1411 | 1218 | 1411 | 363 |
| 1413 | 551 | 1413 | 1129 | 1413 | 332 |
| 1415 | 495 | 1415 | 1134 | 1415 | 322 |
| 1417 | 515 | 1417 | 1169 | 1417 | 323 |
| 1419 | 426 | 1419 | 1101 | 1419 | 307 |
| 1421 | 551 | 1421 | 1125 | 1421 | 290 |
| 1423 | 525 | 1423 | 1145 | 1423 | 337 |
| 1425 | 569 | 1425 | 1068 | 1425 | 261 |
| 1427 | 432 | 1427 | 1107 | 1427 | 323 |
| 1429 | 535 | 1429 | 1115 | 1429 | 350 |
| 1431 | 537 | 1431 | 1015 | 1431 | 348 |
| 1433 | 579 | 1433 | 1081 | 1433 | 311 |
| 1435 | 476 | 1435 | 1036 | 1435 | 329 |
| 1437 | 525 | 1437 | 1024 | 1437 | 316 |
| 1439 | 473 | 1439 | 1038 | 1439 | 343 |
| 1441 | 470 | 1441 | 1006 | 1441 | 309 |
| 1443 | 514 | 1443 | 1077 | 1443 | 278 |
| 1445 | 501 | 1445 | 1120 | 1445 | 330 |
| 1446 | 575 | 1446 | 1251 | 1446 | 339 |
| 1448 | 541 | 1448 | 1394 | 1448 | 312 |
| 1450 | 436 | 1450 | 1595 | 1450 | 340 |
| 1452 | 505 | 1452 | 1829 | 1452 | 310 |
| 1454 | 464 | 1454 | 2141 | 1454 | 341 |
| 1456 | 560 | 1456 | 2351 | 1456 | 372 |
| 1458 | 636 | 1458 | 2589 | 1458 | 377 |
| 1460 | 552 | 1460 | 2813 | 1460 | 395 |
| 1462 | 608 | 1462 | 2948 | 1462 | 398 |
| 1464 | 629 | 1464 | 2996 | 1464 | 371 |
| 1466 | 553 | 1466 | 2991 | 1466 | 371 |
| 1468 | 584 | 1468 | 2877 | 1468 | 392 |
| 1470 | 580 | 1470 | 2727 | 1470 | 350 |
| 1472 | 574 | 1472 | 2501 | 1472 | 412 |

| | | | | | |
|---|---|---|---|---|---|
| 1474 | 497 | 1474 | 2226 | 1474 | 401 |
| 1476 | 555 | 1476 | 1925 | 1476 | 397 |
| 1478 | 546 | 1478 | 1710 | 1478 | 406 |
| 1480 | 508 | 1480 | 1528 | 1480 | 413 |
| 1482 | 605 | 1482 | 1330 | 1482 | 443 |
| 1484 | 611 | 1484 | 1173 | 1484 | 461 |
| 1486 | 646 | 1486 | 1016 | 1486 | 459 |
| 1488 | 558 | 1488 | 929 | 1488 | 381 |
| 1490 | 684 | 1490 | 842 | 1490 | 478 |
| 1492 | 654 | 1492 | 918 | 1492 | 444 |
| 1494 | 636 | 1494 | 926 | 1494 | 470 |
| 1496 | 618 | 1496 | 818 | 1496 | 464 |
| 1498 | 752 | 1498 | 839 | 1498 | 467 |
| 1500 | 749 | 1500 | 915 | 1500 | 530 |
| 1502 | 728 | 1502 | 817 | 1502 | 517 |
| 1504 | 738 | 1504 | 841 | 1504 | 523 |
| 1506 | 654 | 1506 | 829 | 1506 | 538 |
| 1507 | 804 | 1507 | 840 | 1507 | 551 |
| 1509 | 700 | 1509 | 832 | 1509 | 583 |
| 1511 | 830 | 1511 | 826 | 1511 | 536 |
| 1513 | 788 | 1513 | 893 | 1513 | 612 |
| 1515 | 812 | 1515 | 830 | 1515 | 660 |
| 1517 | 778 | 1517 | 885 | 1517 | 629 |
| 1519 | 858 | 1519 | 973 | 1519 | 632 |
| 1521 | 865 | 1521 | 968 | 1521 | 666 |
| 1523 | 859 | 1523 | 951 | 1523 | 701 |
| 1525 | 916 | 1525 | 987 | 1525 | 704 |
| 1527 | 907 | 1527 | 956 | 1527 | 760 |
| 1529 | 825 | 1529 | 1040 | 1529 | 755 |
| 1531 | 984 | 1531 | 976 | 1531 | 784 |
| 1533 | 970 | 1533 | 1078 | 1533 | 746 |
| 1535 | 1031 | 1535 | 1062 | 1535 | 784 |
| 1537 | 983 | 1537 | 1058 | 1537 | 811 |

| | | | | | |
|---|---|---|---|---|---|
| 1539 | 1050 | 1539 | 1122 | 1539 | 857 |
| 1541 | 958 | 1541 | 1021 | 1541 | 839 |
| 1543 | 1111 | 1543 | 1172 | 1543 | 870 |
| 1545 | 1140 | 1545 | 1100 | 1545 | 926 |
| 1547 | 1146 | 1547 | 1182 | 1547 | 965 |
| 1549 | 1148 | 1549 | 1203 | 1549 | 943 |
| 1551 | 1131 | 1551 | 1199 | 1551 | 1037 |
| 1552 | 1168 | 1552 | 1270 | 1552 | 998 |
| 1554 | 1163 | 1554 | 1288 | 1554 | 1077 |
| 1556 | 1334 | 1556 | 1299 | 1556 | 1077 |
| 1558 | 1292 | 1558 | 1325 | 1558 | 1122 |
| 1560 | 1428 | 1560 | 1323 | 1560 | 1117 |
| 1562 | 1329 | 1562 | 1412 | 1562 | 1138 |
| 1564 | 1320 | 1564 | 1492 | 1564 | 1183 |
| 1566 | 1369 | 1566 | 1437 | 1566 | 1297 |
| 1568 | 1505 | 1568 | 1527 | 1568 | 1308 |
| 1570 | 1500 | 1570 | 1499 | 1570 | 1324 |
| 1572 | 1527 | 1572 | 1575 | 1572 | 1418 |
| 1574 | 1587 | 1574 | 1650 | 1574 | 1417 |
| 1576 | 1697 | 1576 | 1712 | 1576 | 1499 |
| 1578 | 1799 | 1578 | 1718 | 1578 | 1504 |
| 1580 | 1773 | 1580 | 1735 | 1580 | 1587 |
| 1582 | 1819 | 1582 | 1868 | 1582 | 1617 |
| 1584 | 1908 | 1584 | 1916 | 1584 | 1697 |
| 1586 | 1970 | 1586 | 1964 | 1586 | 1756 |
| 1588 | 2001 | 1588 | 1986 | 1588 | 1814 |
| 1589 | 2049 | 1589 | 2063 | 1589 | 1846 |
| 1591 | 2150 | 1591 | 2252 | 1591 | 1911 |
| 1593 | 2219 | 1593 | 2227 | 1593 | 1964 |
| 1595 | 2304 | 1595 | 2312 | 1595 | 2108 |
| 1597 | 2458 | 1597 | 2391 | 1597 | 2160 |
| 1599 | 2454 | 1599 | 2546 | 1599 | 2227 |
| 1601 | 2560 | 1601 | 2561 | 1601 | 2310 |

| | | | | | |
|---|---|---|---|---|---|
| 1603 | 2546 | 1603 | 2592 | 1603 | 2361 |
| 1605 | 2671 | 1605 | 2707 | 1605 | 2428 |
| 1607 | 2674 | 1607 | 2864 | 1607 | 2485 |
| 1609 | 2768 | 1609 | 2899 | 1609 | 2557 |
| 1611 | 2897 | 1611 | 2912 | 1611 | 2616 |
| 1613 | 2931 | 1613 | 3024 | 1613 | 2684 |
| 1615 | 3036 | 1615 | 3058 | 1615 | 2811 |
| 1617 | 3102 | 1617 | 3175 | 1617 | 2876 |
| 1619 | 3140 | 1619 | 3208 | 1619 | 2901 |
| 1620 | 3238 | 1620 | 3233 | 1620 | 2910 |
| 1622 | 3285 | 1622 | 3322 | 1622 | 3033 |
| 1624 | 3287 | 1624 | 3316 | 1624 | 3080 |
| 1626 | 3360 | 1626 | 3317 | 1626 | 3129 |
| 1628 | 3486 | 1628 | 3333 | 1628 | 3196 |
| 1630 | 3431 | 1630 | 3445 | 1630 | 3208 |
| 1632 | 3485 | 1632 | 3444 | 1632 | 3205 |
| 1634 | 3511 | 1634 | 3451 | 1634 | 3232 |
| 1636 | 3544 | 1636 | 3509 | 1636 | 3214 |
| 1638 | 3374 | 1638 | 3420 | 1638 | 3210 |
| 1640 | 3545 | 1640 | 3440 | 1640 | 3168 |
| 1642 | 3509 | 1642 | 3399 | 1642 | 3218 |
| 1644 | 3392 | 1644 | 3342 | 1644 | 3209 |
| 1646 | 3409 | 1646 | 3328 | 1646 | 3135 |
| 1648 | 3449 | 1648 | 3269 | 1648 | 3099 |
| 1649 | 3320 | 1649 | 3267 | 1649 | 3025 |
| 1651 | 3310 | 1651 | 3251 | 1651 | 3024 |
| 1653 | 3275 | 1653 | 3160 | 1653 | 2998 |
| 1655 | 3154 | 1655 | 3068 | 1655 | 2896 |
| 1657 | 3123 | 1657 | 3076 | 1657 | 2861 |
| 1659 | 2974 | 1659 | 2955 | 1659 | 2835 |
| 1661 | 3018 | 1661 | 2876 | 1661 | 2712 |
| 1663 | 2875 | 1663 | 2792 | 1663 | 2704 |
| 1665 | 2795 | 1665 | 2733 | 1665 | 2613 |

| | | | | | |
|---|---|---|---|---|---|
| 1667 | 2734 | 1667 | 2708 | 1667 | 2577 |
| 1669 | 2736 | 1669 | 2616 | 1669 | 2505 |
| 1671 | 2634 | 1671 | 2578 | 1671 | 2381 |
| 1673 | 2518 | 1673 | 2356 | 1673 | 2378 |
| 1674 | 2446 | 1674 | 2417 | 1674 | 2238 |
| 1676 | 2343 | 1676 | 2295 | 1676 | 2165 |
| 1678 | 2252 | 1678 | 2280 | 1678 | 2080 |
| 1680 | 2257 | 1680 | 2069 | 1680 | 2074 |
| 1682 | 2117 | 1682 | 2015 | 1682 | 1963 |
| 1684 | 2040 | 1684 | 1936 | 1684 | 1893 |
| 1686 | 2016 | 1686 | 1935 | 1686 | 1795 |
| 1688 | 1917 | 1688 | 1825 | 1688 | 1720 |
| 1690 | 1803 | 1690 | 1715 | 1690 | 1679 |
| 1692 | 1708 | 1692 | 1641 | 1692 | 1596 |
| 1694 | 1688 | 1694 | 1575 | 1694 | 1535 |
| 1696 | 1642 | 1696 | 1543 | 1696 | 1457 |
| 1698 | 1522 | 1698 | 1468 | 1698 | 1436 |
| 1699 | 1464 | 1699 | 1337 | 1699 | 1335 |
| 1701 | 1416 | 1701 | 1350 | 1701 | 1297 |
| 1703 | 1344 | 1703 | 1320 | 1703 | 1235 |
| 1705 | 1282 | 1705 | 1205 | 1705 | 1163 |
| 1707 | 1251 | 1707 | 1170 | 1707 | 1093 |
| 1709 | 1269 | 1709 | 1133 | 1709 | 1083 |
| 1711 | 1176 | 1711 | 1091 | 1711 | 1019 |
| 1713 | 1122 | 1713 | 1092 | 1713 | 978 |
| 1715 | 1073 | 1715 | 1075 | 1715 | 912 |
| 1717 | 1041 | 1717 | 940 | 1717 | 877 |
| 1719 | 1016 | 1719 | 889 | 1719 | 814 |
| 1720 | 868 | 1720 | 887 | 1720 | 800 |
| 1722 | 891 | 1722 | 805 | 1722 | 762 |
| 1724 | 909 | 1724 | 745 | 1724 | 675 |
| 1726 | 897 | 1726 | 851 | 1726 | 697 |
| 1728 | 882 | 1728 | 772 | 1728 | 636 |

| | | | | | |
|---|---|---|---|---|---|
| 1730 | 784 | 1730 | 770 | 1730 | 636 |
| 1732 | 723 | 1732 | 742 | 1732 | 624 |
| 1734 | 758 | 1734 | 696 | 1734 | 576 |
| 1736 | 772 | 1736 | 667 | 1736 | 520 |
| 1738 | 698 | 1738 | 657 | 1738 | 554 |
| 1740 | 618 | 1740 | 602 | 1740 | 515 |
| 1742 | 600 | 1742 | 578 | 1742 | 494 |
| 1743 | 605 | 1743 | 635 | 1743 | 439 |
| 1745 | 586 | 1745 | 569 | 1745 | 425 |
| 1747 | 592 | 1747 | 589 | 1747 | 413 |
| 1749 | 597 | 1749 | 494 | 1749 | 404 |
| 1751 | 575 | 1751 | 552 | 1751 | 350 |
| 1753 | 548 | 1753 | 558 | 1753 | 378 |
| 1755 | 604 | 1755 | 401 | 1755 | 352 |
| 1757 | 379 | 1757 | 486 | 1757 | 323 |
| 1759 | 497 | 1759 | 440 | 1759 | 296 |
| 1761 | 453 | 1761 | 458 | 1761 | 288 |
| 1762 | 372 | 1762 | 540 | 1762 | 282 |
| 1764 | 413 | 1764 | 439 | 1764 | 274 |
| 1766 | 354 | 1766 | 373 | 1766 | 256 |
| 1768 | 447 | 1768 | 394 | 1768 | 238 |
| 1770 | 353 | 1770 | 406 | 1770 | 223 |
| 1772 | 322 | 1772 | 364 | 1772 | 212 |
| 1774 | 315 | 1774 | 324 | 1774 | 171 |
| 1776 | 344 | 1776 | 363 | 1776 | 205 |
| 1778 | 258 | 1778 | 339 | 1778 | 179 |
| 1780 | 243 | 1780 | 351 | 1780 | 149 |
| 1781 | 308 | 1781 | 242 | 1781 | 143 |
| 1783 | 297 | 1783 | 235 | 1783 | 165 |
| 1785 | 299 | 1785 | 216 | 1785 | 165 |
| 1787 | 266 | 1787 | 240 | 1787 | 114 |
| 1789 | 161 | 1789 | 258 | 1789 | 133 |
| 1791 | 213 | 1791 | 207 | 1791 | 85 |

| | | | | | |
|---|---|---|---|---|---|
| 1793 | 182 | 1793 | 219 | 1793 | 71 |
| 1795 | 242 | 1795 | 133 | 1795 | 101 |
| 1797 | 138 | 1797 | 107 | 1797 | 49 |
| 1799 | 125 | 1799 | 109 | 1799 | 104 |
| 1800 | 136 | 1800 | 138 | 1800 | 60 |
