# Supplementary material for: Novel diagnostic and therapeutic techniques reveal changed metabolic profiles in recurrent focal segmental glomerulosclerosis
Source: Sci Rep. 2021 Feb 25;11:4577. doi: 10.1038/s41598-021-83883-w (PMC7907124; doi:10.1038/s41598-021-83883-w)
Supplement: Supplementary file 3 — Supplementary Information 3. [file 41598_2021_83883_MOESM3_ESM.pdf]

CTRL serum		FSGS serum	
Raman Shift (cm-1)	Raman intensity (arb. units)	Raman Shift (cm-1)	Raman intensity (arb. units)
351	-10	351	-5
352	3	352	6
354	11	354	23
356	19	356	15
357	58	357	-2
359	24	359	44
360	8	360	1
362	-1	362	-1
363	8	363	-10
365	-16	365	5
367	-2	367	-11
368	0	368	1
370	0	370	-6
371	20	371	3
373	2	373	0
374	-8	374	-30
376	13	376	-33
377	5	377	0
379	-8	379	0
381	18	381	-3
382	-6	382	3
384	17	384	-10
385	24	385	25
387	57	387	27
388	147	388	91
390	143	390	184
391	163	391	224
393	179	393	267
395	233	395	299
396	255	396	391
398	317	398	440
399	384	399	530
401	384	401	610
402	426	402	637
404	435	404	672
405	487	405	707
407	480	407	729
409	460	409	726
410	455	410	708
412	461	412	694
413	437	413	709
415	432	415	662
416	391	416	612
418	372	418	575
419	338	419	552
421	323	421	492
423	287	423	464

424	220	424	420
426	216	426	328
427	185	427	311
429	169	429	261
430	128	430	216
432	103	432	161
433	69	433	102
435	51	435	63
436	38	436	25
438	42	438	-6
440	25	440	3
441	-4	441	-1
443	50	443	-26
444	45	444	29
446	54	446	37
447	112	447	54
449	114	449	120
450	134	450	106
452	116	452	142
453	163	453	126
455	164	455	160
457	206	457	129
458	187	458	182
460	198	460	144
461	222	461	162
463	210	463	162
464	189	464	110
466	186	466	91
467	200	467	78
469	183	469	71
470	185	470	45
472	177	472	48
473	209	474	46
475	200	475	54
477	204	477	41
478	226	478	54
480	224	480	74
481	251	481	84
483	310	483	138
484	333	484	207
486	398	486	242
487	439	487	338
489	452	489	389
490	476	490	385
492	504	492	418
493	485	494	431
495	505	495	395
497	489	497	437
498	488	498	410
500	480	500	389

501	477	501	378
503	493	503	360
504	477	504	370
506	495	506	347
507	496	507	357
509	496	509	385
510	560	510	408
512	557	512	487
513	611	513	507
515	636	515	586
516	653	517	650
518	702	518	695
520	723	520	762
521	735	521	788
523	767	523	800
524	790	524	813
526	779	526	833
527	791	527	826
529	793	529	839
530	756	530	831
532	791	532	782
533	796	533	820
535	758	535	812
536	774	536	771
538	745	538	787
539	738	539	755
541	761	541	729
542	736	542	755
544	732	544	718
546	691	546	681
547	664	547	654
549	645	549	619
550	625	550	574
552	588	552	573
553	599	553	534
555	564	555	569
556	536	556	532
558	543	558	484
559	547	559	502
561	493	561	530
562	488	562	454
564	460	564	458
565	480	565	434
567	452	567	469
568	456	568	448
570	477	570	433
571	456	571	456
573	419	573	432
574	406	574	365
576	420	576	380

577	405	577	384
579	366	579	362
581	378	581	309
582	347	582	327
584	348	584	260
585	364	585	292
587	356	587	293
588	342	588	309
590	386	590	309
591	413	591	359
593	384	593	389
594	434	594	367
596	422	596	444
597	402	597	425
599	360	599	396
600	363	600	345
602	334	602	339
603	310	603	338
605	269	605	299
606	254	606	268
608	222	608	199
609	197	609	162
611	196	611	140
612	166	612	156
614	173	614	163
615	234	615	244
617	293	617	446
618	520	618	751
620	713	620	1054
621	661	621	1007
623	436	623	671
624	235	624	371
626	104	626	215
627	55	627	85
629	13	629	38
630	-4	630	10
632	1	632	-10
633	33	633	44
635	45	635	123
636	53	636	201
638	162	638	336
639	345	639	673
641	576	641	1030
642	635	642	1140
644	444	644	922
645	265	646	624
647	163	647	423
648	48	649	340
650	26	650	230
652	23	652	232

653	34	653	239
655	21	655	249
656	48	656	272
658	88	658	293
659	106	659	336
661	141	661	400
662	184	662	471
664	236	664	532
665	268	665	604
667	277	667	663
668	294	668	638
670	308	670	646
671	279	671	654
673	246	673	586
674	231	674	552
676	220	676	549
677	195	677	540
679	151	679	507
680	144	680	480
682	164	682	496
683	136	683	506
685	121	685	464
686	111	686	437
687	134	688	424
689	134	689	459
690	109	691	493
692	105	692	471
693	137	693	513
695	134	695	560
696	166	696	576
698	193	698	624
699	203	699	644
701	169	701	661
702	158	702	623
704	152	704	630
705	196	705	667
707	237	707	770
708	285	708	880
710	358	710	989
711	421	711	1143
713	520	713	1267
714	600	714	1412
716	631	716	1506
717	701	717	1530
719	679	719	1589
720	660	720	1545
722	662	722	1506
723	633	723	1492
725	569	725	1404
726	558	726	1301

728	494	728	1234
729	418	729	1133
731	331	731	1039
732	268	732	941
734	279	734	894
735	240	735	909
737	267	737	907
738	292	738	953
740	307	740	1010
741	362	741	1035
743	377	743	1105
744	395	744	1119
746	399	746	1151
747	415	747	1148
749	434	749	1134
750	445	750	1158
752	472	752	1200
753	549	753	1297
755	625	755	1459
756	708	756	1536
757	720	757	1563
759	675	759	1477
760	619	760	1340
762	564	762	1191
763	511	763	1086
765	452	765	957
766	401	766	854
768	364	768	737
769	373	769	665
771	383	771	672
772	452	772	665
774	523	774	795
775	604	775	896
777	750	777	1014
778	866	778	1241
780	901	780	1333
781	883	781	1333
783	806	783	1250
784	700	784	1108
786	594	786	934
787	466	787	787
789	351	789	598
790	272	790	426
791	119	791	292
793	65	793	127
794	13	794	42
796	-3	796	-4
797	2	797	7
799	-2	799	15
800	7	800	30

802	30	802	87
803	85	803	154
805	66	805	237
806	125	806	243
808	159	808	329
809	165	809	374
811	151	811	390
812	145	812	394
814	137	814	401
815	206	815	416
817	237	817	527
818	308	818	607
819	408	819	748
821	479	821	949
822	596	822	1095
824	730	824	1263
825	771	825	1447
827	819	827	1489
828	809	828	1507
830	778	830	1454
831	750	831	1394
833	668	833	1280
834	568	834	1157
836	543	836	1031
837	494	837	981
839	479	839	922
840	474	840	921
841	513	841	964
843	580	843	1048
844	669	844	1210
846	799	846	1407
847	918	847	1655
849	1097	849	1845
850	1168	850	2069
852	1207	852	2077
853	1154	853	2032
855	1086	855	1891
856	952	856	1715
858	847	858	1499
859	720	859	1353
861	621	861	1189
862	583	862	1053
863	452	863	980
865	439	865	808
866	363	866	780
868	338	868	704
869	381	869	719
871	414	871	755
872	424	872	840
874	423	874	866

875	447	875	883
877	415	877	855
878	384	878	777
879	323	879	700
881	291	881	571
882	211	882	538
884	210	884	440
885	164	885	430
887	144	887	408
888	165	888	406
890	132	890	396
891	142	891	382
893	145	893	359
894	163	894	339
895	148	895	334
897	147	897	299
898	95	898	299
900	87	900	249
901	85	901	207
903	73	903	177
904	54	904	119
906	24	906	62
907	-8	907	22
909	2	909	-19
910	2	910	-4
911	14	911	4
913	39	913	32
914	53	914	94
916	157	916	121
917	244	917	269
919	283	919	401
920	313	920	493
922	361	922	548
923	413	923	598
925	444	925	656
926	484	926	736
927	507	927	805
929	556	929	869
930	593	930	951
932	632	932	1011
933	648	933	1089
935	645	935	1081
936	619	936	1055
938	597	938	992
939	552	939	918
940	483	940	829
942	438	942	740
943	423	943	683
945	379	945	612
946	407	946	575

948	339	948	616
949	342	949	533
951	341	951	562
952	372	952	581
953	385	953	630
955	454	955	670
956	458	956	729
958	444	958	748
959	441	959	714
961	381	961	692
962	357	962	595
964	360	964	560
965	305	965	582
966	279	966	515
968	265	968	499
969	217	969	499
971	198	971	429
972	196	972	396
974	150	974	365
975	110	975	287
976	57	976	200
978	24	978	96
979	22	979	34
981	0	981	0
982	-1	982	0
984	16	984	-4
985	16	985	61
987	68	987	90
988	157	988	172
989	163	989	286
991	167	991	239
992	174	992	259
994	231	994	290
995	311	995	499
997	566	997	885
998	1087	998	1824
999	2130	999	3621
1001	3610	1001	5893
1002	4308	1002	6948
1004	3525	1004	5798
1005	2251	1005	3779
1007	1332	1007	2351
1008	955	1008	1567
1010	841	1009	1249
1011	700	1011	1067
1012	575	1012	828
1014	450	1014	620
1015	315	1015	468
1017	260	1017	326
1018	197	1018	261

1020	187	1020	211
1021	217	1021	267
1022	229	1022	330
1024	328	1024	417
1025	445	1025	651
1027	597	1027	972
1028	793	1028	1323
1030	1011	1030	1624
1031	1114	1031	1777
1032	1018	1032	1699
1034	887	1034	1467
1035	735	1035	1252
1037	631	1037	1052
1038	574	1038	938
1040	528	1040	892
1041	504	1041	857
1042	530	1042	830
1044	494	1044	870
1045	477	1045	842
1047	483	1047	835
1048	469	1048	887
1049	495	1049	874
1051	501	1051	991
1052	515	1052	1000
1054	593	1054	1043
1055	634	1055	1214
1057	629	1057	1326
1058	706	1058	1409
1059	790	1059	1559
1061	783	1061	1741
1062	846	1062	1773
1064	880	1064	1856
1065	860	1065	1872
1067	872	1067	1817
1068	874	1068	1872
1069	899	1069	1879
1071	912	1071	1958
1072	979	1072	2043
1074	1039	1074	2178
1075	1091	1075	2326
1076	1154	1076	2422
1078	1197	1078	2529
1079	1262	1079	2592
1081	1296	1081	2674
1082	1305	1082	2725
1084	1332	1084	2724
1085	1367	1085	2740
1086	1385	1086	2761
1088	1420	1088	2741
1089	1415	1089	2750

1091	1413	1091	2752
1092	1467	1092	2724
1093	1426	1093	2778
1095	1463	1095	2702
1096	1478	1096	2735
1098	1486	1098	2717
1099	1489	1099	2675
1101	1474	1101	2637
1102	1414	1102	2542
1103	1319	1103	2413
1105	1250	1105	2246
1106	1133	1106	2110
1108	1077	1108	1894
1109	996	1109	1790
1110	905	1110	1640
1112	861	1112	1518
1113	808	1113	1461
1115	829	1115	1402
1116	828	1116	1436
1118	853	1118	1503
1119	910	1119	1585
1120	1015	1120	1760
1122	1057	1122	1964
1123	1193	1123	2075
1125	1255	1125	2233
1126	1234	1126	2261
1127	1153	1127	2125
1129	1007	1129	1866
1130	850	1130	1547
1132	648	1132	1220
1133	490	1133	904
1134	301	1134	597
1136	189	1136	350
1137	92	1137	171
1139	37	1139	34
1140	-1	1140	-9
1141	13	1141	-23
1143	17	1143	-8
1144	67	1144	32
1146	137	1146	126
1147	156	1147	257
1148	174	1148	322
1150	261	1150	434
1151	350	1151	580
1153	427	1153	765
1154	506	1154	912
1155	548	1155	993
1157	560	1157	1004
1158	529	1158	986
1160	509	1160	930

1161	460	1161	889
1163	471	1162	836
1164	502	1164	896
1165	566	1165	987
1167	644	1167	1116
1168	726	1168	1296
1170	844	1170	1472
1171	952	1171	1645
1172	1014	1172	1764
1174	1033	1174	1811
1175	1025	1175	1807
1177	988	1177	1758
1178	923	1178	1704
1179	926	1179	1616
1181	937	1181	1614
1182	911	1182	1607
1184	924	1183	1612
1185	948	1185	1626
1186	981	1186	1661
1188	1005	1188	1741
1189	1097	1189	1804
1190	1150	1190	1943
1192	1230	1192	2081
1193	1329	1193	2191
1195	1411	1195	2356
1196	1489	1196	2535
1197	1627	1197	2698
1199	1731	1199	2993
1200	1934	1200	3214
1202	2120	1202	3553
1203	2285	1203	3882
1204	2423	1204	4122
1206	2509	1206	4300
1207	2570	1207	4360
1209	2530	1209	4362
1210	2483	1210	4262
1211	2410	1211	4125
1213	2389	1213	4019
1214	2394	1214	4004
1216	2418	1216	4045
1217	2477	1217	4135
1218	2502	1218	4260
1220	2636	1220	4377
1221	2734	1221	4594
1223	2847	1223	4789
1224	2905	1224	4986
1225	3018	1225	5172
1227	3070	1227	5382
1228	3141	1228	5501
1229	3245	1229	5668

1231	3245	1231	5840
1232	3317	1232	5918
1234	3361	1234	6054
1235	3462	1235	6154
1236	3453	1236	6316
1238	3512	1238	6375
1239	3560	1239	6504
1241	3585	1241	6609
1242	3613	1242	6687
1243	3652	1243	6791
1245	3679	1245	6908
1246	3689	1246	6980
1248	3714	1247	7088
1249	3707	1249	7140
1250	3702	1250	7182
1252	3734	1252	7234
1253	3733	1253	7352
1254	3731	1254	7386
1256	3782	1256	7453
1257	3795	1257	7551
1259	3791	1259	7616
1260	3838	1260	7642
1261	3798	1261	7714
1263	3818	1263	7681
1264	3859	1264	7716
1266	3819	1265	7742
1267	3793	1267	7668
1268	3793	1268	7601
1270	3781	1270	7551
1271	3735	1271	7474
1272	3672	1272	7374
1274	3691	1274	7263
1275	3659	1275	7222
1277	3594	1277	7152
1278	3629	1278	7044
1279	3615	1279	7098
1281	3626	1281	7069
1282	3621	1282	7111
1283	3660	1283	7133
1285	3705	1285	7211
1286	3716	1286	7295
1288	3783	1288	7365
1289	3840	1289	7534
1290	3923	1290	7654
1292	4013	1292	7888
1293	4077	1293	8138
1294	4195	1294	8387
1296	4289	1296	8678
1297	4417	1297	8896
1299	4523	1299	9143

1300	4612	1300	9335
1301	4646	1301	9463
1303	4677	1303	9474
1304	4698	1304	9485
1305	4736	1305	9433
1307	4773	1307	9400
1308	4780	1308	9372
1310	4787	1310	9334
1311	4788	1311	9286
1312	4799	1312	9220
1314	4787	1314	9183
1315	4774	1315	9116
1316	4746	1316	9042
1318	4707	1318	8943
1319	4666	1319	8836
1321	4591	1321	8711
1322	4572	1322	8560
1323	4485	1323	8468
1325	4430	1325	8301
1326	4383	1326	8204
1327	4394	1327	8100
1329	4368	1329	8077
1330	4372	1330	8059
1332	4369	1331	8079
1333	4391	1333	8093
1334	4357	1334	8097
1336	4382	1336	8095
1337	4372	1337	8128
1338	4317	1338	8068
1340	4244	1340	7981
1341	4173	1341	7803
1342	4048	1342	7616
1344	3904	1344	7334
1345	3718	1345	7047
1347	3527	1347	6701
1348	3376	1348	6362
1349	3229	1349	6094
1351	3099	1351	5783
1352	2988	1352	5556
1353	2876	1353	5363
1355	2779	1355	5212
1356	2732	1356	5044
1357	2699	1357	4959
1359	2627	1359	4857
1360	2576	1360	4752
1362	2531	1362	4673
1363	2472	1363	4592
1364	2433	1364	4473
1366	2400	1366	4403
1367	2390	1367	4303

1368	2314	1368	4251
1370	2273	1370	4094
1371	2218	1371	3990
1372	2145	1372	3876
1374	2106	1374	3761
1375	2080	1375	3673
1377	2035	1377	3608
1378	2009	1378	3556
1379	2007	1379	3489
1381	1978	1381	3500
1382	1980	1382	3474
1383	1950	1383	3483
1385	2012	1385	3467
1386	1972	1386	3519
1387	1964	1387	3503
1389	1976	1389	3475
1390	1952	1390	3509
1391	1944	1391	3486
1393	1951	1393	3476
1394	1924	1394	3499
1396	1899	1396	3505
1397	1897	1397	3487
1398	1906	1398	3500
1400	1906	1400	3512
1401	1894	1401	3515
1402	1897	1402	3525
1404	1904	1404	3532
1405	1902	1405	3522
1406	1886	1406	3524
1408	1908	1408	3545
1409	1948	1409	3619
1410	2007	1410	3727
1412	2052	1412	3846
1413	2110	1413	3975
1415	2189	1415	4129
1416	2257	1416	4304
1417	2339	1417	4468
1419	2369	1419	4598
1420	2397	1420	4686
1421	2482	1421	4792
1423	2503	1423	4895
1424	2537	1424	5016
1425	2584	1425	5168
1427	2699	1427	5405
1428	2836	1428	5793
1429	3035	1429	6268
1431	3276	1431	6905
1432	3589	1432	7601
1433	3955	1433	8390
1435	4308	1435	9220

1436	4663	1436	9929
1437	4969	1437	10545
1439	5207	1439	11030
1440	5406	1440	11377
1442	5553	1442	11651
1443	5731	1443	11834
1444	5839	1444	12052
1446	5990	1446	12174
1447	6005	1447	12295
1448	6011	1448	12182
1450	5944	1450	11967
1451	5791	1451	11689
1452	5646	1452	11348
1454	5510	1454	11029
1455	5360	1455	10766
1456	5186	1456	10450
1458	5073	1458	10095
1459	4939	1459	9823
1460	4783	1460	9497
1462	4633	1462	9098
1463	4437	1463	8703
1464	4198	1464	8183
1466	3913	1466	7649
1467	3645	1467	7090
1468	3372	1468	6562
1470	3111	1470	6031
1471	2881	1471	5540
1472	2660	1472	5094
1474	2455	1474	4689
1475	2295	1475	4324
1476	2154	1476	4012
1478	1996	1478	3735
1479	1878	1479	3512
1481	1787	1480	3283
1482	1710	1482	3099
1483	1615	1483	2925
1485	1519	1485	2710
1486	1420	1486	2524
1487	1293	1487	2318
1489	1196	1489	2093
1490	1091	1490	1877
1491	986	1491	1670
1493	878	1493	1465
1494	759	1494	1278
1495	662	1495	1093
1497	591	1497	930
1498	531	1498	812
1499	465	1499	727
1501	440	1501	622
1502	387	1502	570

1503	357	1503	513
1505	335	1505	463
1506	291	1506	440
1507	271	1507	386
1509	252	1509	351
1510	226	1510	319
1511	181	1511	294
1513	168	1513	246
1514	132	1514	210
1515	112	1515	172
1517	107	1517	150
1518	79	1518	142
1519	60	1519	90
1521	60	1521	73
1522	41	1522	66
1523	41	1523	29
1525	36	1525	40
1526	14	1526	30
1527	19	1527	0
1529	24	1529	11
1530	10	1530	10
1531	23	1531	-2
1533	18	1533	5
1534	5	1534	-3
1535	-2	1535	1
1537	17	1537	-2
1538	37	1538	22
1539	43	1539	83
1541	55	1541	104
1542	81	1542	151
1543	122	1543	214
1545	184	1545	300
1546	207	1546	384
1547	239	1547	430
1549	265	1548	483
1550	294	1550	509
1551	337	1551	556
1553	370	1552	588
1554	393	1554	608
1555	392	1555	604
1556	384	1556	596
1558	383	1558	562
1559	350	1559	549
1560	361	1560	535
1562	352	1562	567
1563	391	1563	572
1564	435	1564	650
1566	488	1566	715
1567	524	1567	797
1568	594	1568	844

1570	630	1570	953
1571	702	1571	1000
1572	760	1572	1112
1574	805	1574	1199
1575	825	1575	1253
1576	845	1576	1235
1578	843	1578	1270
1579	826	1579	1257
1580	839	1580	1258
1582	857	1582	1285
1583	890	1583	1326
1584	885	1584	1337
1586	837	1586	1262
1587	765	1587	1107
1588	684	1588	986
1590	657	1590	875
1591	624	1591	851
1592	670	1592	835
1594	698	1593	906
1595	746	1595	963
1596	798	1596	1042
1597	834	1597	1130
1599	906	1599	1253
1600	1015	1600	1451
1601	1148	1601	1687
1603	1278	1603	1924
1604	1324	1604	2091
1605	1359	1605	2119
1607	1299	1607	2066
1608	1271	1608	1996
1609	1242	1609	1985
1611	1259	1611	2010
1612	1282	1612	2094
1613	1319	1613	2157
1615	1361	1615	2213
1616	1368	1616	2247
1617	1341	1617	2229
1619	1310	1618	2150
1620	1272	1620	2095
1621	1237	1621	2032
1622	1209	1622	1981
1624	1182	1624	1939
1625	1185	1625	1947
1626	1201	1626	1967
1628	1236	1628	2007
1629	1291	1629	2108
1630	1363	1630	2212
1632	1416	1632	2350
1633	1476	1633	2475
1634	1576	1634	2631

1636	1682	1636	2836
1637	1775	1637	3040
1638	1906	1638	3281
1640	2023	1639	3556
1641	2175	1641	3854
1642	2315	1642	4186
1643	2493	1643	4550
1645	2682	1645	4985
1646	2915	1646	5468
1647	3141	1647	6024
1649	3357	1649	6592
1650	3597	1650	7107
1651	3839	1651	7642
1653	4028	1653	8124
1654	4182	1654	8467
1655	4304	1655	8653
1657	4395	1656	8767
1658	4416	1658	8729
1659	4400	1659	8571
1660	4312	1660	8334
1662	4245	1662	8006
1663	4109	1663	7717
1664	4016	1664	7360
1666	3916	1666	7112
1667	3817	1667	6896
1668	3753	1668	6629
1670	3671	1670	6439
1671	3519	1671	6206
1672	3414	1672	5921
1673	3301	1673	5710
1675	3205	1675	5443
1676	3102	1676	5237
1677	2980	1677	5030
1679	2859	1679	4814
1680	2735	1680	4601
1681	2656	1681	4392
1683	2551	1683	4243
1684	2438	1684	4041
1685	2350	1685	3831
1687	2245	1686	3638
1688	2126	1688	3469
1689	2018	1689	3236
1690	1913	1690	3038
1692	1794	1692	2834
1693	1652	1693	2624
1694	1535	1694	2396
1696	1414	1696	2187
1697	1297	1697	1978
1698	1203	1698	1790
1700	1080	1699	1625

1701	977	1701	1445
1702	879	1702	1278
1703	792	1703	1137
1705	707	1705	1002
1706	642	1706	893
1707	583	1707	810
1709	529	1709	715
1710	461	1710	643
1711	444	1711	574
1712	405	1712	553
1714	388	1714	500
1715	364	1715	468
1716	334	1716	447
1718	322	1718	419
1719	299	1719	401
1720	299	1720	394
1722	273	1722	374
1723	273	1723	371
1724	258	1724	383
1725	244	1725	349
1727	244	1727	354
1728	235	1728	339
1729	235	1729	362
1731	242	1731	353
1732	238	1732	380
1733	236	1733	388
1734	231	1734	414
1736	221	1736	416
1737	224	1737	421
1738	224	1738	450
1740	216	1740	476
1741	206	1741	472
1742	210	1742	464
1744	179	1743	464
1745	187	1745	464
1746	168	1746	465
1747	162	1747	445
1749	133	1749	423
1750	115	1750	372
1751	90	1751	333
1753	91	1753	282
1754	55	1754	259
1755	65	1755	199
1756	55	1756	172
1758	20	1758	134
1759	27	1759	86
1760	8	1760	57
1762	12	1762	33
1763	6	1763	14
1764	-11	1764	5

1765	4	1765	-8
1767	1	1767	-17
1768	7	1768	-23
1769	9	1769	-9
1771	13	1771	-23
1772	5	1772	-33
1773	25	1773	-15
1774	16	1774	-2
1776	27	1776	-14
1777	8	1777	-4
1778	20	1778	-20
1780	36	1780	-13
1781	29	1781	0
1782	9	1782	6
1783	-2	1783	-6
1785	8	1785	-43
1786	28	1786	-4
1787	9	1787	13
1789	15	1788	-15
1790	22	1790	-5
1791	19	1791	3
1792	9	1792	8
1794	18	1794	0
1795	22	1795	5
1796	22	1796	1
1798	14	1797	-1
1799	18	1799	8
1800	2	1800	1
1801	-1	1801	-1
