# Supplementary material for: Novel diagnostic and therapeutic techniques reveal changed metabolic profiles in recurrent focal segmental glomerulosclerosis
Source: Sci Rep. 2021 Feb 25;11:4577. doi: 10.1038/s41598-021-83883-w (PMC7907124; doi:10.1038/s41598-021-83883-w)
Supplement: Supplementary file 4 — Supplementary Information 4. [file 41598_2021_83883_MOESM4_ESM.pdf]

0-biopsy		FSGS recurrence	
Raman Shift (cm-1)	Raman intensity (arb. units)	Raman Shift (cm-1)	Raman intensity (arb. units)
351	-1	349	-1
352	-3	351	4
354	0	353	10
356	2	354	27
357	26	356	25
359	4	357	8
360	0	359	2
362	0	360	0
363	7	362	0
365	-3	364	2
367	3	365	1
368	-1	367	0
370	-2	368	5
371	6	370	7
373	-3	371	-1
374	-6	373	1
376	4	374	7
377	8	376	0
379	-3	378	-1
381	5	379	2
382	-2	381	5
384	5	382	21
385	16	384	49
387	42	385	101
388	93	387	133
390	96	388	145
391	113	390	158
393	114	392	184
395	150	393	215
396	168	395	247
398	208	396	301
399	254	398	337
401	269	399	372
402	311	401	419
404	344	402	465
405	386	404	495
407	391	406	500
409	384	407	501
410	385	409	499
412	381	410	484
413	352	412	451
415	320	413	420
416	295	415	396
418	277	416	360
419	241	418	322
421	213	420	288
423	186	421	234

424	129	423	192
426	111	424	169
427	94	426	135
429	65	427	101
430	45	429	69
432	19	430	42
433	9	432	21
435	5	433	1
436	-14	435	-3
438	17	437	0
440	17	438	-14
441	8	440	-7
443	43	441	8
444	48	443	13
446	60	444	40
447	98	446	60
449	88	447	68
450	106	449	75
452	94	450	83
453	115	452	92
455	106	454	116
457	154	455	129
458	129	457	122
460	138	458	146
461	160	460	151
463	142	461	148
464	147	463	151
466	147	464	157
467	155	466	156
469	149	467	157
470	166	469	164
472	176	471	184
473	211	472	195
475	215	474	204
477	240	475	223
478	261	477	240
480	273	478	262
481	308	480	307
483	354	481	342
484	375	483	383
486	425	484	434
487	467	486	471
489	488	487	511
490	522	489	548
492	544	491	568
493	542	492	591
495	566	494	606
497	562	495	601
498	551	497	601
500	561	498	610

501	569	500	616
503	569	501	628
504	579	503	661
506	620	504	691
507	633	506	709
509	646	507	743
510	696	509	769
512	706	510	792
513	750	512	819
515	767	514	840
516	801	515	881
518	845	517	916
520	859	518	934
521	867	520	960
523	896	521	992
524	911	523	1001
526	903	524	1013
527	916	526	1025
529	917	527	1011
530	897	529	1019
532	935	530	1032
533	934	532	1018
535	919	533	1019
536	934	535	1015
538	899	536	992
539	873	538	980
541	858	540	956
542	818	541	921
544	785	543	885
546	750	544	852
547	730	546	827
549	715	547	808
550	703	549	778
552	668	550	755
553	669	552	736
555	642	553	702
556	615	555	681
558	612	556	684
559	622	558	658
561	571	559	628
562	575	561	614
564	548	562	605
565	560	564	599
567	541	565	585
568	533	567	574
570	525	568	558
571	498	570	523
573	467	571	504
574	453	573	495
576	457	575	481

577	431	576	447
579	401	578	432
581	406	579	408
582	366	581	391
584	364	582	392
585	356	584	382
587	341	585	364
588	321	587	369
590	343	588	380
591	336	590	368
593	314	591	379
594	344	593	397
596	330	594	384
597	315	596	364
599	293	597	350
600	286	599	341
602	276	600	321
603	250	602	288
605	220	603	251
606	190	605	226
608	183	606	211
609	170	608	209
611	188	609	205
612	188	611	206
614	228	612	231
615	302	614	278
617	409	615	387
618	576	617	584
620	711	618	804
621	713	620	903
623	601	621	789
624	437	623	529
626	244	624	294
627	128	626	132
629	36	627	29
630	-2	629	-1
632	14	630	1
633	31	632	7
635	62	634	10
636	105	635	61
638	241	637	173
639	406	638	357
641	597	640	584
642	675	641	743
644	632	643	747
645	523	644	625
647	412	646	441
648	262	647	292
650	198	649	216
652	165	650	183

653	158	652	178
655	161	653	182
656	169	655	203
658	198	656	223
659	210	658	244
661	237	659	265
662	251	661	286
664	273	662	317
665	305	664	332
667	296	665	339
668	310	667	365
670	338	668	370
671	318	670	354
673	309	671	355
674	321	673	362
676	319	674	349
677	302	676	330
679	291	677	325
680	295	679	332
682	312	680	329
683	295	682	308
685	282	683	301
686	283	685	309
687	304	686	329
689	324	688	325
690	307	689	310
692	305	691	322
693	322	692	320
695	306	694	316
696	308	695	322
698	305	697	327
699	302	698	310
701	269	700	287
702	251	701	268
704	230	703	266
705	237	704	270
707	227	705	264
708	220	707	272
710	235	708	281
711	234	710	307
713	270	711	359
714	312	713	399
716	340	714	452
717	411	716	492
719	445	717	514
720	497	719	550
722	561	720	595
723	616	722	632
725	631	723	679
726	665	725	712

728	643	726	702
729	615	728	665
731	562	729	624
732	546	731	618
734	548	732	607
735	521	734	612
737	537	735	634
738	547	737	658
740	555	738	710
741	608	740	771
743	624	741	836
744	683	743	898
746	695	744	946
747	734	746	992
749	764	747	1017
750	797	749	1037
752	842	750	1075
753	924	752	1134
755	988	753	1219
756	1070	755	1309
757	1093	756	1356
759	1085	758	1335
760	1022	759	1262
762	958	761	1165
763	875	762	1068
765	804	763	967
766	709	765	866
768	648	766	795
769	613	768	725
771	564	769	675
772	564	771	651
774	560	772	622
775	554	774	633
777	599	775	667
778	615	777	669
780	597	778	652
781	574	780	628
783	532	781	582
784	472	783	523
786	418	784	448
787	342	786	367
789	277	787	304
790	232	789	227
791	143	790	145
793	94	792	94
794	55	793	58
796	41	794	38
797	18	796	11
799	-2	797	0
800	-3	799	3

802	7	800	16
803	27	802	10
805	10	803	24
806	65	805	63
808	95	806	93
809	126	808	116
811	148	809	142
812	182	811	181
814	229	812	248
815	317	814	308
817	365	815	375
818	466	817	486
819	595	818	604
821	705	820	738
822	853	821	912
824	1018	822	1067
825	1113	824	1189
827	1191	825	1283
828	1219	827	1347
830	1237	828	1375
831	1208	830	1344
833	1136	831	1260
834	1035	833	1178
836	989	834	1122
837	940	836	1076
839	921	837	1052
840	918	839	1051
841	945	840	1081
843	1004	842	1136
844	1083	843	1222
846	1198	844	1333
847	1300	846	1496
849	1470	847	1679
850	1560	849	1833
852	1634	850	1940
853	1636	852	1971
855	1581	853	1920
856	1479	855	1816
858	1360	856	1670
859	1218	858	1509
861	1086	859	1372
862	1005	861	1220
863	849	862	1082
865	796	863	975
866	698	865	873
868	658	866	813
869	633	868	787
871	659	869	771
872	648	871	747
874	644	872	752

875	671	874	756
877	656	875	746
878	649	877	723
879	615	878	690
881	616	880	633
882	595	881	584
884	646	882	549
885	691	884	522
887	756	885	526
888	813	887	535
890	808	888	538
891	764	890	505
893	656	891	467
894	585	893	419
896	471	894	366
897	395	896	309
898	301	897	249
900	236	898	201
901	181	900	155
903	132	901	109
904	81	903	63
906	46	904	21
907	9	906	-1
909	18	907	-10
910	12	909	-19
911	18	910	-4
913	50	912	17
914	65	913	90
916	175	914	216
917	274	916	315
919	330	917	383
920	369	919	438
922	408	920	498
923	455	922	529
925	471	923	546
926	511	925	562
927	532	926	577
929	580	927	610
930	614	929	658
932	674	930	717
933	706	932	756
935	722	933	778
936	713	935	794
938	705	936	782
939	667	938	734
940	604	939	679
942	559	940	629
943	511	942	581
945	474	943	563
946	485	945	514

948	411	946	454
949	407	948	439
951	408	949	429
952	428	951	444
953	458	952	490
955	521	953	541
956	528	955	555
958	509	956	571
959	507	958	549
961	429	959	498
962	385	961	482
964	367	962	430
965	284	964	354
966	233	965	300
968	197	966	235
969	124	968	176
971	95	969	157
972	88	971	139
974	63	972	101
975	35	974	54
977	-1	975	25
978	-5	977	10
979	-1	978	1
981	9	979	-8
982	18	981	-4
984	51	982	-6
985	61	984	0
987	105	985	57
988	190	987	103
989	199	988	143
991	247	989	194
992	299	991	254
994	415	992	338
995	602	994	503
997	985	995	824
998	1642	997	1383
999	2566	998	2357
1001	3595	999	3792
1002	4243	1001	5101
1004	4162	1002	5359
1005	3509	1004	4359
1007	2583	1005	2968
1008	1855	1007	1955
1010	1408	1008	1361
1011	1093	1010	1034
1012	910	1011	856
1014	776	1012	706
1015	629	1014	571
1017	542	1015	455
1018	445	1017	354

1020	409	1018	310
1021	424	1020	286
1022	441	1021	308
1024	547	1022	391
1025	695	1024	507
1027	875	1025	695
1028	1105	1027	981
1030	1348	1028	1304
1031	1512	1030	1527
1032	1531	1031	1586
1034	1470	1032	1489
1035	1333	1034	1315
1037	1212	1035	1151
1038	1114	1037	1013
1040	1031	1038	910
1041	975	1040	859
1042	967	1041	824
1044	933	1042	777
1045	909	1044	751
1047	916	1045	724
1048	905	1047	716
1050	970	1048	710
1051	1004	1049	692
1052	1085	1051	736
1054	1316	1052	830
1055	1619	1054	917
1057	2028	1055	1068
1058	2643	1057	1331
1059	3321	1058	1619
1061	3758	1059	1923
1062	3952	1061	2162
1064	3749	1062	2174
1065	3214	1064	1991
1067	2644	1065	1734
1068	2132	1067	1488
1069	1760	1068	1306
1071	1536	1069	1221
1072	1457	1071	1209
1074	1440	1072	1226
1075	1453	1074	1269
1077	1490	1075	1323
1078	1515	1076	1384
1079	1557	1078	1443
1081	1568	1079	1466
1082	1546	1081	1482
1084	1543	1082	1497
1085	1519	1084	1488
1086	1485	1085	1474
1088	1464	1086	1462
1089	1437	1088	1431

1091	1391	1089	1431
1092	1413	1091	1423
1094	1368	1092	1423
1095	1395	1093	1451
1096	1407	1095	1461
1098	1409	1096	1484
1099	1446	1098	1515
1101	1466	1099	1517
1102	1448	1101	1493
1103	1413	1102	1462
1105	1381	1103	1404
1106	1300	1105	1348
1108	1259	1106	1280
1109	1163	1108	1189
1110	1077	1109	1109
1112	1019	1110	1037
1113	955	1112	991
1115	946	1113	974
1116	959	1115	975
1118	1005	1116	1011
1119	1115	1118	1108
1120	1288	1119	1215
1122	1455	1120	1375
1123	1752	1122	1588
1125	2081	1123	1773
1126	2438	1125	1937
1127	2870	1126	2075
1129	3315	1127	2192
1130	3684	1129	2252
1132	3767	1130	2206
1133	3477	1132	1992
1134	2842	1133	1608
1136	2084	1134	1146
1137	1355	1136	714
1139	788	1137	391
1140	401	1139	173
1141	165	1140	38
1143	27	1141	-15
1144	1	1143	1
1146	21	1144	8
1147	9	1146	11
1149	28	1147	61
1150	105	1148	159
1151	220	1150	269
1153	311	1151	370
1154	393	1153	480
1156	470	1154	574
1157	508	1155	619
1158	512	1157	626
1160	503	1158	598

1161	486	1160	573
1163	505	1161	570
1164	559	1163	590
1165	662	1164	634
1167	789	1165	710
1168	961	1167	840
1170	1129	1168	993
1171	1245	1170	1132
1172	1271	1171	1208
1174	1196	1172	1206
1175	1083	1174	1147
1177	942	1175	1038
1178	791	1177	945
1179	714	1178	887
1181	653	1179	835
1182	595	1181	792
1184	562	1182	766
1185	546	1183	761
1186	560	1185	762
1188	556	1186	790
1189	623	1188	853
1191	676	1189	912
1192	739	1190	991
1193	829	1192	1098
1195	937	1193	1195
1196	1024	1195	1322
1197	1188	1196	1461
1199	1310	1197	1629
1200	1512	1199	1851
1202	1718	1200	2077
1203	1905	1202	2306
1204	2066	1203	2506
1206	2170	1204	2668
1207	2253	1206	2769
1209	2250	1207	2774
1210	2192	1209	2719
1211	2126	1210	2661
1213	2090	1211	2615
1214	2070	1213	2585
1216	2078	1214	2589
1217	2114	1216	2605
1218	2147	1217	2673
1220	2259	1218	2790
1221	2372	1220	2914
1223	2489	1221	3046
1224	2603	1223	3197
1225	2745	1224	3343
1227	2841	1225	3471
1228	2948	1227	3629
1230	3088	1228	3756

1231	3145	1229	3862
1232	3242	1231	3979
1234	3319	1232	4100
1235	3428	1234	4180
1236	3439	1235	4236
1238	3501	1236	4317
1239	3556	1238	4371
1241	3580	1239	4407
1242	3609	1241	4446
1243	3642	1242	4473
1245	3649	1243	4485
1246	3666	1245	4495
1248	3669	1246	4486
1249	3651	1247	4464
1250	3642	1249	4470
1252	3670	1250	4471
1253	3655	1252	4448
1254	3644	1253	4451
1256	3676	1254	4467
1257	3691	1256	4465
1259	3694	1257	4470
1260	3725	1259	4472
1261	3710	1260	4476
1263	3753	1261	4519
1264	3802	1263	4529
1266	3786	1264	4511
1267	3782	1265	4520
1268	3807	1267	4529
1270	3805	1268	4519
1271	3794	1270	4487
1272	3771	1271	4472
1274	3793	1272	4467
1275	3795	1274	4422
1277	3751	1275	4401
1278	3805	1277	4414
1279	3824	1278	4410
1281	3861	1279	4407
1282	3899	1281	4416
1283	3978	1282	4447
1285	4083	1283	4473
1286	4224	1285	4525
1288	4509	1286	4613
1289	4964	1288	4761
1290	5747	1289	5028
1292	6803	1290	5412
1293	7909	1292	5943
1294	8699	1293	6508
1296	8790	1294	6856
1297	8288	1296	6826
1299	7470	1297	6538

1300	6683	1299	6227
1301	6089	1300	6005
1303	5737	1301	5891
1304	5549	1303	5859
1305	5464	1304	5865
1307	5418	1305	5871
1308	5365	1307	5876
1310	5337	1308	5873
1311	5291	1310	5878
1312	5274	1311	5868
1314	5224	1312	5837
1315	5167	1314	5798
1316	5112	1315	5756
1318	5058	1316	5692
1319	4976	1318	5610
1321	4891	1319	5539
1322	4846	1321	5462
1323	4754	1322	5362
1325	4682	1323	5281
1326	4629	1325	5236
1327	4620	1326	5209
1329	4604	1327	5191
1330	4608	1329	5197
1332	4635	1330	5214
1333	4658	1331	5227
1334	4669	1333	5265
1336	4723	1334	5308
1337	4734	1336	5317
1338	4718	1337	5308
1340	4679	1338	5291
1341	4632	1340	5243
1342	4520	1341	5135
1344	4374	1342	4985
1345	4190	1344	4792
1347	3980	1345	4589
1348	3790	1347	4369
1349	3568	1348	4144
1351	3386	1349	3954
1352	3232	1351	3775
1353	3084	1352	3597
1355	2940	1353	3459
1356	2862	1355	3359
1358	2790	1356	3263
1359	2719	1357	3179
1360	2656	1359	3112
1362	2622	1360	3058
1363	2586	1362	3006
1364	2565	1363	2972
1366	2563	1364	2962
1367	2574	1366	2929

1368	2518	1367	2891
1370	2472	1368	2852
1371	2397	1370	2769
1372	2272	1371	2677
1374	2186	1372	2613
1375	2119	1374	2547
1377	2044	1375	2477
1378	1986	1377	2432
1379	1957	1378	2398
1381	1920	1379	2373
1382	1906	1381	2349
1383	1864	1382	2350
1385	1887	1383	2359
1386	1861	1385	2342
1387	1835	1386	2353
1389	1849	1387	2372
1390	1839	1389	2373
1392	1828	1390	2379
1393	1836	1391	2381
1394	1827	1393	2364
1396	1809	1394	2352
1397	1815	1396	2360
1398	1839	1397	2371
1400	1856	1398	2366
1401	1864	1400	2362
1402	1889	1401	2357
1404	1908	1402	2351
1405	1935	1404	2335
1406	1957	1405	2332
1408	2019	1406	2360
1409	2117	1408	2402
1411	2238	1409	2446
1412	2394	1410	2509
1413	2615	1412	2605
1415	2886	1413	2728
1416	3157	1414	2870
1417	3387	1416	3011
1419	3525	1417	3122
1420	3583	1419	3225
1421	3642	1420	3311
1423	3680	1421	3385
1424	3764	1423	3476
1425	3910	1424	3610
1427	4175	1425	3797
1428	4519	1427	4049
1429	5005	1428	4384
1431	5611	1429	4817
1432	6397	1431	5382
1433	7353	1432	6036
1435	8373	1433	6759

1436	9419	1435	7514
1438	10329	1436	8201
1439	10927	1437	8750
1440	11204	1439	9108
1442	11163	1440	9305
1443	11012	1441	9385
1444	10764	1443	9423
1446	10605	1444	9455
1447	10432	1446	9480
1448	10325	1447	9508
1450	10231	1448	9473
1451	10120	1450	9379
1452	10036	1451	9252
1454	9977	1452	9086
1455	9887	1454	8891
1456	9777	1455	8731
1458	9724	1456	8603
1459	9606	1458	8435
1460	9408	1459	8264
1462	9178	1460	8065
1463	8820	1462	7789
1464	8363	1463	7432
1466	7818	1464	6999
1467	7223	1466	6502
1468	6566	1467	5964
1470	5923	1468	5423
1471	5299	1470	4896
1472	4716	1471	4388
1474	4171	1472	3917
1475	3703	1474	3487
1477	3279	1475	3097
1478	2922	1476	2760
1479	2607	1478	2468
1481	2335	1479	2230
1482	2123	1480	2028
1483	1913	1482	1848
1485	1744	1483	1687
1486	1575	1484	1525
1487	1402	1486	1372
1489	1261	1487	1236
1490	1119	1488	1104
1491	988	1490	978
1493	866	1491	849
1494	741	1493	718
1495	622	1494	613
1497	544	1495	532
1498	475	1497	447
1499	392	1498	380
1501	352	1499	325
1502	297	1501	266

1503	258	1502	226
1505	230	1503	186
1506	190	1505	149
1507	161	1506	126
1509	142	1507	105
1510	119	1509	78
1511	91	1510	56
1513	74	1511	33
1514	46	1513	12
1515	36	1514	13
1517	39	1515	5
1518	13	1517	-11
1519	6	1518	-10
1521	9	1519	-10
1522	-1	1521	-7
1523	7	1522	1
1525	2	1523	-3
1526	-7	1525	7
1527	7	1526	23
1529	13	1527	41
1530	22	1529	58
1531	35	1530	80
1533	51	1531	92
1534	53	1533	98
1535	63	1534	118
1537	89	1535	150
1538	123	1537	182
1539	150	1538	210
1541	183	1539	248
1542	229	1540	303
1543	293	1542	373
1545	365	1543	440
1546	418	1544	500
1547	470	1546	566
1549	525	1547	638
1550	581	1548	706
1551	629	1550	778
1553	682	1551	834
1554	693	1552	871
1555	701	1554	900
1557	691	1555	910
1558	677	1556	887
1559	637	1558	854
1561	624	1559	830
1562	606	1560	820
1563	619	1562	831
1564	636	1563	851
1566	665	1564	862
1567	675	1566	891
1568	728	1567	932

1570	757	1568	980
1571	820	1570	1044
1572	876	1571	1101
1574	920	1572	1135
1575	938	1574	1181
1576	986	1575	1229
1578	1016	1576	1260
1579	1041	1578	1298
1580	1088	1579	1348
1582	1132	1580	1405
1583	1182	1582	1471
1584	1205	1583	1519
1586	1195	1584	1540
1587	1170	1586	1502
1588	1109	1587	1428
1590	1073	1588	1349
1591	1036	1589	1306
1592	1056	1591	1311
1594	1098	1592	1342
1595	1149	1593	1398
1596	1227	1595	1468
1598	1314	1596	1556
1599	1429	1597	1667
1600	1569	1599	1811
1601	1731	1600	1991
1603	1901	1601	2178
1604	2013	1603	2333
1605	2088	1604	2420
1607	2090	1605	2432
1608	2080	1607	2395
1609	2057	1608	2337
1611	2043	1609	2290
1612	2043	1611	2289
1613	2074	1612	2339
1615	2128	1613	2402
1616	2154	1615	2436
1617	2148	1616	2449
1619	2132	1617	2444
1620	2102	1618	2419
1621	2064	1620	2379
1623	2029	1621	2346
1624	2004	1622	2324
1625	1998	1624	2314
1626	2002	1625	2338
1628	2053	1626	2397
1629	2121	1628	2462
1630	2192	1629	2528
1632	2261	1630	2596
1633	2344	1632	2689
1634	2454	1633	2791

1636	2564	1634	2896
1637	2679	1636	3006
1638	2798	1637	3121
1640	2929	1638	3250
1641	3085	1639	3379
1642	3221	1641	3514
1643	3392	1642	3671
1645	3570	1643	3855
1646	3778	1645	4051
1647	3986	1646	4246
1649	4177	1647	4447
1650	4380	1649	4676
1651	4593	1650	4899
1653	4765	1651	5082
1654	4893	1653	5265
1655	5044	1654	5436
1657	5142	1655	5560
1658	5209	1656	5642
1659	5242	1658	5674
1660	5231	1659	5685
1662	5245	1660	5654
1663	5181	1662	5599
1664	5165	1663	5565
1666	5130	1664	5503
1667	5069	1666	5445
1668	5031	1667	5403
1670	4984	1668	5331
1671	4881	1669	5247
1672	4799	1671	5159
1674	4684	1672	5056
1675	4580	1673	4951
1676	4462	1675	4821
1677	4321	1676	4673
1679	4178	1677	4525
1680	4030	1679	4390
1681	3914	1680	4257
1683	3769	1681	4108
1684	3620	1683	3964
1685	3489	1684	3831
1687	3351	1685	3680
1688	3193	1686	3526
1689	3042	1688	3368
1690	2889	1689	3208
1692	2731	1690	3031
1693	2554	1692	2841
1694	2377	1693	2656
1696	2209	1694	2471
1697	2043	1696	2296
1698	1893	1697	2112
1700	1719	1698	1918

1701	1559	1699	1735
1702	1408	1701	1558
1703	1266	1702	1397
1705	1140	1703	1253
1706	1035	1705	1118
1707	928	1706	993
1709	833	1707	872
1710	740	1709	785
1711	691	1710	709
1713	622	1711	630
1714	567	1712	574
1715	531	1714	521
1716	480	1715	470
1718	448	1716	435
1719	421	1718	406
1720	397	1719	377
1722	368	1720	357
1723	356	1721	333
1724	326	1723	308
1725	305	1724	288
1727	286	1725	275
1728	275	1727	261
1729	254	1728	253
1731	252	1729	243
1732	230	1731	235
1733	228	1732	226
1735	209	1733	210
1736	192	1734	198
1737	182	1736	201
1738	188	1737	198
1740	170	1738	180
1741	151	1740	166
1742	145	1741	157
1744	127	1742	150
1745	128	1743	145
1746	118	1745	133
1747	104	1746	118
1749	93	1747	98
1750	72	1749	79
1751	59	1750	70
1753	59	1751	59
1754	41	1752	50
1755	47	1754	51
1756	44	1755	31
1758	18	1756	15
1759	17	1758	10
1760	8	1759	5
1762	10	1760	5
1763	6	1761	-3
1764	-6	1763	-3

1765	3	1764	2
1767	3	1765	3
1768	-1	1767	3
1769	2	1768	7
1771	2	1769	9
1772	3	1770	15
1773	7	1772	14
1774	3	1773	19
1776	9	1774	17
1777	1	1776	18
1778	7	1777	29
1780	17	1778	34
1781	15	1779	23
1782	0	1781	5
1783	-12	1782	8
1785	3	1783	24
1786	14	1785	24
1787	3	1786	17
1789	4	1787	21
1790	11	1788	27
1791	15	1790	20
1792	4	1791	17
1794	9	1792	20
1795	7	1794	13
1796	3	1795	10
1798	3	1796	11
1799	7	1797	4
1800	-1	1799	0
1801	0	1800	0
