# Supplementary material for: Novel diagnostic and therapeutic techniques reveal changed metabolic profiles in recurrent focal segmental glomerulosclerosis
Source: Sci Rep. 2021 Feb 25;11:4577. doi: 10.1038/s41598-021-83883-w (PMC7907124; doi:10.1038/s41598-021-83883-w)
Supplement: Supplementary file 5 — Supplementary Information 5. [file 41598_2021_83883_MOESM5_ESM.pdf]

all values are given as Log2 (patient / control sample)

Analyte	Metabolite class	before 1. CytoSorb	after 1. CytoSorb	before CytoSorb at time of remission	after CytoSorb at time of remission	Difference FSGS recurrence-FSGS remission	p-Value (paired, 2-sided)	minus log 10 p-Value (paired, 2-sided)
C0	acylcarnitines	-1,76	-1,69	-0,45	-0,34	1,33	0,009	2,070
PC ae C44:5	glycerophospl	-0,21	-0,25	-0,59	-0,64	-0,38	0,009	2,043
PC aa C40:5	glycerophospl	0,33	0,34	0,17	0,18	-0,16	0,011	1,975
PC ae C34:0	glycerophospl	0,26	0,28	-0,01	0,01	-0,27	0,011	1,963
PC aa C36:1	glycerophospl	-0,19	-0,20	0,18	0,19	0,38	0,016	1,801
SM C16:0	sphingolipids	-0,08	-0,16	0,07	-0,01	0,15	0,016	1,791
PC ae C38:6	glycerophospl	0,19	0,16	0,34	0,31	0,15	0,017	1,777
PC aa C38:0	glycerophospl	-0,32	-0,32	0,08	0,06	0,39	0,017	1,761
PC ae C42:4	glycerophospl	-0,11	-0,19	-0,32	-0,40	-0,21	0,018	1,737
PC aa C40:6	glycerophospl	-0,37	-0,37	-0,14	-0,13	0,24	0,019	1,722
PC ae C40:6	glycerophospl	-0,14	-0,14	-0,08	-0,08	0,06	0,025	1,606
PC ae C38:0	glycerophospl	-0,05	-0,05	-0,16	-0,14	-0,10	0,027	1,575
Pro	aminoacids	0,56	0,64	0,92	0,97	0,35	0,027	1,575
PC aa C32:2	glycerophospl	0,65	0,79	0,15	0,34	-0,47	0,027	1,571
PC aa C34:4	glycerophospl	0,67	0,79	0,00	0,06	-0,70	0,029	1,543
PC ae C30:0	glycerophospl	0,26	0,26	-0,06	-0,03	-0,30	0,029	1,536
PC aa C36:6	glycerophospl	0,44	0,41	-0,03	-0,02	-0,45	0,030	1,517
PC aa C34:3	glycerophospl	0,30	0,38	0,15	0,21	-0,16	0,031	1,510
PC aa C32:1	glycerophospl	0,13	0,34	-0,24	-0,07	-0,39	0,033	1,482
PC aa C40:2	glycerophospl	-0,19	-0,42	-0,26	-0,48	-0,06	0,046	1,334
PC aa C38:4	glycerophospl	0,20	0,26	-0,14	-0,13	-0,36	0,046	1,333
PC ae C36:5	glycerophospl	0,44	0,33	0,63	0,55	0,21	0,048	1,321
PC aa C38:5	glycerophospl	0,23	0,28	-0,02	-0,01	-0,27	0,051	1,296
PC aa C36:2	glycerophospl	-0,06	0,00	0,23	0,25	0,27	0,056	1,253
PC ae C38:4	glycerophospl	0,12	0,08	-0,25	-0,22	-0,34	0,056	1,252
PC aa C32:0	glycerophospl	0,19	0,24	0,27	0,30	0,07	0,057	1,244
PC aa C38:6	glycerophospl	-0,23	-0,19	-0,07	-0,05	0,15	0,059	1,227

PC aa C36:5	glycerophospl	0,33	0,41	0,00	0,00	-0,38	0,064	1,196
PC aa C38:3	glycerophospl	0,00	0,04	-0,21	-0,13	-0,19	0,068	1,167
PC ae C40:4	glycerophospl	-0,15	-0,10	-0,30	-0,28	-0,16	0,071	1,149
Total SM-non	Cust. Met. Ind	-0,11	-0,19	0,01	-0,04	0,13	0,076	1,117
PC ae C34:2	glycerophospl	0,45	0,44	0,53	0,55	0,10	0,079	1,100
Total SM	Cust. Met. Ind	-0,09	-0,17	0,02	-0,02	0,13	0,086	1,066
SM C24:1	sphingolipids	-0,11	-0,17	0,05	0,04	0,19	0,091	1,043
Tyr	aminoacids	-0,19	-0,54	0,13	-0,11	0,37	0,091	1,039
PC aa C36:0	glycerophospl	-0,22	0,07	0,47	0,56	0,59	0,102	0,993
SM C24:0	sphingolipids	-0,22	-0,22	-0,01	0,07	0,26	0,105	0,978
PC aa C28:1	glycerophospl	0,39	0,42	0,48	0,48	0,08	0,106	0,975
PC aa C24:0	glycerophospl	-0,15	0,27	-1,10	-0,41	-0,82	0,107	0,969
SM C26:0	sphingolipids	-0,66	-0,33	0,31	0,34	0,82	0,114	0,943
PC ae C40:3	glycerophospl	-0,08	-0,07	-0,16	-0,12	-0,06	0,116	0,935
PC ae C40:5	glycerophospl	-0,08	-0,14	-0,28	-0,28	-0,16	0,117	0,933
PC aa C42:1	glycerophospl	-0,03	-0,11	-0,11	-0,23	-0,10	0,123	0,911
PC ae C38:3	glycerophospl	0,30	0,24	-0,02	0,03	-0,26	0,130	0,886
PC aa C36:4	glycerophospl	0,19	0,26	0,06	0,06	-0,17	0,132	0,879
SM (OH) C22:	sphingolipids	-0,19	-0,27	0,02	0,05	0,26	0,137	0,862
PC ae C34:3	glycerophospl	0,73	0,57	0,95	0,91	0,28	0,144	0,840
PC ae C36:0	glycerophospl	-0,61	-0,53	-0,20	-0,27	0,33	0,145	0,840
PC aa C34:1	glycerophospl	-0,21	-0,10	0,07	0,07	0,22	0,158	0,800
PC aa C30:0	glycerophospl	0,28	0,44	0,17	0,27	-0,14	0,161	0,792
SM (OH) C24:	sphingolipids	-0,38	-0,53	-0,20	-0,22	0,24	0,164	0,785
C2	acylcarnitines	-0,98	-1,78	-0,25	-0,52	0,99	0,169	0,771
PC aa C40:3	glycerophospl	0,03	0,16	0,25	0,29	0,18	0,171	0,767
PC ae C36:4	glycerophospl	0,29	0,29	0,30	0,30	0,01	0,175	0,757
Orn / Arg	Cust. Met. Ind	1,23	1,47	0,77	0,62	-0,65	0,188	0,725
PC ae C34:1	glycerophospl	-0,03	-0,05	-0,13	-0,10	-0,07	0,188	0,725
PC ae C44:6	glycerophospl	-0,40	-0,44	-0,68	-0,59	-0,21	0,191	0,719
PC aa C32:3	glycerophospl	0,40	0,33	0,09	0,16	-0,24	0,191	0,718
xLeu	aminoacids	0,10	-0,32	0,30	0,11	0,31	0,228	0,642
lysoPC a C20::	glycerophospl	1,04	0,97	0,54	-0,14	-0,80	0,229	0,641

PC ae C42:5	glycerophospl	-0,12	-0,18	-0,31	-0,27	-0,14	0,235	0,628
PC ae C36:2	glycerophospl	0,24	0,29	0,18	0,26	-0,04	0,248	0,605
SM C18:1	sphingolipids	-0,31	-0,36	-0,35	-0,46	-0,07	0,250	0,602
SM C16:1	sphingolipids	-0,07	-0,19	0,02	0,02	0,15	0,257	0,591
lysoPC a C28:1	glycerophospl	0,37	0,39	-0,03	0,23	-0,28	0,259	0,587
lysoPC a C20:4	glycerophospl	0,87	0,82	0,45	-0,30	-0,77	0,273	0,564
SM (OH) C14:1	sphingolipids	0,25	0,11	0,33	0,34	0,16	0,276	0,560
SM (OH) C22:1	sphingolipids	0,12	0,04	0,00	0,01	-0,08	0,290	0,538
PC ae C44:4	glycerophospl	-0,39	-0,23	-0,49	-0,54	-0,21	0,304	0,517
PC aa C34:2	glycerophospl	0,01	0,13	0,29	0,21	0,18	0,307	0,512
Tyr / Phe	Cust. Met. Ind	-0,88	-0,51	-0,13	-0,28	0,49	0,312	0,506
PC ae C38:1	glycerophospl	0,46	0,29	-0,35	-2,41	-1,76	0,315	0,502
Total SM-OH	Cust. Met. Ind	0,04	-0,06	0,08	0,09	0,10	0,315	0,502
PC aa C36:3	glycerophospl	0,05	0,10	0,15	0,13	0,07	0,320	0,495
PC aa C42:2	glycerophospl	-0,22	-0,51	-0,07	0,07	0,37	0,339	0,470
PC ae C36:3	glycerophospl	0,26	0,25	0,28	0,33	0,05	0,345	0,462
SM C18:0	sphingolipids	-0,28	-0,29	-0,16	-0,26	0,08	0,351	0,454
Ser	aminoacids	0,60	-0,07	0,76	0,60	0,42	0,353	0,452
lysoPC a C16:1	glycerophospl	0,27	0,23	0,15	-0,57	-0,46	0,402	0,396
C2 / C0	Cust. Met. Ind	0,52	0,00	0,12	-0,06	-0,23	0,403	0,395
Arg	aminoacids	-0,41	-0,93	-0,24	0,22	0,66	0,403	0,394
C4:1	acylcarnitines	0,00	0,59	-0,10	-0,19	-0,44	0,421	0,376
His	aminoacids	-0,05	-0,20	0,03	0,46	0,37	0,425	0,371
Thr	aminoacids	0,80	-0,28	0,92	0,87	0,63	0,432	0,364
lysoPC a C24:0	glycerophospl	-0,40	-0,22	-0,65	-0,25	-0,13	0,434	0,363
PC ae C40:1	glycerophospl	0,10	0,08	0,20	0,09	0,05	0,440	0,357
PC ae C40:2	glycerophospl	-0,11	0,06	0,08	0,08	0,10	0,440	0,356
Total SM-OH /	Cust. Met. Ind	-0,11	0,23	-0,01	0,24	0,06	0,442	0,354
lysoPC a C18:0	glycerophospl	0,04	-0,08	0,55	-0,04	0,28	0,454	0,343
SM C26:1	sphingolipids	-0,17	-0,44	-0,12	0,34	0,42	0,455	0,342
lysoPC a C26:1	glycerophospl	-0,34	-0,06	-0,96	-0,09	-0,33	0,464	0,334
Met	aminoacids	0,25	-0,34	0,28	0,23	0,30	0,467	0,331
PC ae C38:2	glycerophospl	-0,23	0,15	0,11	0,17	0,18	0,469	0,329

C12:1	acylcarnitines	-0,41	-0,02	-0,44	-0,79	-0,40	0,478	0,321
PC ae C42:1	glycerophospl	-0,30	-0,30	-0,54	-0,30	-0,12	0,496	0,304
PC aa C38:1	glycerophospl	0,06	0,10	1,02	0,06	0,46	0,528	0,277
lysoPC a C26:(	glycerophospl	-0,09	-0,38	-1,36	-0,30	-0,59	0,543	0,265
PC ae C44:3	glycerophospl	0,19	0,00	-0,40	0,08	-0,26	0,581	0,236
lysoPC a C17:(	glycerophospl	0,56	0,39	0,62	-0,01	-0,17	0,599	0,223
PC aa C42:4	glycerophospl	-0,12	-0,37	-0,27	-0,35	-0,06	0,601	0,221
lysoPC a C16:(	glycerophospl	-0,01	-0,14	0,51	-0,23	0,21	0,610	0,215
SM C22:3	sphingolipids	0,62	0,31	0,33	0,36	-0,12	0,615	0,211
Gly	aminoacids	0,57	-0,11	0,45	0,55	0,27	0,615	0,211
C10:2	acylcarnitines	-0,54	0,19	-0,39	-0,62	-0,33	0,619	0,208
PC ae C42:2	glycerophospl	-0,19	-0,10	-0,03	-0,14	0,06	0,651	0,187
PC ae C32:2	glycerophospl	0,03	-0,09	0,00	0,03	0,05	0,654	0,184
PC ae C42:3	glycerophospl	-0,18	-0,33	-0,21	-0,21	0,04	0,669	0,174
PC aa C42:5	glycerophospl	0,00	0,08	0,03	-0,03	-0,04	0,673	0,172
PC aa C42:6	glycerophospl	0,15	0,10	-0,01	0,15	-0,06	0,677	0,170
Trp	aminoacids	-0,25	-0,56	-0,33	-0,34	0,07	0,705	0,152
PC ae C36:1	glycerophospl	0,02	0,12	0,03	0,08	-0,01	0,748	0,126
H1	sugars	-0,35	-0,28	-0,44	-0,04	0,07	0,755	0,122
lysoPC a C18::	glycerophospl	0,95	0,97	1,26	0,28	-0,19	0,766	0,115
PC ae C38:5	glycerophospl	0,07	-0,01	0,04	0,06	0,02	0,784	0,106
SM C20:2	sphingolipids	0,10	0,23	0,14	0,15	-0,02	0,798	0,098
lysoPC a C28:(	glycerophospl	0,22	0,25	-0,36	0,56	-0,13	0,815	0,089
SM (OH) C16:	sphingolipids	-0,02	-0,09	-0,04	-0,06	0,00	0,845	0,073
Orn	aminoacids	1,07	0,43	0,61	0,72	-0,08	0,867	0,062
C14:1	acylcarnitines	-0,39	-0,46	-0,28	-0,62	-0,03	0,877	0,057
C18:2	acylcarnitines	-0,06	0,06	0,39	-0,60	-0,10	0,881	0,055
PC aa C40:4	glycerophospl	-0,02	-0,10	-0,03	-0,08	0,00	0,900	0,046
PC ae C30:2	glycerophospl	0,18	0,13	-0,05	0,32	-0,02	0,944	0,025
Gln	aminoacids	0,13	0,08	-0,06	0,25	-0,01	0,950	0,022
Phe	aminoacids	0,45	0,08	0,20	0,29	-0,02	0,951	0,022
C3-DC (C4-OH	acylcarnitines	-0,85	-0,19	-0,47	-0,62	-0,03	0,952	0,021
PC aa C42:0	glycerophospl	0,00	-0,10	-0,04	-0,05	0,00	0,980	0,009

**lysoPC a C18:**	**glycerophosp**	0,36	0,26	0,76	-0,13	0,00	0,996	0,002
**PC ae C32:1**	**glycerophosp**	-0,14	-0,30	-0,22	-0,22	0,00	0,998	0,001
