# Supplementary material for: Novel diagnostic and therapeutic techniques reveal changed metabolic profiles in recurrent focal segmental glomerulosclerosis
Source: Sci Rep. 2021 Feb 25;11:4577. doi: 10.1038/s41598-021-83883-w (PMC7907124; doi:10.1038/s41598-021-83883-w)
Supplement: Supplementary file 6 — Supplementary Information 6. [file 41598_2021_83883_MOESM6_ESM.pdf]

	0	FSGS patient at time of disease recurrence	10 healthy controls from EPIC study	FSGS patient at time of disease recurrence	10 healthy controls from EPIC study
**Analyte**	**Metabolite class**	**Mean (Concentration in µM, red FSGS> CTRL, green FSGS< CTRL)**		**SD**	
**C0**	acylcarnitines	30,93	32,72	14,40	7,07
**C14:1**	acylcarnitines	0,16	0,09	0,03	0,02
**C18:2**	acylcarnitines	0,06	0,11	0,02	0,01
**C2**	acylcarnitines	3,89	5,36	1,63	2,11
**C3**	acylcarnitines	0,33	0,39	0,07	0,08
**C4**	acylcarnitines	0,40	0,42	0,11	0,09
**Arg**	aminoacids	87,13	56,06	25,90	20,79
**Gln**	aminoacids	812,25	536,50	108,96	97,91
**Gly**	aminoacids	314,50	169,01	87,11	47,97
**His**	aminoacids	83,90	60,68	15,26	13,78
**Met**	aminoacids	33,20	21,99	8,75	5,64
**Orn**	aminoacids	149,00	75,02	47,24	23,79
**Phe**	aminoacids	75,28	54,10	16,81	9,60
**Pro**	aminoacids	334,25	182,32	43,22	62,33
**Ser**	aminoacids	150,93	81,55	44,47	16,01
**Thr**	aminoacids	211,90	108,25	78,01	29,21
**Trp**	aminoacids	56,73	51,80	11,35	9,23
**Tyr**	aminoacids	67,00	60,48	16,49	18,40
**Orn/Arg ratio**	aminoacids	1,71	1,34		
**Thy/Phe**	aminoacids	0,89	1,12		
**lysoPC a C16:**	glycerophospholipids	155,00	61,92	47,53	16,65
**lysoPC a C16:**	glycerophospholipids	3,66	2,20	1,16	0,98
**lysoPC a C17:**	glycerophospholipids	3,13	1,11	0,88	0,31
**lysoPC a C18:**	glycerophospholipids	52,28	18,93	14,55	4,83
**lysoPC a C18:**	glycerophospholipids	38,68	13,82	12,37	4,52
**lysoPC a C18:**	glycerophospholipids	60,05	21,61	19,30	5,35
**lysoPC a C20:**	glycerophospholipids	4,32	1,94	1,74	0,42
**lysoPC a C20:**	glycerophospholipids	11,51	5,13	4,53	1,32
**lysoPC a C24:**	glycerophospholipids	0,29	0,50	0,03	0,07
**lysoPC a C26:**	glycerophospholipids	0,27	0,13	0,06	0,03
**lysoPC a C28:**	glycerophospholipids	0,90	0,35	0,17	0,07
**PC aa C28:1**	glycerophospholipids	4,39	2,27	0,48	0,56
**PC aa C30:0**	glycerophospholipids	4,04	5,34	0,47	1,51
**PC aa C32:0**	glycerophospholipids	19,35	15,69	1,97	2,92
**PC aa C32:1**	glycerophospholipids	16,93	23,37	3,37	12,91
**PC aa C32:2**	glycerophospholipids	4,67	4,94	1,11	1,61
**PC aa C32:3**	glycerophospholipids	0,62	0,54	0,12	0,09
**PC aa C34:1**	glycerophospholipids	217,50	208,89	22,05	48,82
**PC aa C34:2**	glycerophospholipids	372,00	382,05	40,23	49,75
**PC aa C34:3**	glycerophospholipids	17,90	17,21	2,48	3,88
**PC aa C34:4**	glycerophospholipids	2,03	1,99	0,66	0,57

**PC aa C36:0**	glycerophospholipids	2,26	1,47	0,45	0,45
**PC aa C36:1**	glycerophospholipids	53,43	42,97	8,34	10,48
**PC aa C36:2**	glycerophospholipids	234,00	219,81	26,85	27,99
**PC aa C36:3**	glycerophospholipids	151,75	141,21	16,32	23,60
**PC aa C36:4**	glycerophospholipids	208,25	194,34	30,46	28,59
**PC aa C36:5**	glycerophospholipids	24,03	22,27	5,17	7,42
**PC aa C36:6**	glycerophospholipids	0,89	0,94	0,23	0,28
**PC aa C38:0**	glycerophospholipids	2,90	2,18	0,46	0,59
**PC aa C38:1**	glycerophospholipids	0,72	0,92	0,29	0,39
**PC aa C38:3**	glycerophospholipids	52,50	42,26	8,32	9,48
**PC aa C38:4**	glycerophospholipids	116,45	100,35	24,78	16,44
**PC aa C38:5**	glycerophospholipids	58,25	49,85	10,68	8,94
**PC aa C38:6**	glycerophospholipids	82,13	70,40	8,52	15,92
**PC aa C40:2**	glycerophospholipids	0,21	0,26	0,05	0,06
**PC aa C40:3**	glycerophospholipids	0,39	0,44	0,03	0,06
**PC aa C40:4**	glycerophospholipids	2,60	2,92	0,39	0,60
**PC aa C40:5**	glycerophospholipids	7,68	8,14	1,20	2,02
**PC aa C40:6**	glycerophospholipids	23,20	20,93	2,81	5,34
**PC aa C42:0**	glycerophospholipids	0,35	0,42	0,05	0,11
**PC aa C42:1**	glycerophospholipids	0,16	0,21	0,03	0,06
**PC aa C42:2**	glycerophospholipids	0,12	0,18	0,02	0,04
**PC aa C42:4**	glycerophospholipids	0,11	0,17	0,02	0,03
**PC aa C42:6**	glycerophospholipids	0,27	0,57	0,04	0,06
**PC ae C30:0**	glycerophospholipids	0,39	0,67	0,08	0,13
**PC ae C30:2**	glycerophospholipids	0,13	0,09	0,02	0,02
**PC ae C32:1**	glycerophospholipids	3,36	2,59	0,56	0,61
**PC ae C32:2**	glycerophospholipids	0,95	0,63	0,14	0,16
**PC ae C34:0**	glycerophospholipids	1,83	1,78	0,35	0,40
**PC ae C34:1**	glycerophospholipids	13,33	9,09	1,88	2,10
**PC ae C34:2**	glycerophospholipids	19,00	10,39	2,21	1,98
**PC ae C34:3**	glycerophospholipids	11,49	6,44	1,94	1,30
**PC ae C36:0**	glycerophospholipids	0,81	0,98	0,12	0,18
**PC ae C36:1**	glycerophospholipids	9,72	7,09	0,93	1,65
**PC ae C36:2**	glycerophospholipids	18,38	12,44	2,09	2,83
**PC ae C36:3**	glycerophospholipids	10,70	6,48	1,24	1,05
**PC ae C36:4**	glycerophospholipids	27,78	15,23	3,40	3,10
**PC ae C36:5**	glycerophospholipids	15,58	9,65	2,42	1,80
**PC ae C38:0**	glycerophospholipids	1,79	2,04	0,26	0,47
**PC ae C38:1**	glycerophospholipids	0,41	0,61	0,26	0,30
**PC ae C38:2**	glycerophospholipids	2,06	1,64	0,11	0,29
**PC ae C38:3**	glycerophospholipids	4,78	3,51	0,97	0,78
**PC ae C38:4**	glycerophospholipids	17,88	10,88	3,95	2,39
**PC ae C38:5**	glycerophospholipids	24,85	14,41	3,56	2,80
**PC ae C38:6**	glycerophospholipids	8,96	5,88	1,13	1,36
**PC ae C40:1**	glycerophospholipids	1,23	1,09	0,17	0,27
**PC ae C40:2**	glycerophospholipids	1,82	1,22	0,14	0,24
**PC ae C40:3**	glycerophospholipids	0,92	0,92	0,12	0,23
**PC ae C40:4**	glycerophospholipids	2,16	1,78	0,32	0,47
**PC ae C40:5**	glycerophospholipids	3,50	2,80	0,62	0,75
**PC ae C40:6**	glycerophospholipids	4,58	3,30	0,55	0,73

**PC ae C42:2**	glycerophospholipids	0,42	0,39	0,05	0,08
**PC ae C42:3**	glycerophospholipids	0,53	0,78	0,08	0,13
**PC ae C44:3**	glycerophospholipids	0,07	0,83	0,02	0,08
**PC ae C44:5**	glycerophospholipids	1,16	1,05	0,28	0,37
**PC ae C44:6**	glycerophospholipids	0,72	0,74	0,13	0,26
**SM (OH) C14:**	sphingolipids	8,76	6,33	1,29	1,46
**SM (OH) C16:**	sphingolipids	3,92	3,49	0,56	0,81
**SM (OH) C22:**	sphingolipids	8,71	9,84	1,24	1,70
**SM (OH) C22:**	sphingolipids	9,73	8,10	1,53	1,95
**SM (OH) C24:**	sphingolipids	0,74	0,95	0,12	0,20
**SM C16:0**	sphingolipids	109,33	115,76	15,77	14,07
**SM C16:1**	sphingolipids	18,58	15,81	2,69	2,60
**SM C18:0**	sphingolipids	24,25	22,52	3,27	5,15
**SM C18:1**	sphingolipids	11,46	10,66	1,88	2,65
**SM C20:2**	sphingolipids	0,71	0,51	0,07	0,11
**SM C22:3**	sphingolipids	5,39	0,19	1,25	0,31
**SM C24:0**	sphingolipids	13,03	15,27	1,54	1,86
**SM C24:1**	sphingolipids	36,88	35,46	4,93	5,58
**SM C26:0**	sphingolipids	0,12	0,11	0,03	0,04
**SM C26:1**	sphingolipids	0,26	0,23	0,06	0,08
**H1**	sugars	5695,25	4589,39	588,41	890,44

Difference FSGS patient at time of disease recurrence versus healthy controls
p-Wert (red, p< 0,05)
8,2E-01
1,4E-02
8,3E-03
1,8E-01
1,5E-01
7,3E-01
9,0E-02
9,2E-03
4,0E-02
4,7E-02
7,8E-02
4,9E-02
8,2E-02
1,1E-03
5,1E-02
7,5E-02
4,6E-01
5,1E-01
2,8E-02
8,0E-02
1,8E-02
1,8E-02
2,6E-02
2,7E-02
7,1E-02
6,6E-02
9,3E-06
1,8E-02
6,7E-03
6,2E-04
5,6E-03
2,1E-02
6,9E-02
7,0E-01
3,4E-01
5,9E-01
6,8E-01
6,6E-01
9,2E-01

2,9E-02
7,9E-02
3,9E-01
3,2E-01
4,5E-01
5,9E-01
7,3E-01
4,0E-02
2,9E-01
8,3E-02
2,9E-01
2,2E-01
6,7E-02
1,1E-01
8,2E-02
2,2E-01
5,6E-01
2,5E-01
1,2E-01
4,7E-02
5,4E-03
7,0E-03
5,5E-06
9,7E-04
1,0E-02
6,2E-02
1,1E-02
8,1E-01
1,1E-02
1,9E-03
1,0E-02
4,3E-02
2,6E-03
3,6E-03
3,4E-03
2,3E-03
1,2E-02
1,6E-01
2,3E-01
2,1E-04
7,2E-02
3,4E-02
6,1E-03
5,0E-03
2,4E-01
2,2E-04
9,7E-01
9,8E-02
1,1E-01
8,6E-03

2,8E-01
2,0E-03
2,9E-21
5,0E-01
8,8E-01
2,3E-02
2,5E-01
1,8E-01
1,2E-01
2,1E-02
4,9E-01
1,3E-01
4,2E-01
5,0E-01
2,7E-03
3,4E-03
5,1E-02
6,3E-01
6,5E-01
3,4E-01
2,0E-02
